# *ortho*-Naphthoquinone-catalyzed aerobic oxidation of amines to fused pyrimidin-4(3*H*)-ones: a convergent synthetic route to bouchardatine and sildenafil†

**DOI:** 10.1039/d0ra06820a

**Published:** 2020-08-21

**Authors:** Kyeongha Kim, Hun Young Kim, Kyungsoo Oh

**Affiliations:** Center for Metareceptome Research, Graduate School of Pharmaceutical Sciences, Chung-Ang University 84 Heukseok-ro, Dongjak Seoul 06974 Republic of Korea kyungsoooh@cau.ac.kr

## Abstract

A facile access to fused pyrimidin-4(3*H*)-one derivatives has been established by using the metal-free *ortho*-naphthoquinone-catalyzed aerobic cross-coupling reactions of amines. The utilization of two readily available amines allowed a direct coupling strategy to quinazolinone natural product, bouchardatine, as well as sildenafil (Viagra™) in a highly convergent manner.

## Abstract 2

Fused pyrimidin-4(3*H*)-one derivatives have been accessed by using the *ortho*-naphthoquinone-catalyzed aerobic cross-coupling reactions of amines.

## Graphical abstract

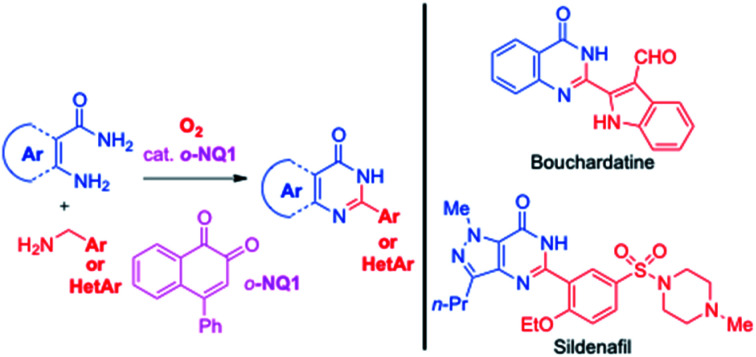

N-Heterocyclic compounds with a pyrimidin-4(3*H*)-one core constitute a large number of natural products and biologically active molecules. For example, quinazolinone alkaloids possess a phenyl-fused pyrimidin-4(3*H*)-one structure and display a wide spectrum of pharmacological activities (Scheme 1a).^1^ Sildenafil (Viagra™), a potent and selective inhibitor of type 5 phosphodiesterases on smooth muscle cell, is based on the pyrazole-fused pyrimidin-4(3*H*)-one structure and marketed for erectile dysfunction.^2^ The synthetic approaches to the phenyl-fused pyrimidin-4(3*H*)-ones, quinazolinones, typically involve the condensation between anthranilamides and aldehydes to give aminal intermediates that in turn oxidized to quinazolinones under oxidation conditions (Scheme 1b). The oxidation catalysts include Cu,^3^ Fe,^4^ Ga,^5^ Ir,^6^ Mn,^7^ iodine,^8^ peroxide,^9^ however the aerobic oxidation of aminal intermediates is also known at 150 °C.^10^ The utilization of alcohols also effects the one-pot synthesis of quinazolinones through *in situ* oxidation to aldehydes in the presence of Fe,^11^ Ir,^12^ Mn,^13^ Ni,^14^ Pd,^15^ Ru,^16^ V,^17^ Zn,^18^ and iodine catalysts.^19^ Other precursors to aldehydes have been also identified using alkynes,^20^ benzoic acids,^21^ indoles,^22^ α-keto acid salts,^23^ β-keto esters,^24^ styrenes,^25^ sulfoxides,^26^ and toluenes.^27^ Non-aldehyde approaches to quinazolinones have been also demonstrated in the cross coupling of amidines,^28^ amines,^29^ benzamides,^30^ isocyanides,^31^ and nitriles.^32^ In 2013, the Nguyen group disclosed the synthesis of four quinazolinones, utilizing the autooxidation of benzylamines to imines that subsequently condensed with anthranilamides.^33^ While a closed system at 150 °C was necessary, the use of 40 mol% AcOH without solvent provided the quinazolinones in 46–75% yields. The Nguyen group also developed the FeCl_3_·6H_2_O-catalyzed condensation of 2-nitroanilines and benzylamines in the presence of 20 mol% of S_8_, where six quinazolinones were obtained in 68–75% yields.^34^ While the cross condensation of anthranilamides and benzylamines was accomplished, there exists a significant knowledge gap due to the limited substrate scope combined with less optimal reaction conditions (*i.e.* high reaction temperature, closed system in neat conditions and excess use of amines). In addition, it is not entirely clear if the cross condensation of amide-containing amines and benzylamines would work for other fused pyrimidin-4(3*H*)-one derivatives.^35^ To address such shortcomings in the cross condensation of amines, the *ortho*-naphthoquinone (*o*-NQ)-catalyzed aerobic cross amination strategy was investigated (Scheme 1c).^36^ Herein, we report a highly general approach to fused pyrimidin-4(3*H*)-one derivatives in the presence of *o*-NQ catalyst, culminating to the direct aerobic coupling of two amines to bouchardatine and sildenafil.

**Scheme 1:**
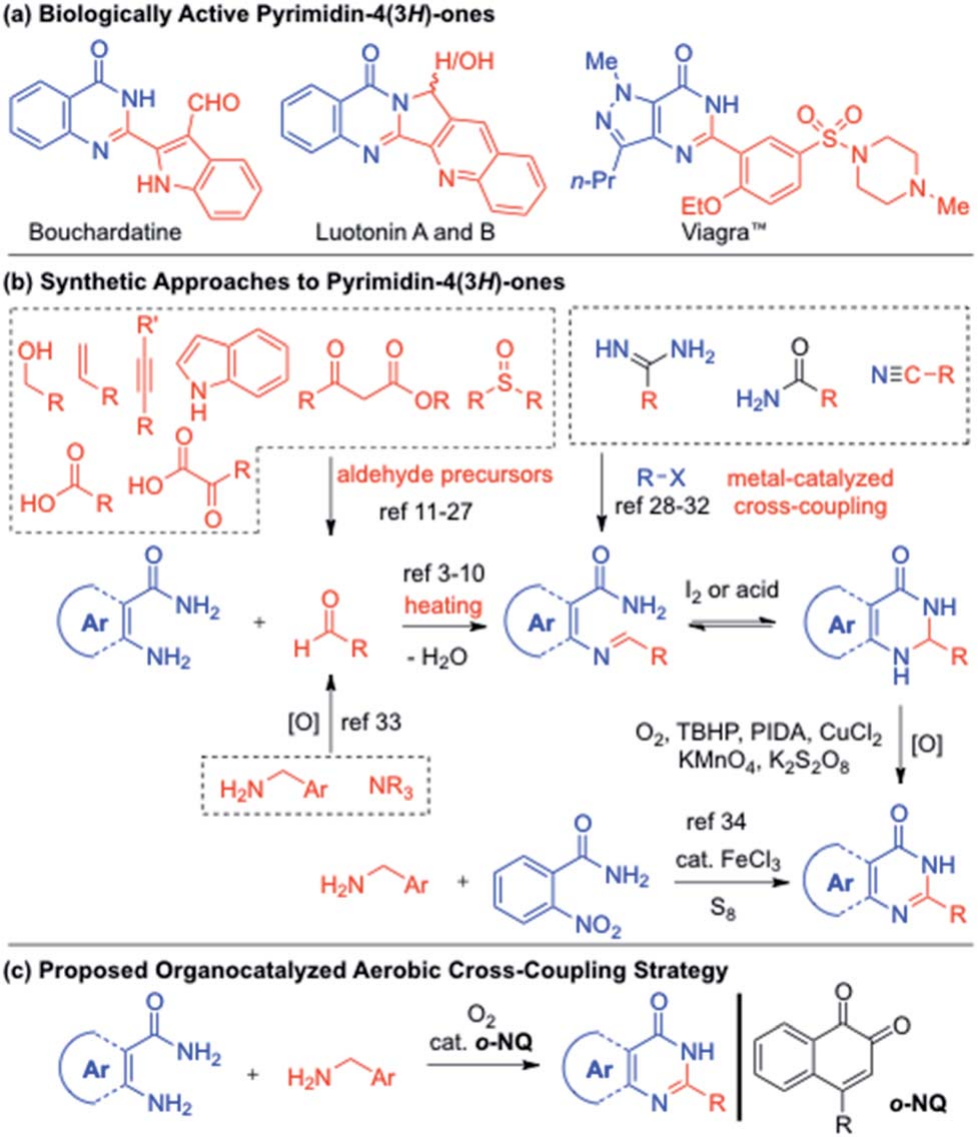
Biologically active fused pyrimidin-4(3*H*)-one derivatives and their synthetic methods.

Given that the *o*-NQ-catalyzed aerobic cross coupling of benzylamines and aniline derivatives such as *o*-phenylenediamines provided a facile approach to heterocyclic compounds including benzoimidazoles,^37^ the use of anthranilamide 1a and benzylamine 2a was examined as a model study (Table 1). The catalytic use of *o*-NQ1 smoothly converted benzylamine 2a to the corresponding homocoupled imine 2a′ under aerobic conditions. However, the subsequent *in situ* condensation reaction of 2a′ with anthranilamide 1a only provided the aminal product 3a in 11% yield (entry 1). To facilitate the cross coupling between the amine 2a and anthranilamide 1a, a catalytic amount of TFA was utilized where a significant improvement in yield was observed for 3a (entry 2). Faced with the inability to oxidize the aminal 3a to the corresponding product, quinazolinone 4a, other *ortho*-naphthoquinone catalysts were screened without much success (entries 3–5). To our delight, the examination of solvents revealed that the reaction temperature of 100 °C was needed for the formation of 4a (entries 6–11).^38^ The reaction in DMSO lowered down the ratio of 1a and 2a from 1.0 : 1.5 to 1.0 : 1.2 (entry 12) and the catalyst loading to 5 mol% without affecting the overall reaction efficiency (entry 13). The control experiments confirmed the critical roles of both *o*-NQ1 and TFA (entries 15–18), and the reaction utilized molecular oxygen as a terminal oxidant (entries 19 and 20). Piecing together the experimental data, the employment of 5 mol% *o*-NQ1 and 20 mol% TFA in DMSO at 100 °C was selected for further studies.

**Table 1:** Optimization of *o*-NQ-catalyzed aerobic cross-coupling of amines to quinazolinone^a^

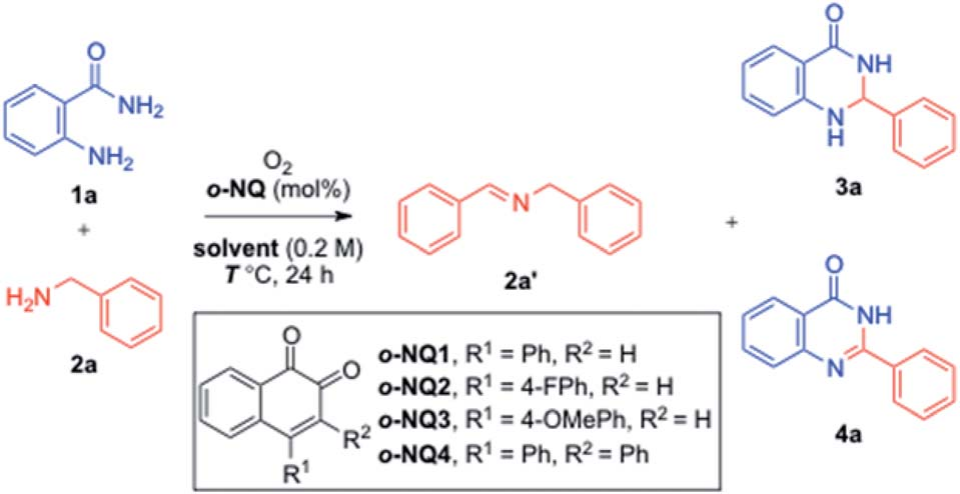				
Entry	Cat. (mol%)	Solvent	*T* (°C)	Yield^b^ (%)
1	*o*-NQ1 (10)	CH_3_CN	80	3a, 11
2	*o*-NQ1 (10)/TFA (20)	CH_3_CN	80	3a, 83
3	*o*-NQ2 (10)/TFA (20)	CH_3_CN	80	3a, 13
4	*o*-NQ3 (10)/TFA (20)	CH_3_CN	80	3a, 66
5	*o*-NQ4 (10)/TFA (20)	CH_3_CN	80	3a, 32
6	*o*-NQ1 (10)/TFA (20)	MeOH	65	3a, 5
7	*o*-NQ1 (10)/TFA (20)	EtOH	78	3a, 31
8	*o*-NQ1 (10)/TFA (20)	DMF	150	4a, >95
9^c^	*o*-NQ1 (10)/TFA (20)	DMSO	150	4a, >95
10	*o*-NQ1 (10)/TFA (20)	DMSO	100	4a, >95
11	*o*-NQ1 (10)/TFA (20)	DMSO	80	3a, 30
12^d^	*o*-NQ1 (10)/TFA (20)	DMSO	100	4a, >95
13^d^	*o*-NQ1 (5)/TFA (20)	DMSO	100	4a, >95 (93)
14	*o*-NQ1 (5)/TFA (10)	DMSO	100	3a/4a, 45/50
15	—	DMSO	100	3a, 10
16	*o*-NQ1 (10)	DMSO	100	3a/4a, 34/51
17	TFA (20)	DMSO	100	NR
18	*o*-NQ1 (5)/AcOH (20)	DMSO	100	3a, 25
19^e^	*o*-NQ1 (5)/TFA (20)	DMSO	100	3a, 33
20^f^	*o*-NQ1 (5)/TFA (20)	DMSO	100	NR

a Reaction using 1a (0.20 mmol), 2a (0.30 mmol), and *o*-NQ in solvent (0.2 M) under O_2_ balloon for 24 h.

b Yields based on internal standard and isolated yield in parentheses.

c Reaction for 6 h.

d Use of 2a (0.24 mmol, 1.2 equiv.).

e Reaction under air.

f Reaction under argon. NR = no reaction.

The optimized aerobic cross-coupling condition was applied to a variety of benzylamine derivatives (Scheme 2). In general, the electronic and steric characters of benzylamines did not significantly affect the formation of quinazolinones (4a–4m). However, the use of halogen-substituted and dimethoxy-substituted benzylamines led to the slightly lower yields of quinazolinones (4e, 4f and 4m) in 58–75% yields. In addition, the current aerobic cross-coupling reaction tolerated the furanyl and thiophenyl moieties, where the corresponding quinazolinones (4o and 4p) were obtained in 54% and 75% yields, respectively.

**Scheme 2:**
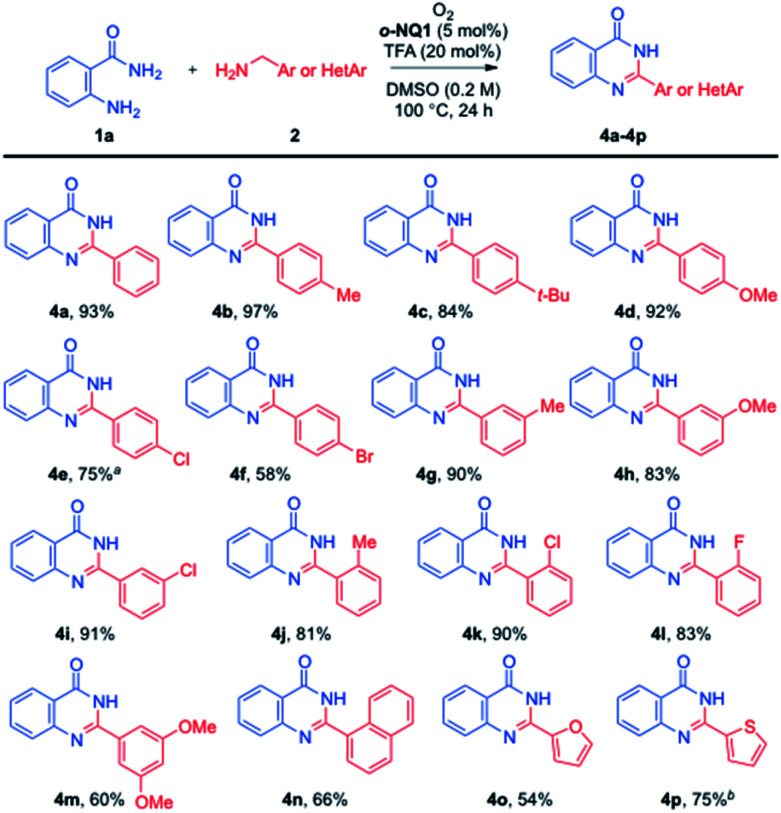
Substrate scope of aerobic oxidation to quinazolinones (^*a*^reaction for 36 h, ^*b*^reaction for 12 h).

Further extension of the current aerobic cross-coupling reactions of amines is illustrated in Scheme 3. Thus, an array of substituted anthranilamides was readily employed to give the fused pyrimidin-4(3*H*)-one derivatives (4q–4x) in 61–84% yields. In particular, the *N*-substituted anthranilamides also participated in the current aerobic cross-coupling reaction in excellent yields (4y–4za). While the use of 3-amino-2-naphthamide led to the corresponding quinazolinone 4zb in 46% yield, the synthetic advantage of the current method was well demonstrated in the preparation of heteroaryl fused pyrimidin-4(3*H*)-one derivatives (4zc–4zh), where a variety of heterocyclic amines were successfully utilized in a tandem sequence of aerobic oxidation processes.

**Scheme 3:**
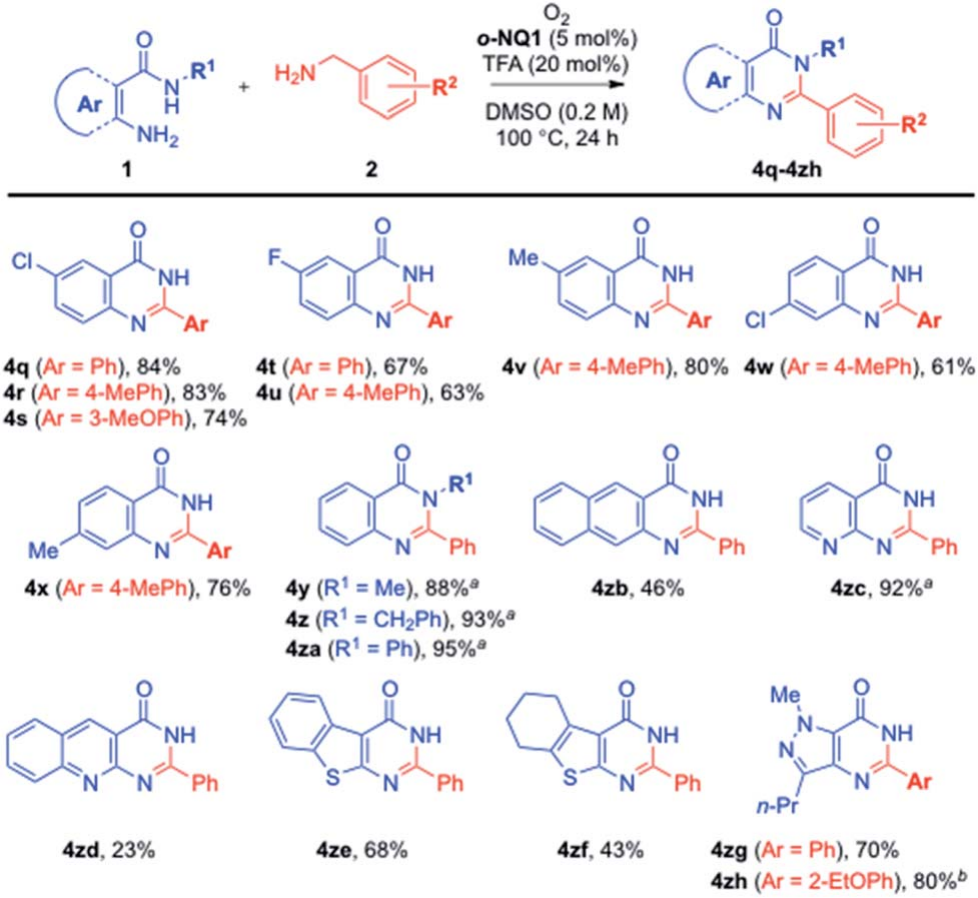
Further substrate scope for fused pyrimidin-4(3*H*)-one derivatives (^*a*^reaction at 120 °C, ^*b*^reaction at 140 °C).

The mechanistic rationale of the aerobic cross-coupling reactions of amines is depicted in Scheme 4. Thus, the benzylamine 2a is condensed with the *o*-NQ1 catalyst to give the naphthol-imine species A.^37*a*^ While the nucleophilic attack of 2a to the naphthol-imine A is favored due to the low nucleophilicity of the anthranilamide 1a, the presence of TFA promotes the cross-coupling between naphthol-imine A and anthranilamide 1a to give the naphthol-aminal B1. This process releases the hetero-coupled imine 3a′ and naphthol-amine C. The use of TFA promotes the intramolecular Mannich cyclization of imine 3a′, leading to the aminal 3a that in turn converts to the desired fused pyrimidin-4(3*H*)-one 4a with the help of *o*-NQ1 catalyst and molecular oxygen. Alternatively, the naphthol-imine A can produce the homocoupled imine 2a′ and the naphthol-amine C*via* the naphthol-aminal B2 through the nucleophilic attack of benzylamine 2a. The conversion of the naphthol-amine C to *o*-NQ1 catalyst is effected upon exposure to oxygen atmosphere.^38^ The homocoupled imine 2a′ undergoes hydrolysis at >80 °C to the benzaldehyde and benzylamine 2a that in turn re-enters the catalytic cycle.^37*b*^ Our experimental observation of the homocoupled imine 2a′ by the ^1^H NMR and TLC analysis during the reaction supports the involvement of 2a′. However, the major pathway to the fused pyrimidin-4(3*H*)-one 4a appears to involve the naphthol-aminal B1 since the use of benzaldehyde instead of benzylamine 2a under the optimized conditions only led to the 80% conversion. The *o*-NQ1 catalyst without added TFA provided a mixture of aminal 3a and quinazolinone 4a in 34% and 51% yields, respectively (Table 1, entry 16). Thus, it is likely that the role of TFA is the catalyst for the cross-coupling of two amines to give the naphthol-aminal B1 and the cyclization of the imine 3a′ to aminal 3a. Our control experiments also revealed that TFA alone slowly oxidize 3a to 4a, but rapidly oxidized by the action of *o*-NQ1 within 10 h.^39^

**Scheme 4:**
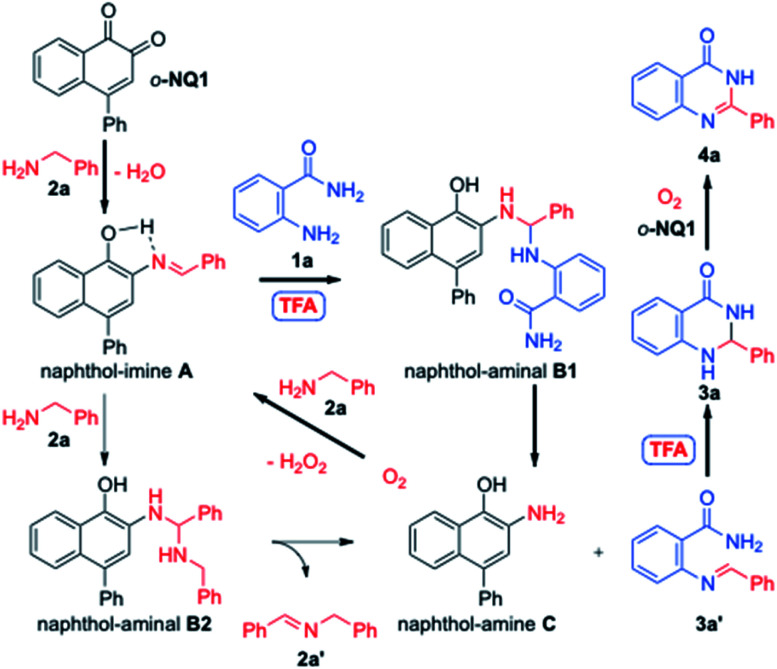
Mechanistic rationale for aerobic cross coupling reaction of amines.

The synthetic utility of the aerobic cross-coupling strategy is demonstrated in the synthesis of quinazolinone alkaloids and sildenafil (Scheme 5). The direct cross coupling of a commercially available pyrazole amine 1o and benzylamine 2r afforded a highly convergent synthetic approach to sildenafil.^40^ Likewise, the employment of anthranilamide 1a and 2-(aminomethyl)indole 2s provided the desired quinazolinone 5 in 70% yield, and the subsequent formylation under the Zeng's conditions^41^ paved a way to the total synthesis of bouchardatine. In addition, while the basicity of quinolin-2-ylmethanamine 2t required an excess of TFA, the corresponding quinazolinone 6 was obtained in 60% yield under the optimized conditions. The conversion of 6 to the luotonin natural products has been reported by the Argade group and others.^42^

**Scheme 5:**
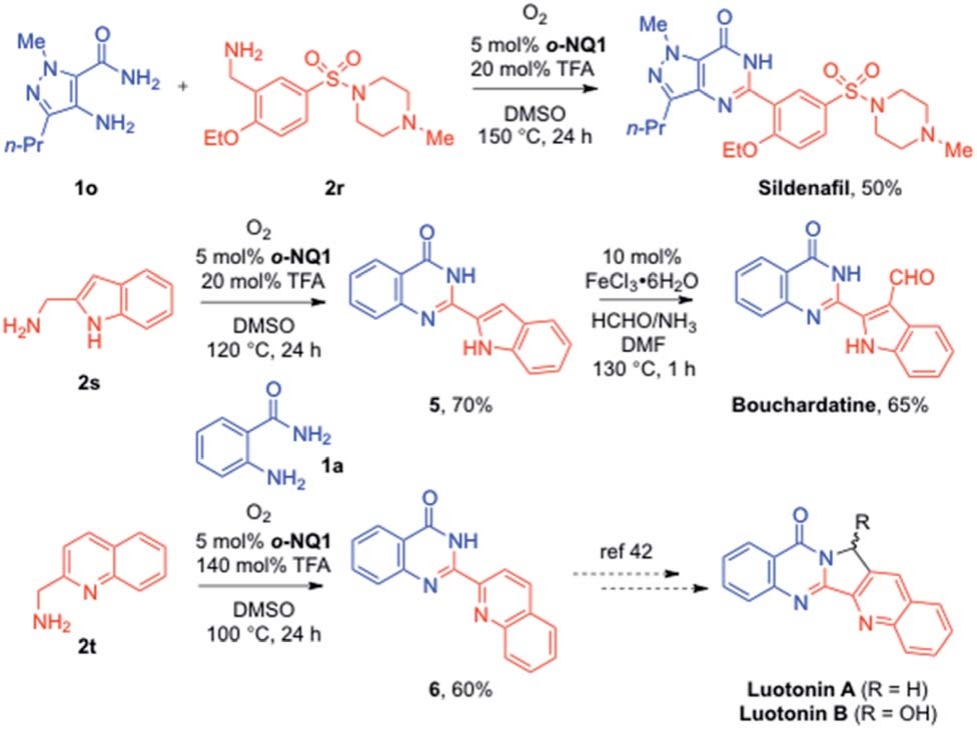
Synthetic utilization to quinazolinone alkaloids and sildenafil.

## Conclusions

In summary, we have developed the aerobic cross-coupling reactions of amines to fused pyrimidin-4(3*H*)-one derivatives. This metal-free tandem aerobic oxidation sequence utilizes 5 mol% of *o*-NQ catalyst and 20 mol% of TFA as co-catalyst. The developed aerobic oxidation protocol allows a highly convergent approach to quinazolinone alkaloids and sildenafil. Given that the fused pyrimidin-4(3*H*)-one derivatives possess a diverse array of biological activities, the *o*-NQ-catalyzed tandem aerobic cross-coupling reactions should find their synthetic utility in the medicinal chemistry projects. We are current extending the *o*-NQ-catalyzed aerobic oxidation protocols to other heterocycles of medicinal interest, and our results will be reported in due course.

## Conflicts of interest

There are no conflicts to declare.

## Supplementary Material

RA-010-D0RA06820A-s001
