# Supplementary material for: ortho-Naphthoquinone-catalyzed aerobic oxidation of amines to fused pyrimidin-4(3H)-ones: a convergent synthetic route to bouchardatine and sildenafil
Source: RSC Adv. 2020 Aug 21;10(52):31101–5. doi: 10.1039/d0ra06820a (PMC9056348; doi:10.1039/d0ra06820a)

Electronic Supplementary Material (ESI) for RSC Advances.
This journal is © The Royal Society of Chemistry 2020

ortho-Naphthoquinone-Catalyzed Aerobic Oxidation of Amines to Fused Pyrimidin-4(3*H*)-ones: A Convergent Synthetic Route to Bouchardatine and Sildenafil

Kyeongha Kim, Hun Young Kim and Kyungsoo Oh*

Center for Metareceptome Research, Graduate School of Pharmaceutical Sciences, Chung-Ang University, 84 Heukseok-ro, Dongjak, Seoul 06974, Republic of Korea

Table of Contents

General Methods S2

2-Amino-aryl Benzamide Starting Materials S2

Aryl Amine Starting Materials S5

Preparation of *ortho*-Naphthoquinones Catalysts S6

General Procedure A for the Synthesis of Fused Pyrimidin-4(3*H*)-ones: S6

1.0 mmol Scale Reaction S6

Characterization of the Products in Table 1, Scheme 2 and 3 S7

Synthesis of Bouchardatine in Scheme 5 S17

Synthesis of Sildenafil in Scheme 5 S18

References S20

^{1}H & ^{13}C NMR Spectra S23

General Methods

All reactions were carried out with oven-dried glassware. The progress of all reactions was monitored by thin-layer chromatography on Dynamic Adsorbent, Inc. precoated silica gel plates (250 μm) and visualized by ultra-violet light or by staining with $KMnO_4$ stain. HPLC grade solvents were used without further drying. Unless otherwise specified, all chemicals were purchased from Acros or Alfa Aesar or TCI and all solvents were purchased from Fischer Scientific. The microwave assisted reactions were carried out in Anton Paar microwave 400 synthesis reactor. ^{1}H and ^{13}C NMR spectra were recorded in $CDCl_3$ and DMSO-d_6 on JEOL 600 MHz Fourier transform spectrometers at ambient temperature. The coupling constant *J* is given in Hz. The chemical shifts are reported in ppm on a scale downfield from TMS as an internal standard, and signal patterns are indicated as follows: s = singlet, d = doublet, t = triplet, q = quartet, m = multiplet. For high resolution mass spectrometry (HRMS), m/z ratios are reported as values in atomic mass units. Silica gel (32-64u, Dynamic Adsorbent, Inc.) was used for column chromatography. The infrared spectra were obtained using a Aglient Cary 630 FRIR Spectrometer. Melting points were recorded on a Buchi-B-450 melting point apparatus and the values were uncorrected.

2-Amino-aryl Benzamide Starting Materials

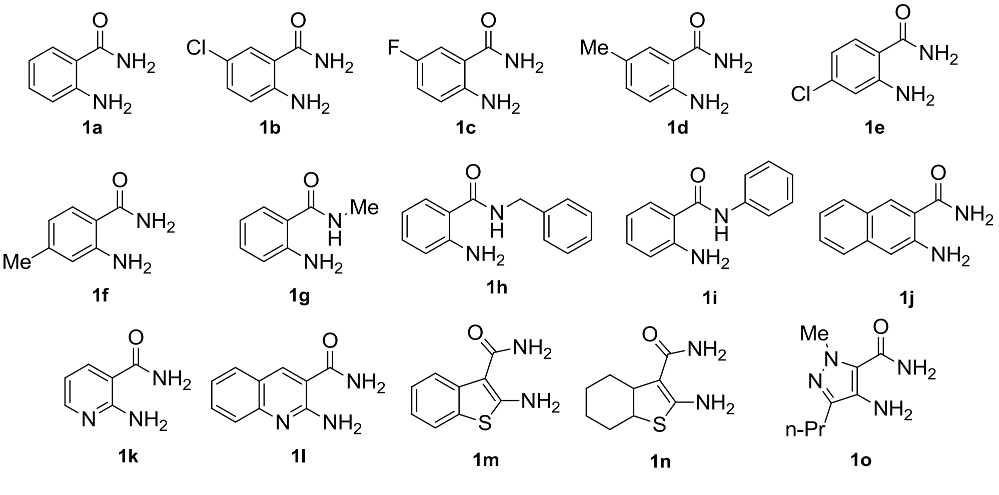

2-Amino-aryl benzamide **1a**-**1c**, **1k**, **1n** and **1o** are commercially available. The compounds **1j**, **1l** are prepared according to literature procedure and the ^{1}H and ^{13}C NMR spectra were compared with previously reported literature data.[1-2] **1d**-**1i** and **1m** were synthesized based on the literature procedure with slight modification.[3-4]

1) Synthesis of 2-Amino-aryl Benzamides (Method A for 1d, 1e, 1f and 1m)

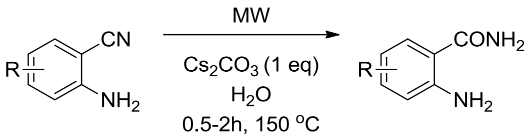

Benzonitrile compounds (1.0 mmol) and Cs_2CO_3 (1.0 equiv) were added into a 10 mL microwave reaction vial and 8.5 mL of deionized water was added. After irradiation under microwave at 150 °C for 25 min-2 h, the reaction mixture was cooled down and concentrated under reduced pressure. The residue was dissolved in acetone, dried over Na_2SO_4, filtered and concentrated under reduced pressure. The residue was purified by flash column chromatography on silica gel (eluent: 2-5% MeOH in DCM) to give **1d-1f, 1m** in 70-90% yields.

2) Synthesis of 2-Amino-aryl Benzamides (Method B for 1g-1i)

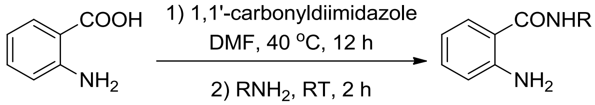

To a flask charged with anthranilic acid (1.0 mmol) and 1,1'-carbonyldiimidazole (1.0 equiv), was added 4 mL of anhydrous DMF under argon. The reaction was stirred at 40 °C for 12 h. After cooling down to ambient temperature, amine (1.0 equiv) was added and reaction was further stirred for 2 h. The reaction mixture was extracted with ethyl acetate (20 mL x 3) and washed with brine, dried over Na_2SO_4, filtered and concentrated under reduced pressure. The residue was purified by flash column chromatography on silica gel (eluent: 20% ethyl acetate in hexanes) to give **1g-1i** in 67-92% yields.

3) Characterization of Aryl Benzamide Starting Materials:

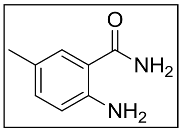

2-Amino-5-methylbenzamide (1d): The product **1d** was prepared by the Method A using 2-amino-5-methylbenzonitrile (132.1 mg, 1.0 mmol). The pure product was obtained by column chromatography on silica gel using 3% MeOH in CH_2Cl_2 as eluent. 130.4 mg (80%); Pale yellow solid; The ^1H and ^{13}C NMR spectra for this compound are consistent with the previously reported literature.[5] ^1H-NMR (DMSO-d_6, 600 MHz): δ 7.62 (s, 1H), 7.31 (d, J = 1.8 Hz, 1H), 6.96 (s, 1H), 6.92 (dd, J = 8.4, 1.8 Hz, 1H), 6.55 (d, J

= 8.4 Hz, 1H), 6.28 (s, 2H), 2.11 (s, 3H); ^{13}C-NMR (DMSO-d_6, 150 MHz): δ 171.3, 147.8, 132.7, 128.6, 122.7, 116.5, 113.7, 20.0.

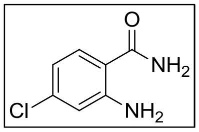

2-Amino-4-chlorobenzamide (1e): The product **1e** was prepared by the Method A using 2-amino-4-chlorobenzonitrile (152.5 mg, 1.0 mmol). The pure product was obtained by column chromatography on silica gel using 2% MeOH in CH_2Cl_2 as eluent. 153.5 mg (90%); Pale yellow solid; The ^{1}H and ^{13}C NMR spectra for this compound are consistent with the previously reported literature.[6] ^{1}H-NMR (DMSO-d_6, 600 MHz): δ 7.77 (s, 1H), 7.53 (d, J = 8.4 Hz, 1H), 7.14 (s, 1H), 6.82 (s, 2H), 6.73 (d, J = 1.8 Hz, 1H), 6.47 (dd, J = 8.4, 1.8 Hz, 1H); ^{13}C-NMR (DMSO-d_6, 150 MHz): δ 170.4, 151.5, 136.3, 130.6, 115.1, 114.0, 112.4.

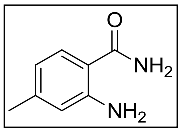

2-Amino-4-methylbenzamide (1f): The product **1f** was prepared by the Method A using 2-amino-4-methylbenzonitrile (132.1 mg, 1.0 mmol). The pure product was obtained by column chromatography on silica gel using 3% MeOH in CH_2Cl_2 as eluent. 112.5 mg (75%); White solid; The ^{1}H and ^{13}C NMR spectra for this compound are consistent with the previously reported literature.[7] ^{1}H-NMR (DMSO-d_6, 600 MHz): δ 7.61 (s, 1H), 7.42 (d, J = 8.4 Hz, 1H), 6.92 (s, 1H), 6.52 (s, 2H), 6.46 (s, 1H), 6.28 (d, J = 8.4 Hz, 1H), 2.15 (s, 3H); ^{13}C-NMR (DMSO-d_6, 150 MHz): δ 171.2, 150.3, 141.6, 128.8, 116.4, 115.6, 111.1, 21.0.

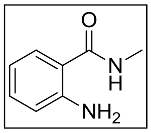

2-Amino-N-methylbenzamide (1g): The product **1g** was prepared by the Method B using anthranilic acid (137.1 mg, 1.0 mmol) and methyl amine (40 wt% solution in water, 83 µL, 1.0 mmol). The pure product was obtained by column chromatography on silica gel using 20% ethyl acetate in hexanes as eluent. 100.6 mg (67%); White solid; The ^{1}H and ^{13}C NMR spectra for this compound are consistent with the previously reported literature.[8] ^{1}H-NMR ($CDCl_3$, 600 MHz): δ 7.27-7.29 (m, 1H), 7.20-7.17 (m, 1H), 6.67-6.61 (m, 2H), 6.15 (s, 1H), 5.49 (s, 2H), 2.94 (d, J = 4.8 Hz, 3H); ^{13}C-NMR ($CDCl_3$, 150 MHz): δ 170.1, 148.7, 132.3, 127.2, 117.4, 116.7, 116.4, 26.6.

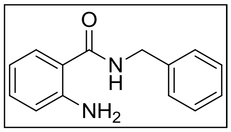

2-Amino-N-benzylbenzamide (1h): The product **1h** was prepared by the Method B using anthranilic acid (137.1 mg, 1.0 mmol) and benzylamine (109 µL, 1.0 mmol). The pure product was obtained by column chromatography on silica gel using 20% ethyl acetate in hexanes as eluent. 203.6 mg (90%); White solid; The ^{1}H and ^{13}C NMR spectra for this compound are consistent with the previously reported literature.[8] ^{1}H-NMR ($CDCl_3$, 600 MHz): δ 7.37-7.28 (m, 6H), 7.19-7.21 (m, 1H), 6.68 (d, J = 8.4 Hz, 1H), 6.61-6.63 (m, 1H), 6.40 (s, 1H), 5.55 (s, 2H), 4.59 (d, J = 5.4 Hz, 2H); ^{13}C-NMR ($CDCl_3$, 150 MHz): δ 169.3,

149.0, 138.4, 132.5, 128.9, 127.9, 127.6, 127.2, 117.5, 116.7, 115.9, 43.8.

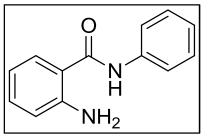

2-Amino-N-phenylbenzamide (1i): The product **1i** was prepared by the Method B using anthranilic acid (137.1 mg, 1.0 mmol) and aniline (91 μL, 1.0 mmol). The pure product was obtained by column chromatography on silica gel using 20% ethyl acetate in hexanes as eluent. 195.2 mg (92%); White solid; The ^{1}H and ^{13}C NMR spectra for this compound are consistent with the previously reported literature.[8] ^{1}H-NMR ($CDCl_3$, 600 MHz): δ 7.75 (s, 1H), 7.56 (d, J = 7.8 Hz, 2H), 7.46 (d, J = 8.4 Hz, 1H), 7.36 (t, J = 7.8 Hz, 2H), 7.26-7.23 (m, 1H), 7.12-7.15 (m, 1H), 6.69-6.71 (m, 2H), 5.47 (s, 2H); ^{13}C-NMR ($CDCl_3$, 150 MHz): δ 167.7, 149.1, 138.0, 132.9, 129.2, 127.3, 124.6, 120.7, 117.7, 117.0, 116.4.

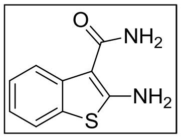

2-Aminobenzo[*b*]thiophene-3-carboxamide (1m): The product **1m** was prepared by the Method A using 2-aminobenzo[*b*]thiophene-3-carbonitrile (174.22 mg, 1.0 mmol). The pure product was obtained by column chromatography on silica gel using 5% MeOH in CH_2Cl_2 as eluent. 134.5 mg (70%); White solid, m.p. 188-191 °C; ^{1}H-NMR (DMSO-d_6, 600 MHz): δ 7.67 (s, 2H), 7.66 (d, J = 10.2 Hz, 1H), 7.60 (d, J = 7.2 Hz, 1H), 7.20-7.22 (m, 1H), 7.01 (t, J = 7.2 Hz, 1H), 6.98 (s, 2H); ^{13}C-NMR (DMSO-d6, 150 MHz): δ 167.8, 161.6, 137.1, 128.7, 124.8, 121.7, 120.8, 119.9, 100.8. IR (neat): 3470, 3391, 3265, 3162, 2937, 2855, 1638, 1576, 1466 cm^{-1}; HRMS (ESI): m/z calcd for C_9H_5NOS $[M-NH_3]^+$ 176.0164 Found 176.0169.

Aryl Amine Starting Materials:

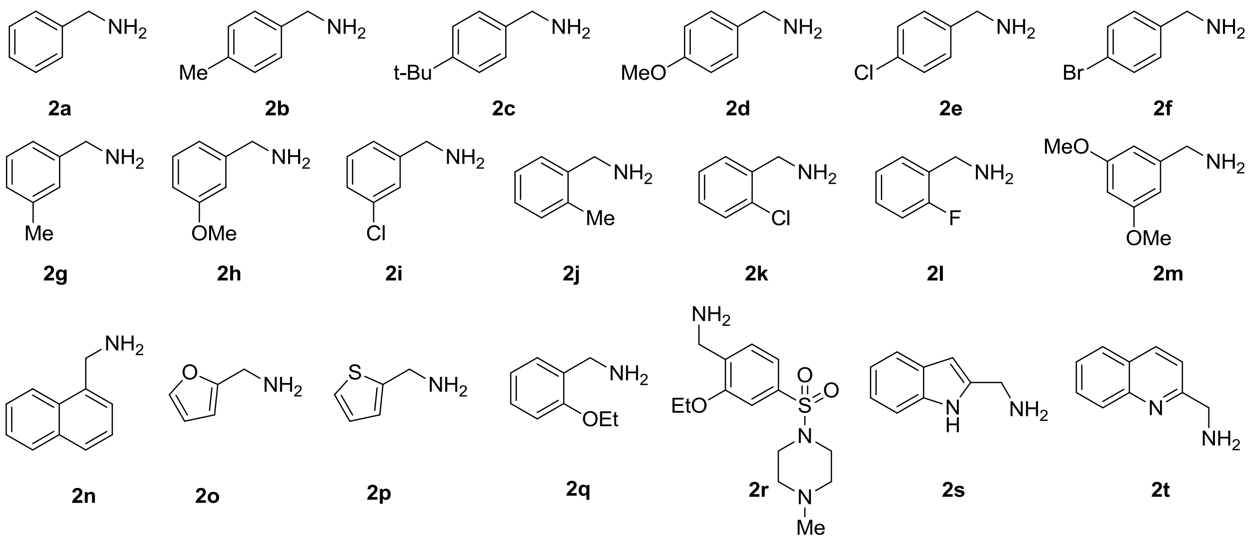

All of aryl amine substrates are commercially available except **2r**. For the synthesis of **2r**, please refer to the following

sildenafil synthesis section.

Preparation of *ortho*-Naphthoquinones Catalysts:

ortho-Naphthoquinone catalysts were prepared by the previously reported method.[9]

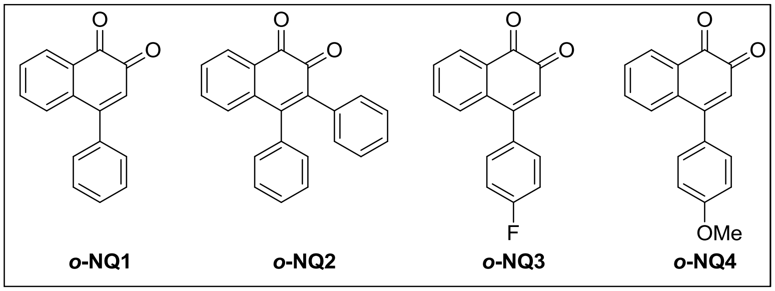

General Procedure A for the Synthesis of Fused Pyrimidin-4(3*H*)-ones:

To a dried flask charged with catalyst ***o*-NQ1** (0.01 mmol, 5 mol%), aryl benzamide (0.2 mmol), aryl benzylamine (0.24 mmol) were added 1.0 mL of DMSO followed by TFA (0.04 mmol, 20 mol%). The reaction was stirred under O_2 balloon at 100-120 °C for 12-36 h. The reaction mixture was cooled down to ambient temperature, diluted with 10 mL of water, and extracted with ethyl acetate (10 mL x 3). The combined organic phase was washed with brine, dried over Na_2SO_4, filtered and concentrated under reduced pressure. The residue was purified by column chromatography on silica gel (eluent: 30-40% acetone in hexanes or 20-40% ethyl acetate in hexanes) to give the desired products **4a-4zh** in 23-97% yields.

1.0 mmol Scale Reaction

To a dried flask charged with catalyst ***o*-NQ1** (11.7 mg, 0.05 mmol, 5 mol%), benzamide (136.2 mg, 1.0 mmol), benzylamine (131 μL, 1.2 mmol) were added 5.0 mL of DMSO followed by TFA (15 μL, 0.2 mmol, 20 mol %). The reaction was stirred under O_2 balloon at 100 °C for 36 h. The reaction mixture was cooled down to ambient temperature, diluted with 20 mL of water, and extracted with ethyl acetate (20 mL x 3). The combined organic phase was washed with brine, dried over Na_2SO_4, filtered and concentrated under reduced pressure. The residue was purified by column chromatography on silica gel (eluent: 30% Acetone in hexanes) to give the desired product **6a** in 85% yield (186 mg).

Characterization of the Products in Table 1, Scheme 2 and 3

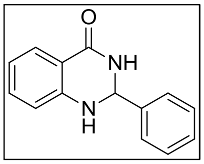

2-Phenyl-2,3-dihydroquinazolin-4(1*H*)-one (3a): The product **3a** was prepared by the General Procedure A (CH_3CN was used as solvent instead of DMSO) using **1a** (27.2 mg, 0.2 mmol) and **2a** (26 μL, 0.24 mmol). The pure product was obtained by column chromatography on silica gel using 30% acetone in hexanes as eluent. 35.8 mg (80%); white solid; The ^{1}H NMR and ^{13}C NMR spectra for this compound are consistent with previously reported literature data.[10] ^{1}H-NMR (DMSO-d$_6$, 600 MHz): δ 8.27 (s, 1H), 7.60-7.59 (m, 1H), 7.47-7.48 (m, 2H), 7.39-7.32 (m, 3H), 7.21-7.24 (m, 1H), 7.10 (s, 1H), 6.73 (d, J = 8.4 Hz, 1H), 6.64-6.67 (m, 1H), 5.74 (s, 1H); ^{13}C-NMR (DMSO-d$_6$, 150 MHz): δ 163.5, 147.8, 141.6, 133.3, 128.4, 128.3, 127.3, 126.8, 117.1, 114.9, 114.4, 66.5.

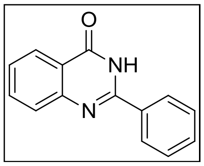

2-Phenylquinazolin 4(3*H*)-one (4a): The product **4a** was prepared by the General Procedure A using **1a** (27.2 mg, 0.2 mmol) and **2a** (26 μL, 0.24 mmol). The pure product was obtained by column chromatography on silica gel using 30% acetone in hexanes as eluent. 41 mg (93%); white solid; The ^{1}H NMR and ^{13}C NMR spectra for this compound are consistent with previously reported literature data.[11] ^{1}H-NMR (CDCl$_3$, 600 MHz): δ 11.73 (s, 1H), 8.34 (d, J = 7.8 Hz, 1H), 8.25-8.27 (m, 2H), 7.85-7.80 (m, 2H), 7.58-7.59 (m, 3H), 7.50-7.52 (m, 1H); ^{13}C-NMR (CDCl$_3$, 150 MHz): δ 164.1, 151.9, 149.7, 135.0, 133.0, 131.8, 129.2, 128.2, 127.6, 126.9, 126.5, 121.0.

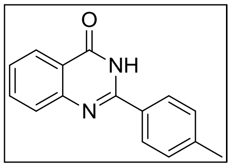

2-(*p*-Tolyl)quinazolin-4(3*H*)-one (4b): The product **4b** was prepared by the General Procedure A using **1a** (27.2 mg, 0.2 mmol) and **2b** (30 μL, 0.24 mmol). The pure product was obtained by column chromatography on silica gel using 30% ethyl acetate in hexanes as eluent. 46 mg (97%); white solid; The ^{1}H NMR and ^{13}C NMR spectra for this compound are consistent with previously reported literature data.[12] ^{1}H-NMR (DMSO-d$_6$, 600 MHz): δ 12.45 (s, 1H), 8.13 (d, J = 8.4 Hz, 1H), 8.07-8.09 (m, 2H), 7.82-7.79 (m, 1H), 7.70-7.71 (m, 1H), 7.47-7.50 (m, 1H), 7.32-7.33 (m, 2H), 2.36 (s, 3H); ^{13}C-NMR (DMSO-d$_6$, 150 MHz): δ 162.3, 152.2, 148.8, 141.4, 134.5, 129.9, 129.1, 127.7, 127.3, 126.3, 125.8, 120.9, 21.0.

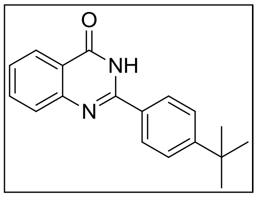

2-(4-(*tert*-Butyl)phenyl)quinazolin-4(3*H*)-one (4c): The product **4c** was prepared by the General Procedure A using **1a** (27.2 mg, 0.2 mmol) and **2c** (42 μL, 0.24 mmol). The pure product was obtained by column chromatography on silica gel using 20% acetone in hexanes as eluent. 47 mg (84%); white solid; The ^{1}H NMR and ^{13}C NMR spectra for this

compound are consistent with previously reported literature data.[14] ^{1}H-NMR ($CDCl_3$, 600 MHz): δ 11.43 (s, 1H), 8.35 (d, *J* = 7.2 Hz, 1H), 8.18-8.20 (m, 2H), 7.83 (d, *J* = 7.2 H, 1H), 7.79 (t, *J* = 7.2 Hz, 1H), 7.59-7.60 (m, 2H), 7.50 (t, *J* = 7.2 Hz, 1H), 1.40 (s, 9H); ^{13}C-NMR ($CDCl_3$, 150 MHz): δ 163.9, 155.4, 151.9, 149.9, 134.9, 130.2, 128.2, 127.3, 126.7, 126.5, 126.2, 121.1, 35.2, 31.3.

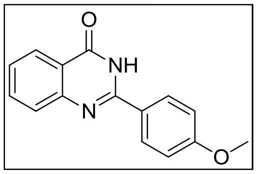

2-(4-Methoxyphenyl)quinazolin-4(3*H*)-one (4d): The product **4d** was prepared by the General Procedure A using **1a** (27.2 mg, 0.2 mmol) and **2d** (31 μL, 0.24 mmol). The pure product was obtained by column chromatography on silica gel using 30% ethyl acetate in hexanes as eluent. 46 mg (92%); white solid; The ^{1}H NMR and ^{13}C NMR spectra for this compound are consistent with previously reported literature data.[12] ^{1}H-NMR (DMSO-d_6, 600 MHz): δ 12.40 (s, 1H), 8.17-8.18 (m, 2H), 8.12 (d, *J* = 6.6 Hz, 1H), 7.78-7.80 (m, 1H), 7.68-7.69 (m, 1H), 7.45-7.47 (m, 1H), 7.06-7.08 (m, 2H), 3.83 (s, 3H); ^{13}C-NMR (DMSO-d_6, 150 MHz): δ 162.4, 161.8, 151.9, 148.9, 134.5, 129.4, 127.2, 126.1, 125.8, 124.8, 120.7, 114.0, 55.4.

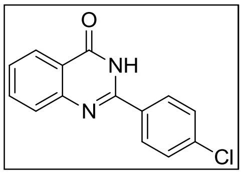

2-(4-Chlorophenyl)quinazolin-4(3*H*)-one (4e): The product **4e** was prepared by the General Procedure A using **1a** (27.2 mg, 0.2 mmol) and **2e** (30 μL, 0.24 mmol). The pure product was obtained by column chromatography on silica gel using 30% acetone in hexanes as eluent. 38 mg (75%); white solid; The ^{1}H NMR and ^{13}C NMR spectra for this compound are consistent with previously reported literature data.[12] ^{1}H-NMR (DMSO-d_6, 600 MHz): δ 12.60 (s, 1H), 8.18-8.20 (m, 2H), 8.15 (d, *J* = 7.2 Hz, 1H), 7.84 (t, *J* = 7.2 Hz, 1H), 7.73 (d, *J* = 7.2 Hz, 1H), 7.61-7.62 (m, 2H), 7.53 (t, *J* = 7.2 Hz, 1H); ^{13}C-NMR (DMSO-d_6, 150 MHz): δ 162.2, 151.4, 148.5, 136.3, 134.7, 131.5, 129.6, 128.7, 127.5, 126.8, 125.9, 121.0.

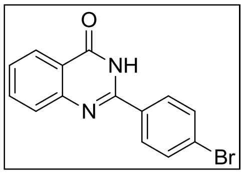

2-(4-Bromophenyl)quinazolin-4(3*H*)-one (4f): The product **4f** was prepared by the General Procedure A using **1a** (27.2 mg, 0.2 mmol) and **2f** (30 μL, 0.24 mmol). The pure product was obtained by column chromatography on silica gel using 30% acetone in hexanes as eluent. 35 mg (58%); white solid; The ^{1}H NMR and ^{13}C NMR spectra for this compound are consistent with previously reported literature data.[13] ^{1}H-NMR (DMSO-d_6, 600 MHz): δ 12.34 (s, 1H), 8.12-8.16 (m, 3H), 7.82 (t, *J* = 7.2 Hz, 1H), 7.72-7.74 (m, 3H), 7.52 (t, *J* = 7.2 Hz, 1H); ^{13}C-NMR (DMSO-d_6, 150 MHz): δ 161.7, 151.2, 148.2, 134.1, 131.7, 131.1, 129.4, 127.0, 126.2, 125.4, 124.7, 120.7.

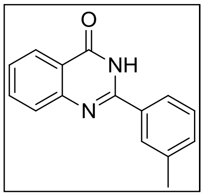

2-(*m*-Tolyl)quinazolin-4(3*H*)-one (4g): The product **4g** was prepared by the General Procedure A using **1a** (27.2 mg, 0.2 mmol) and **2g** (30 μL, 0.24 mmol). The pure product was obtained by column chromatography on silica gel using 20% acetone in hexanes as eluent. 42 mg (90%) white solid; The ^{1}H NMR and ^{13}C NMR spectra for this compound are consistent with previously reported literature data.[13] ^{1}H-NMR (DMSO-d$_6$, 600 MHz): δ 12.46 (s, 1H), 8.13-8.14 (m, 1H), 8.01 (s, 1H), 7.96 (d, *J* = 7.2 Hz, 1H), 7.80-7.82 (m, 1H), 7.72-7.73 (m, 1H), 7.48-7.51 (m, 1H), 7.43-7.37 (m, 2H), 2.39 (s, 3H); ^{13}C-NMR (DMSO-d$_6$, 150 MHz): δ 162.2, 152.3, 148.8, 137.9, 134.5, 132.6, 132.0, 128.5, 128.3, 127.5, 126.5, 125.8, 124.9, 121.0, 20.9.

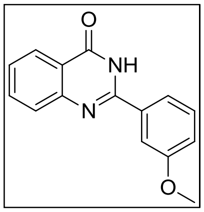

2-(3-Methoxyphenyl)quinazolin-4(3*H*)-one (4h): The product **4h** was prepared by the General Procedure A using **1a** (27.2 mg, 0.2 mmol) and **2h** (31 μL, 0.24 mmol). The pure product was obtained by column chromatography on silica gel using 30% acetone in hexanes as eluent. 43 mg (83%); white solid; The ^{1}H NMR and ^{13}C NMR spectra for this compound are consistent with previously reported literature data.[10] ^{1}H-NMR (DMSO-d$_6$, 600 MHz): δ 12.52 (s, 1H), 8.14 (d, *J* = 7.2 Hz, 1H), 7.78-7.82 (m, 2H), 7.72-7.74 (m, 2H), 7.48-7.51 (m, 1H), 7.42-7.44 (m, 1H), 7.18-7.13 (m, 1H), 3.85 (s, 3H); ^{13}C-NMR (DMSO-d$_6$, 150 MHz): δ 162.5, 159.3, 152.2, 148.7, 134.5, 134.2, 129.7, 127.4, 126.5, 125.8, 121.0, 120.1, 117.5, 112.5, 55.4.

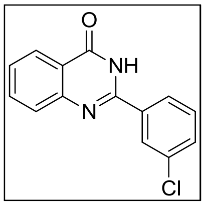

2-(3-Chlorophenyl)quinazolin-4(3*H*)-one (4i): The product **4i** was prepared by the General Procedure A using **1a** (27.2 mg, 0.2 mmol) and **2i** (29 μL, 0.3 mmol). The pure product was obtained by column chromatography on silica gel using 30% acetone in hexanes as eluent. 49 mg (91%); white solid; The ^{1}H NMR and ^{13}C NMR spectra for this compound are consistent with previously reported literature data.[13] ^{1}H-NMR (DMSO-d$_6$, 600 MHz): δ 12.36 (s, 1H), 8.24 (s, 1H), 8.14-8.16 (m, 2H), 7.81-7.84 (m, 1H), 7.75 (d, *J* = 8.4 Hz, 1H), 7.62 (d, *J* = 8.4 Hz, 1H), 7.51-7.57 (m, 2H); ^{13}C-NMR (DMSO-d$_6$, 150 MHz): δ 161.7, 150.8, 148.1, 134.6, 134.0, 133.1, 130.6, 129.9, 127.1, 127.0, 126.3, 126.0, 125.4, 120.8.

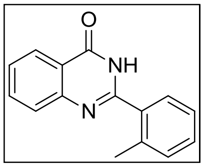

2-(*o*-Tolyl)quinazolin-4(3*H*)-one (4j): The product **4j** was prepared by the General Procedure A using **1a** (27.2 mg, 0.2 mmol) and **2j** (30 μL, 0.24 mmol). The pure product was obtained by column chromatography on silica gel using 20% acetone in hexanes as eluent. 38 mg (81%); white solid; The ^{1}H NMR and ^{13}C NMR spectra for this compound are consistent with previously reported literature

data.[13] ^{1}H-NMR (DMSO-d_6, 600 MHz): δ 12.44 (s, 1H), 8.16 (d, J = 8.4 Hz, 1H), 7.83-7.81 (m, 1H), 7.67-7.68 (m, 1H), 7.54-7.49 (m, 2H), 7.40-7.43 (m, 1H), 7.30-7.34 (m, 2H), 2.38 (s, 3H); ^{13}C-NMR (DMSO-d_6, 150 MHz): δ 161.8, 154.3, 148.7, 136.1, 134.4, 134.2, 130.5, 129.9, 129.1, 127.3, 126.6, 125.8, 125.7, 121.0, 19.5.

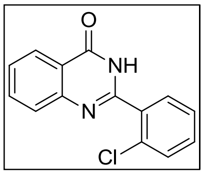

2-(2-Chlorophenyl)quinazolin-4(3*H*)-one (4k): The product **4k** was prepared by the General Procedure A using **1a** (27.2 mg, 0.2 mmol) and **2k** (29 μL, 0.24 mmol). The pure product was obtained by column chromatography on silica gel using 20% acetone in hexanes as eluent. 40 mg (79%); white solid; The ^{1}H NMR and ^{13}C NMR spectra for this compound are consistent with previously reported literature data.[12] ^{1}H-NMR ($CDCl_3$, 600 MHz): δ 10.24 (s, 1H), 8.29 (d, J = 8.4 Hz, 1H), 7.84-7.79 (m, 3H), 7.51-7.54 (m, 2H), 7.47-7.49 (m, 1H), 7.43-7.46 (m, 1H); ^{13}C-NMR ($CDCl_3$, 150 MHz): δ 162.1, 151.1, 149.1, 135.0, 132.8, 132.2, 132.0, 131.6, 130.8, 128.2, 127.7, 127.6, 126.7, 121.3.

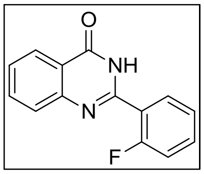

2-(2-Fluorophenyl)quinazolin-4(3*H*)-one (4l): The product **4l** was prepared by the General Procedure A using **1a** (27.2 mg, 0.2 mmol) and **2l** (27 μL, 0.24 mmol). The pure product was obtained by column chromatography on silica gel using 20% acetone in hexanes as eluent. 35 mg (83%); white solid; The ^{1}H NMR and ^{13}C NMR spectra for this compound are consistent with previously reported literature data.[15] ^{1}H-NMR ($CDCl_3$, 600 MHz): δ 10.07 (s, 1H), 8.36-8.33 (m, 1H), 8.30 (d, J = 7.2 Hz, 1H), 7.80-7.81 (m, 2H), 7.56-7.50 (m, 2H), 7.34-7.36 (m, 1H), 7.23 (dd, J = 12.4, 8.3 Hz, 1H); ^{13}C-NMR ($CDCl_3$, 150 MHz): δ 162.0, 160.9 (d, J = 248.6 Hz), 149.1, 148.4, 134.9, 133.7 (d, J = 8.7 Hz), 131.5, 128.2, 127.4, 126.7, 125.4, 121.4, 120.2 (d, J = 8.6 Hz), 116.8 (d, J = 23.1 Hz).

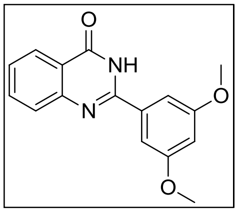

2-(3,5-Dimethoxyphenyl)quinazolin-4(3*H*)-one (4m): The product **4m** was prepared by the General Procedure A using **1a** (27.2 mg, 0.2 mmol) and **2m** (40 mg, 0.24 mmol). The pure product was obtained by column chromatography on silica gel using 30% acetone in hexanes as eluent. 34 mg (60%); white solid; The ^{1}H NMR and ^{13}C NMR spectra for this compound are consistent with previously reported literature data.[13] ^{1}H-NMR (DMSO-d_6, 600 MHz): δ 12.49 (s, 1H), 8.13-8.14 (m, 1H), 7.83-7.80 (m, 1H), 7.72-7.74 (m, 1H), 7.49-7.52 (m, 1H), 7.37-7.38 (m, 2H), 6.67-6.68 (m, 1H), 3.83 (s, 6H); ^{13}C-NMR (DMSO-d_6, 150 MHz): δ 162.2, 160.5, 151.9, 148.5, 134.5, 127.5, 126.6, 125.8, 121.0, 105.5 (2C), 103.8, 55.5.

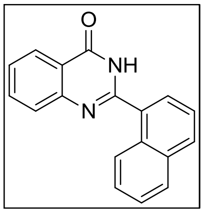

2-(Naphthalen-1-yl)quinazolin-4(3*H*)-one (4n): The product **4n** was prepared by the General Procedure A using **1a** (27.2 mg, 0.2 mmol) and **2n** (36 μL, 0.24 mmol). The pure product was obtained by column chromatography on silica gel using 20% acetone in hexanes as eluent. 37 mg (66%); white solid; The ^{1}H NMR and ^{13}C NMR spectra for this compound are consistent with previously reported literature data.[12] ^{1}H-NMR (DMSO-d$_6$, 600 MHz): δ 12.67 (s, 1H), 8.21 (d, *J* = 7.2 Hz, 1H), 8.16 (d, *J* = 7.2 Hz, 1H), 8.10-8.12 (m, 1H), 8.03-8.04 (m, 1H), 7.84-7.87 (m, 1H), 7.78-7.79 (m, 1H), 7.72-7.73 (m, 1H), 7.62-7.65 (m, 1H), 7.61-7.56 (m, 3H); ^{13}C-NMR (DMSO-d$_6$, 150 MHz): δ 161.9, 153.7, 148.7, 134.5, 133.1, 131.7, 130.4, 130.2, 128.3, 127.7, 127.5, 127.1, 126.8, 126.4, 125.8, 125.2, 125.1, 121.2.

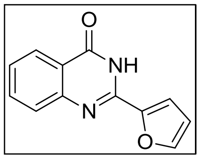

2-(Furan-2-yl)quinazolin-4(3*H*)-one (4o): The product **4o** was prepared by the General Procedure A using **1a** (27.2 mg, 0.2 mmol) and **2o** (23 μL, 0.24 mmol). The pure product was obtained by column chromatography on silica gel using 30% acetone in hexanes as eluent. 23 mg (54%); white solid; The ^{1}H NMR and ^{13}C NMR spectra for this compound are consistent with previously reported literature data.[13] ^{1}H-NMR (DMSO-d$_6$, 600 MHz): δ 12.50 (s, 1H), 8.10-8.11 (m, 1H), 7.99 (s, 1H), 7.81-7.78 (m, 1H), 7.67-7.68 (m, 1H), 7.61-7.62 (m, 1H), 7.48 (t, *J* = 7.2 Hz, 1H), 6.73-6.74 (m, 1H); ^{13}C-NMR (DMSO-d$_6$, 150 MHz): δ 161.2, 148.5, 146.1, 146.0, 143.8, 134.2, 127.0, 126.1, 125.6, 121.0, 114.1, 112.2.

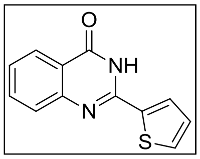

2-(Thiophen-2-yl)quinazolin-4(3*H*)-one (4p): The product **4p** was prepared by the General Procedure A using **1a** (27.2 mg, 0.2 mmol) and **2p** (25 μL, 0.24 mmol). The pure product was obtained by column chromatography on silica gel using 20% acetone in hexanes as eluent. 35 mg (75%); white solid; The ^{1}H NMR and ^{13}C NMR spectra for this compound are consistent with previously reported literature data.[13] ^{1}H-NMR (DMSO-d$_6$, 600 MHz): δ 12.65 (s, 1H), 8.22 (m, 1H), 8.12-8.11 (m, 1H), 7.85-7.86 (m, 1H), 7.81-7.78 (m, 1H), 7.63-7.64 (m, 1H), 7.46-7.48 (m, 1H), 7.21-7.23 (m, 1H); ^{13}C-NMR (DMSO-d$_6$, 150 MHz): δ 161.8, 148.6, 147.8, 137.3, 134.7, 132.1, 129.4, 128.5, 126.9, 126.3, 126.0, 120.8.

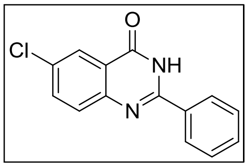

6-Chloro-2-phenylquinazolin-4(3*H*)-one (4q): The product **4q** was prepared by the General Procedure A using **1b** (34.1 mg, 0.2 mmol) and **2a** (26 μL, 0.24 mmol). The pure product was obtained by column chromatography on silica gel using 30% acetone in hexanes as eluent. 43 mg (84%); white solid; The ^{1}H NMR and ^{13}C NMR spectra for this compound are consistent with previously reported literature data.[14] ^{1}H-NMR (DMSO-d$_6$, 600 MHz): δ 12.70 (s, 1H), 8.15-8.16 (m, 2H), 8.06-8.07

(m, 1H), 7.83-7.85 (m, 1H), 7.74-7.75 (m, 1H), 7.57-7.60 (m, 1H), 7.52-7.55 (m, 2H); ^{13}C-NMR (DMSO-d_6, 150 MHz): δ 161.3, 152.9, 147.4, 134.7, 132.5, 131.6, 130.7, 129.7, 128.6, 127.8, 124.9, 122.2.

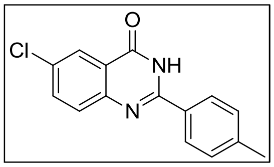

6-Chloro-2-(*p*-tolyl)quinazolin-4(3*H*)-one (4r): The product **4r** was prepared by the General Procedure A using **1b** (34.1 mg, 0.2 mmol) and **2b** (30 μL, 0.24 mmol). The pure product was obtained by column chromatography on silica gel using 30% acetone in hexanes as eluent. 45 mg (83%); white solid; The ^{1}H NMR and ^{13}C NMR spectra for this compound are consistent with previously reported literature data.[12] ^{1}H-NMR (DMSO-d_6, 600 MHz): δ 12.25 (s, 1H), 8.06-8.09 (m, 3H), 7.79-7.81 (m, 1H), 7.71-7.73 (m, 1H), 7.34-7.35 (m, 2H), 2.40 (s, 3H); ^{13}C-NMR (DMSO-d_6, 150 MHz): δ 160.9, 152.5, 147.2, 141.2, 134.1, 130.2, 129.4, 129.1, 128.7, 127.4, 124.5, 121.8, 20.5.

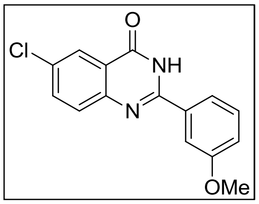

6-Chloro-2-(3-methoxyphenyl)quinazolin-4(3*H*)-one (4s): The product **4s** was prepared by the General Procedure A using **1b** (34.1 mg, 0.2 mmol) and **2h** (30 μL, 0.24 mmol). The pure product was obtained by column chromatography on silica gel using 20% acetone in hexanes as eluent. 43 mg (74%); white solid, m.p. 278-279 °C; ^{1}H-NMR (DMSO-d_6, 600 MHz): δ 12.38 (s, 1H), 8.08 (s, 1H), 7.82-7.75 (m, 4H), 7.43-7.45 (m, 1H), 7.14-7.15 (m, 1H), 3.87 (s, 3H); ^{13}C-NMR (DMSO-d_6, 150 MHz): δ 160.7, 159.1, 152.3, 146.9, 134.0, 133.5, 130.3, 129.1(2), 129.0(5), 124.4, 121.9, 119.8, 117.3, 112.6, 55.1; IR(neat): 3175, 3034, 2922, 2844, 1677, 1580, 1463, 1287, 1237 cm^{-1}; HRMS (ESI): m/z calcd for $C_{15}H_{12}ClN_2O_2$ $[M+H]^+$ 287.0581 Found 287.0591.

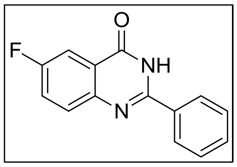

6-Fluoro-2-phenylquinazolin-4(3*H*)-one (4t): The product **4t** was prepared by the General Procedure A using **1c** (30.8 mg, 0.2 mmol) and **2a** (26 μL, 0.24 mmol). The pure product was obtained by column chromatography on silica gel using 30% acetone in hexanes as eluent. 32 mg (67%); white solid; The ^{1}H NMR and ^{13}C NMR spectra for this compound are consistent with previously reported literature data.[10] ^{1}H-NMR (DMSO-d_6, 600 MHz): δ 12.60 (s, 1H), 8.15-8.16 (m, 2H), 7.82-7.79 (m, 2H), 7.68-7.72 (m, 1H), 7.52-7.59 (m, 3H); ^{13}C-NMR (DMSO-d_6, 151 MHz): δ 161.7, 159.9 (d, J = 243.0 Hz), 151.9, 145.6, 132.6, 131.4, 130.2 (d, J = 4.4 Hz), 128.6, 127.7, 123.0 (d, J = 23.1 Hz), 122.1 (d, J = 8.7 Hz), 110.5 (d, J = 23.0 Hz).

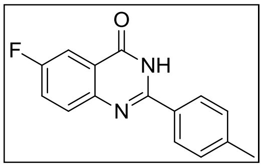

6-Fluoro-2-(*p*-tolyl)quinazolin-4(3*H*)-one (4u): The product **4u** was prepared by the General Procedure A using **1c** (30.8 mg, 0.2 mmol) and **2b** (30 μL, 0.24 mmol). The pure product was obtained by column chromatography on silica gel using 30% acetone in

hexanes as eluent. 32 mg (63%); white solid, m.p. 275-277 ºC; ^{1}H-NMR (DMSO-d$_6$, 600 MHz): δ 12.58 (s, 1H), 8.05-8.07 (m, 2H), 7.81-7.77 (m, 2H), 7.71-7.68 (m, 1H), 7.32-7.34 (m, 2H), 2.37 (s, 3H); ^{13}C-NMR (DMSO-d$_6$, 150 MHz): δ 162.3, 160.4 (d, *J* = 244.1), 152.3, 146.2, 142.0, 130.7 (d, *J* = 5.7 Hz), 130.3, 129.7, 128.2, 123.5 (d, *J* = 24.5 Hz), 122.6 (d, *J* = 7.2 Hz), 111.0 (d, *J* = 23.1 Hz), 21.5; IR(neat): 3186, 3030, 2937, 1653, 1343, 1280 cm^{-1}; HRMS (ESI): m/z calcd for $C_{15}H_{12}FN_2O$ [M+H]$^+$ 255.0928 Found 255.0930.

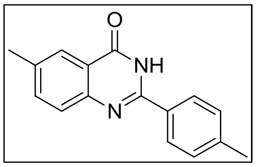

6-Methyl-2-(*p*-tolyl)quinazolin-4(3*H*)-one (4v): The product **4v** was prepared by the General Procedure A using **1d** (30.0 mg, 0.2 mmol) and **2b** (30 μL, 0.24 mmol). The pure product was obtained by column chromatography on silica gel using 20% acetone in hexanes as eluent. 40 mg (80%); white solid; The ^{1}H NMR and ^{13}C NMR spectra for this compound are consistent with previously reported literature data.[16] ^{1}H-NMR (DMSO-d$_6$, 600 MHz): δ 12.15 (s, 1H), 8.08 (d, *J* = 8.4 Hz, 2H), 7.94 (s, 1H), 7.60-7.63 (m, 2H), 7.33 (d, *J* = 8.4 Hz, 2H), 2.45 (s, 3H), 2.39 (s, 3H); ^{13}C-NMR (DMSO-d$_6$, 150 MHz): δ 161.8, 151.2, 146.5, 140.8, 135.6, 135.3, 129.8, 128.7, 127.2, 126.8, 124.9, 120.4, 20.5, 20.4.

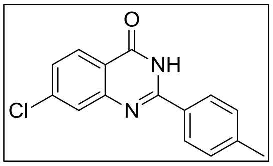

7-Chloro-2-(*p*-tolyl)quinazolin-4(3*H*)-one (4w): The product **4w** was prepared by the General Procedure A using **1e** (30.8 mg, 0.2 mmol) and **2b** (30 μL, 0.24 mmol). The pure product was obtained by column chromatography on silica gel using 30% acetone in hexanes as eluent. 33 mg (61%); white solid, m.p. 313-314 ºC; ^{1}H-NMR (DMSO-d$_6$, 600 MHz): δ 12.23 (s, 1H), 8.08-8.13 (m, 3H), 7.72 (s, 1H), 7.47-7.48 (m, 1H), 7.34-7.35 (m, 2H), 2.40 (s, 3H); ^{13}C-NMR (DMSO-d$_6$, 150 MHz): δ 161.1, 153.4, 149.5, 141.3, 138.6, 129.3, 128.6, 127.4, 127.3, 125.9, 125.8, 119.3, 20.4; IR(neat): 3190, 2918, 2864, 1673, 1556, 1479 cm^{-1}; HRMS (ESI): m/z calcd for $C_{15}H_{12}ClN_2O$ [M+H]$^+$ 271.0632 Found 271.0639.

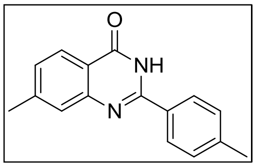

7-Methyl-2-(*p*-tolyl)quinazolin-4(3*H*)-one (4x): The product **4x** was prepared by the General Procedure A using **1f** (30.0 mg, 0.2 mmol) and **2b** (30 μL, 0.24 mmol). The pure product was obtained by column chromatography on silica gel using 30% acetone in hexanes as eluent. 38 mg (76%); white solid; The ^{1}H NMR and ^{13}C NMR spectra for this compound are consistent with previously reported literature data.[17] ^{1}H-NMR (DMSO-d$_6$, 600 MHz): δ 12.18 (s, 1H), 8.07-8.08 (m, 2H), 8.02 (d, *J* = 7.8 Hz, 1H), 7.51 (s, 1H), 7.29-7.33 (m, 3H), 2.46 (s, 3H), 2.38 (s, 3H); ^{13}C-NMR (DMSO-d$_6$, 150 MHz): δ 161.8, 152.0, 148.7, 144.6, 141.0, 129.8, 128.8, 127.4, 127.3, 126.8, 125.4, 118.3, 21.0, 20.6.

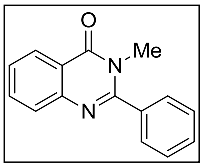

3-Methyl-2-phenylquinazolin-4(3*H*)-one (4y): The product **4y** was prepared by the General Procedure A using **1g** (30.0 mg, 0.2 mmol) and **2a** (26 μL, 0.24 mmol). The pure product was obtained by column chromatography on silica gel using 30% ethyl acetate in hexanes as eluent. 41 mg (88%); white solid; The ^{1}H NMR and ^{13}C NMR spectra for this compound are consistent with previously reported literature data.[18] ^{1}H-NMR ($CDCl_3$, 600 MHz): δ 8.33 (d, *J* = 8.4 Hz, 1H), 7.73-7.75 (m, 2H), 7.57-7.49 (m, 6H), 3.50 (s, 3H); ^{13}C-NMR ($CDCl_3$, 150 MHz): δ 162.9, 156.3, 147.5, 135.5, 134.4, 130.2, 129.0, 128.1, 127.6, 127.1, 126.8, 120.7, 34.4.

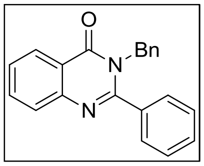

3-Benzyl-2-phenylquinazolin-4(3*H*)-one (4z): The product **4z** was prepared by the General Procedure A using **1h** (45.2 mg, 0.2 mmol) and **2a** (26 μL, 0.24 mmol). The pure product was obtained by column chromatography on silica gel using 20% ethyl acetate in hexanes as eluent. 58 mg (93%); white solid; The ^{1}H NMR and ^{13}C NMR spectra for this compound are consistent with previously reported literature data.[19] ^{1}H-NMR ($CDCl_3$, 600 MHz): δ 8.38 (d, *J* = 8.4 Hz, 1H), 7.80-7.76 (m, 2H), 7.54-7.52 (m, 1H), 7.45-7.47 (m, 1H), 7.38-7.41 (m, 2H), 7.32-7.35 (m, 2H), 7.19-7.20 (m, 3H), 6.92-6.93 (m, 2H), 5.28 (s, 2H); ^{13}C-NMR ($CDCl_3$, 150 MHz): δ 162.6, 156.5, 147.4, 136.7, 135.4, 134.7, 130.0, 128.7, 128.6, 128.1, 127.7, 127.5, 127.3, 127.2, 127.1, 121.0, 48.9.

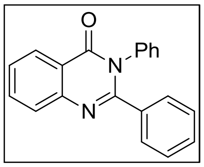

2,3-Diphenylquinazolin-4(3*H*)-one (4za): The product **4za** was prepared by the General Procedure A using **1i** (42.4 mg, 0.2 mmol) and **2a** (26 μL, 0.24 mmol). The pure product was obtained by column chromatography on silica gel using 20% ethyl acetate in hexanes as eluent. 57 mg (95%); white solid; The ^{1}H NMR and ^{13}C NMR spectra for this compound are consistent with previously reported literature data.[18] ^{1}H-NMR ($CDCl_3$, 600 MHz): δ 8.36 (d, *J* = 7.6 Hz, 1H), 7.84-7.80 (m, 2H), 7.55-7.53 (m, 1H), 7.14-7.34 (m, 10H); ^{13}C-NMR ($CDCl_3$,150 MHz): δ 162.4, 155.3, 147.6, 137.8, 135.6, 134.9, 129.4, 129.2, 129.1(2C), 128.5, 128.1, 127.9, 127.4, 127.3, 121.1.

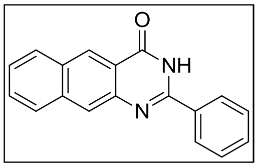

2-Phenylbenzo[*g*]quinazolin-4(3*H*)-one (4zb): The product **4zb** was prepared by the General Procedure A using **1j** (37 mg, 0.2 mmol) and **2a** (26 μL, 0.24 mmol). The pure product was obtained by column chromatography on silica gel using 20% acetone in hexanes as eluent. 25 mg (46%); white solid; The ^{1}H NMR and ^{13}C NMR spectra for this compound are consistent with previously reported literature data.[1] ^{1}H-NMR (DMSO-d_6, 600 MHz): δ 12.20 (s, 1H), 8.85 (s, 1H), 8.30 (s, 1H),

8.22-8.24 (m, 2H), 8.19 (d, $J = 8.4$ Hz, 1H), 8.10 (d, $J = 8.4$ Hz, 1H), 7.66 (t, $J = 7.2$ Hz, 1H), 7.60-7.55 (m, 4H); ^{13}C-NMR (DMSO-d$_6$, 150 MHz): δ 162.4, 151.1, 143.9, 136.2, 132.8, 131.0, 130.7, 129.0, 128.3, 128.2, 127.5, 127.4, 127.0, 125.9, 124.6, 119.9.

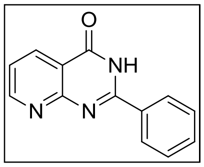

2-Phenylpyrido[2,3-d]pyrimidin-4(3*H*)-one (4zc): The product **4zc** was prepared by the General Procedure A using **1k** (27.4 mg, 0.2 mmol) and **2a** (26 μL, 0.24 mmol). The pure product was obtained by column chromatography on silica gel using 30% acetone in hexanes as eluent. 41 mg (92%); white solid; The ^{1}H NMR and ^{13}C NMR spectra for this compound are consistent with previously reported literature data.[20] ^{1}H-NMR (DMSO-d$_6$, 600 MHz): δ 12.82 (s, 1H), 8.95-8.96 (m, 1H), 8.52-8.51 (m, 1H), 8.20-8.21 (m, 2H), 7.60-7.63 (m, 1H), 7.55-7.57 (m, 2H), 7.51-7.53 (m, 1H), ^{13}C-NMR (DMSO-d$_6$, 150 MHz): δ 163.0, 158.7, 156.1, 155.4, 135.5, 132.4, 131.9, 128.7, 128.1, 122.2, 116.2.

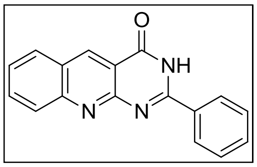

2-Phenylpyrimido[4,5-*b*]quinolin-4(3*H*)-one (4zd): The product **4zd** was prepared by the General Procedure A using **1l** (37.4 mg, 0.2 mmol) and **2a** (26 μL, 0.24 mmol). The pure product was obtained by column chromatography on silica gel using 3% MeOH in DCM as eluent. 13 mg (23%); Yellow solid; The ^{1}H NMR and ^{13}C NMR spectra for this compound are consistent with previously reported literature data.[2] ^{1}H-NMR (DMSO-d$_6$, 600 MHz): δ 12.40 (s, 1H), 9.25 (s, 1H), 8.29-8.30 (m, 2H), 8.22-8.24 (m, 1H), 8.07-8.09 (m, 1H), 7.91-7.93 (m, 1H), 7.58-7.65 (m, 4H); ^{13}C-NMR (DMSO-d$_6$, 150 MHz): δ 163.2, 155.9, 155.8, 150.8, 137.6, 132.6, 132.2, 131.5, 129.0, 128.2, 128.0, 127.8, 125.9(2C), 115.2.

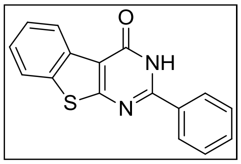

2-Phenylbenzo[4,5]thieno[2,3-*d*]pyrimidin-4(3*H*)-one (4ze) : The product **4zf** was prepared by the General Procedure A using **1m** (38.4 mg, 0.2 mmol) and **2a** (26 μL, 0.24 mmol). The pure product was obtained by column chromatography on silica gel using 20 % acetone in hexanes as eluent. 37.8 mg (68%); white solid, m.p. 322-323 ^{o}C; ^{1}H-NMR (DMSO-d$_6$, 600 MHz): δ 12.62 (s, 1H), 8.53 (d, $J = 7.8$ Hz, 1H), 8.22 (d, $J = 8.4$ Hz, 2H), 8.02 (d, $J = 7.8$ Hz, 1H), 7.63-7.48 (m, 5H); ^{13}C-NMR (DMSO-d$_6$, 150 MHz): δ 166.0, 157.9, 154.7, 134.6, 133.2, 131.5, 131.2, 128.1, 127.5, 125.5, 125.1, 123.4, 122.1, 115.4; IR(neat): 3086, 2972, 2933, 2864, 1662, 1533, 1448 cm^{-1}; HRMS (ESI): m/z calcd for $C_{16}H_{11}N_2OS$ [M]$^+$ 279.0586 Found 279.0594.

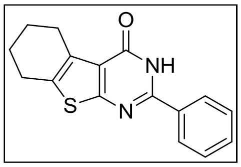

2-Phenyl-5,6,7,8-tetrahydrobenzo[4,5]thieno[2,3-*d*]pyrimidin-4(3*H*)-one (4zf): The product **4zf** was prepared by the General Procedure A using **1n** (39.2 mg, 0.2 mmol) and **2a** (26 μL, 0.24 mmol). The pure product was obtained by column chromatography on silica gel using 20% acetone in hexanes as eluent. 24.2 mg (43%); white solid, m.p. 294-295 °C; ^{1}H-NMR (DMSO-d$_6$, 600 MHz): δ 12.22 (s, 1H), 8.10-8.12 (m, 2H), 7.57-7.49 (m, 3H), 2.92-2.93 (m, 2H), 2.75-2.77 (m, 2H), 1.86-1.77 (m, 4H); ^{13}C-NMR (DMSO-d$_6$, 150 MHz): δ 162.7, 158.3, 151.8, 132.1, 131.8, 130.7, 130.5, 128.1, 127.2, 120.6, 24.9, 24.2, 22.2, 21.4; IR(neat): 3076, 2933, 2858, 1653, 1533, 1483, 1284 cm^{-1}; HRMS (ESI): m/z calcd for $C_{16}H_{15}N_2OS$ $[M+H]^+$ 283.0899 Found 283.0906.

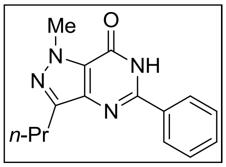

1-Methyl-5-phenyl-3-propyl-*1H*-pyrazolo[4,3-*d*]pyrimidin-7(6*H*)-one (4zg): The product **4zg** was prepared by the General Procedure A using **1o** (36.8 mg, 0.2 mmol) and **2a** (26 μL, 0.24 mmol). The pure product was obtained by column chromatography on silica gel using 30% acetone in hexanes as eluent. 37 mg (70%); white solid; The ^{1}H NMR and ^{13}C NMR spectra for this compound are consistent with previously reported literature data.[14] ^{1}H-NMR (DMSO-d$_6$, 600 MHz): δ 12.13 (s, 1H), 8.06-8.07 (m, 2H), 7.53-7.49 (m, 3H), 4.15 (s, 3H), 2.82 (t, J = 7.2 Hz, 2H), 1.76-1.82 (m, 2H), 0.97 (t, J = 7.2 Hz, 3H); ^{13}C-NMR (DMSO-d$_6$, 150 MHz): δ 154.2, 149.8, 144.8, 137.7, 132.7, 130.1, 128.0, 127.1, 124.2, 37.4, 26.8, 21.1, 13.3.

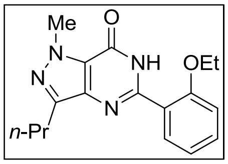

5-(2-ethoxyphenyl)-1-methyl-3-propyl-1*H*-pyrazolo[4,3-d]pyrimidin-7(6*H*)-one (4zh): The product **4zh** was prepared by the General Procedure A using **1o** (36.8 mg, 0.2 mmol) and **2q** (36 mg, 0.24 mmol). The pure product was obtained by column chromatography on silica gel using 20% acetone in hexanes as eluent. 40 mg (80%); white solid; The ^{1}H NMR and ^{13}C NMR spectra for this compound are consistent with previously reported literature data.[21] ^{1}H-NMR (CDCl$_3$, 600 MHz): δ 11.11 (s, 1H), 8.44-8.46 (m, 1H), 7.45-7.42 (m, 1H), 7.12 (t, J = 8.4 Hz, 1H), 7.02 (d, J = 8.4 Hz, 1H), 4.25-4.28 (m, 5H), 2.92 (t, J = 7.8 Hz, 2H), 1.83-1.89 (m, 2H), 1.59 (t, J = 7.2 Hz, 3H), 1.02 (t, J = 7.2 Hz, 3H); ^{13}C-NMR (CDCl$_3$, 150 MHz): δ 156.6, 154.0, 148.5, 146.7, 138.8, 132.5, 131.2, 124.6, 121.9, 120.3, 113.0, 65.4, 38.3, 27.9, 22.5, 14.8, 14.2.

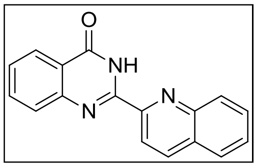

2-(Quinolin-2-yl)quinazolin-4(3*H*)-one (**6**): The product **6** was prepared by the General Procedure A (140 mol% of TFA, 120 °C, 24 h) using **1a** (27.2 mg, 0.2 mmol) and **2t** (36 mg, 0.24 mmol). The pure product was obtained by column chromatography on silica gel using 20% ethyl acetate in hexanes as eluent. 34 mg (60 %); White solid; The ^{1}H NMR and ^{13}C NMR spectra for this

compound are consistent with previously reported literature data.[22] ^{1}H-NMR ($CDCl_3$, 600 MHz): δ 11.17 (s, 1H), 8.61 (d, *J* = 9.0 Hz, 1H), 8.36 (dd, *J* = 7.8, 1.2 Hz, 1H), 8.32 (d, *J* = 9.0 Hz, 1H), 8.12 (d, *J* = 9.0 Hz, 1H), 7.86 (t, *J* = 9.0 Hz, 2H), 7.80-7.76 (m, 2H), 7.60-7.62 (m, 1H), 7.53-7.51 (m, 1H); ^{13}C-NMR ($CDCl_3$, 150 MHz): δ 161.6, 149.3, 149.1, 148.2, 146.9, 137.8, 134.7, 130.6, 129.8, 129.4, 128.4, 128.4, 127.9, 127.7, 126.9, 122.8, 118.6.

Synthesis of Bouchardatine in Scheme 5

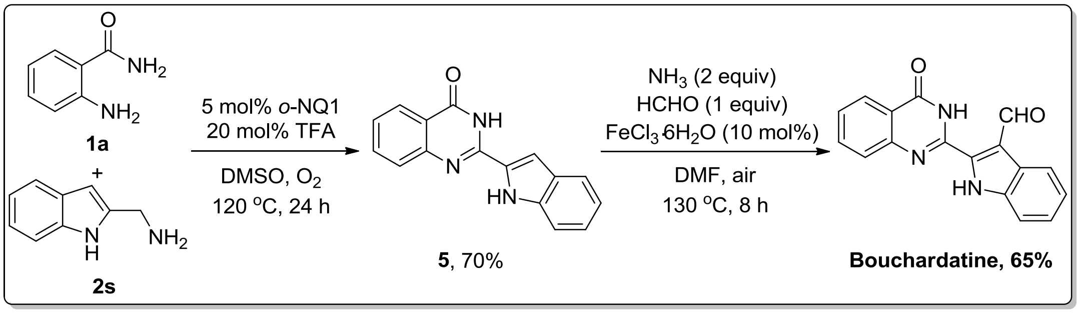

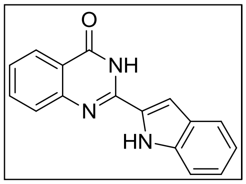

2-(1*H*-Indol-2-yl)quinazolin-4(3*H*)-one (5) : The intermediate compound **8** was prepared by the General Procedure A (120 °C, 24 h) using **1a** (54 mg, 0.4 mmol) and **2s** (29 mg, 0.2 mmol). The residue was purified by column chromatography on silica gel (eluent: 20% ethyl acetate in hexanes) to give the desired products **5** (35 mg, 70% yield). Yellow solid; The ^{1}H NMR and ^{13}C NMR spectra for this compound are consistent with previously reported literature data.[23] ^{1}H-NMR (DMSO-d$_6$, 600 MHz): δ 12.60 (s, 1H), 11.79 (s, 1H), 8.15 (d, *J* = 7.2 Hz, 1H), 7.85-7.83 (m, 1H), 7.72-7.74 (m, 1H), 7.66 (s, 1H), 7.63 (d, *J* = 8.4 Hz, 1H), 7.53-7.49 (m, 2H), 7.20-7.23 (m, 1H), 7.05 (t, *J* = 7.2 Hz, 1H); ^{13}C-NMR (DMSO-d$_6$, 150 MHz): δ 161.8, 148.7, 146.6, 137.7, 134.7, 130.0, 127.4, 126.9, 126.3, 126.1, 124.1, 121.5, 121.2, 120.0, 112.4, 105.0.

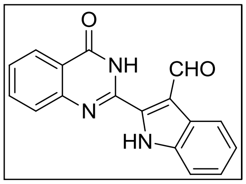

Bouchardatine (2-(4-Oxo-3,4-dihydroquinazolin-2-yl)-1*H*-indole-3-carbaldehyde): To a flask charged with a mixture of **5** (0.2 mmol, 52 mg) and $FeCl_3·6H_2O$ (10 mol%) in DMF were added 37% HCHO solution in water (0.2 mmol, 16 μL) and 28-30% NH_3 solution in water (0.4 mmol, 27 μL). The reaction was stirred at 130 °C under air for 8 h. The reaction mixture was cooled down to ambient temperature and diluted with 5 mL of brine. The 0.5 mL of 0.5 M HCl was added to the mixture. The reaction mixture was further stirred for 30 min and extracted with ethyl acetate (10 mL x 5), washed with saturated

$NaHCO_3$ (10 mL x 2), dried over Na_2SO_4, filtered, and concentrated under reduced pressure. The residue was purified by column chromatography on silica gel (eluent: 5% ethyl acetate in DCM) to give the desired product **Bouchardatine** in 65% yield (36 mg). The pure product was obtained by column chromatography on silica gel using 5% MeOH in DCM as eluent. 36 mg (65%); Yellow solid; The ^{1}H NMR and ^{13}C NMR spectra for this compound are consistent with previously reported literature data.[23] ^{1}H-NMR (DMSO-d$_6$, 600 MHz): δ 13.60 (s, 1H), 13.10 (s, 1H), 10.46 (s, 1H), 8.25 (d, *J* = 8.4 Hz, 1H), 8.20 (d, *J* = 9.0 Hz, 1H), 7.92-7.89 (m, 1H), 7.84 (d, *J* = 8.4 Hz, 1H), 7.68 (d, *J* = 8.4 Hz, 1H), 7.58-7.60 (m, 1H), 7.39-7.41 (m, 1H), 7.32-7.34 (m, 1H); ^{13}C-NMR (DCMSO-d$_6$, 150 MHz): δ 187.5, 161.2, 148.4, 145.3, 135.8, 135.7, 134.9, 127.6, 127.5, 127.4, 126.1, 125.4, 123.2, 121.8, 120.2, 115.1, 113.3.

Synthesis of Sildenafil in Scheme 5

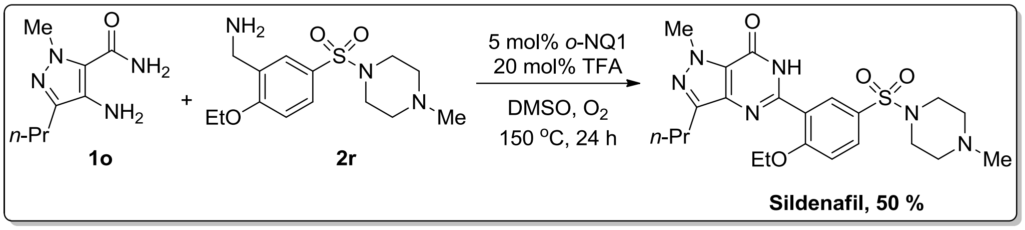

1) Synthesis of Benzyamine Derivative 2r

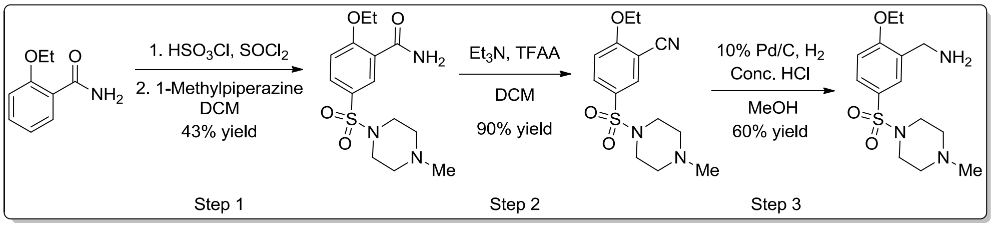

Step 1: To a flask charged with 3 mL of chlorosulfonic acid and 1 mL of Thionyl chloride was added 2-ethoxybenzamide (1.65 g, 10 mmol) at 0 °C under argon. The reaction mixture was stirred for 12 h below 20 °C. After reaction was complete by TLC, the reaction mixture was poured into chopped ice and the resulting product was extracted with dichloromethane (50 mL x 2), dried over Na_2SO_4, filtered and concentrated under reduced pressure. The crude product was dissolved in 20 mL of dry dichloromethane and 1-methylpiperazine (2.44 mL, 20 mmol) was added to the mixture. The reaction was stirred for 30 min at 0 °C and then continued to stir at ambient temperature for 1 h. After the reaction was complete by TLC, 10 mL of water and 20 mL of saturated NH_4Cl were added, respectively.

The reaction mixture was extracted with DCM, dried over Na_2SO_4, filtered, concentrated under reduced pressure. The desired product was purified by recrystallization in diethyl ether to give **2r-Benzamide** in 43% yield.

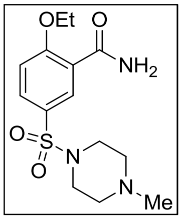

2r-Benzamide (2-Ethoxy-5-((4-methylpiperazin-1-yl)sulfonyl)benzamide): White solid, m.p. 199-200 °C; ^{1}H-NMR ($CDCl_3$, 600 MHz): δ 8.58 (d, J = 2.4 Hz, 1H), 7.82 (dd, J = 8.4, 2.4 Hz, 1H), 7.68 (s, 1H), 7.07 (d, J = 8.4 Hz, 1H), 6.25 (s, 1H), 4.26-4.29 (m, 2H), 2.92-3.16 (m, 4H), 2.38-2.54 (m, 4H), 2.25 (s, 3H), 1.54-1.56 (m, 3H); ^{13}C-NMR ($CDCl_3$, 150 MHz): δ 165.3, 160.3, 133.0, 132.9, 128.1, 121.7, 112.7, 65.8, 54.1, 46.1, 45.8, 14.8; IR(neat): 3451, 3177, 2855, 2808, 1673, 1587, 1343, 1155 cm^{-1}; HRMS (ESI): m/z calcd for $C_{14}H_{22}N_3O_4S$ $[M+H]^+$ 328.1325 Found 328.1338.

Step 2: To a solution of **2r-Benzamide** (1.34 g, 4.1 mmol) in DCM (8mL) was added Et_3N (2.28 mL, 16.4 mmol) at 0 °C under argon. After 5min, trifluoroacetic anhydride (1.27 mL, 9.0 mmol) was added dropwise. The reaction was stirred for 7 h at ambient temperature. The reaction mixture was diluted with DCM (15 mL) and saturated $NaHCO_3$ aqueous solution (5 mL) was added. After extracting with DCM, the combined organic layer was washed with brine, dried over Na_2SO_4, filtered and concentrated under reduced pressure. The desired product was purified by column chromatography on silica gel (eluent: 5% MeOH in DCM) to provide **2r-Nitrile** in 90% yield.

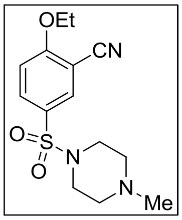

2r-Nitrile (2-Ethoxy-5-((4-methylpiperazin-1-yl)sulfonyl)benzonitrile): Pale Yellow solid, m.p. 131-132 °C; ^{1}H-NMR ($CDCl_3$, 600 MHz): δ 7.90 (d, J = 2.4 Hz, 1H), 7.84 (dd, J = 9.0, 2.4 Hz, 1H), 7.05 (d, J = 9.0 Hz, 1H), 4.19-4.22 (m, 2H), 2.80-3.16 (m, 4H), 2.34-2.55 (m, 4H), 2.23 (s, 3H), 1.47-1.49 (m, 3H); ^{13}C-NMR ($CDCl_3$, 150 MHz): δ 163.5, 134.0, 133.7, 127.8, 114.8, 112.4, 102.9, 65.8, 53.9, 46.0, 45.7, 14.3; IR(neat): 2991, 2804, 2228, 1590, 1490, 1334, 1133 cm^{-1}; HRMS (ESI): m/z calcd for $C_{14}H_{20}N_3O_3S$ $[M+H]^+$ 310.1219 Found 310.1230.

Step 3: **2r-Nitrile** (0.62 g, 2.0 mmol) was dissolved in the mixture of MeOH (10 mL) and conc. HCl (0.8 mL) and 10% Pd/C (120 mg) was added at ambient temperature under argon. The reaction atmosphere was then changed from argon to hydrogen and the solution was stirred for 48 h. After which, the reaction mixture was basified by adding the 1M NaOH solution (20 mL) and diluted with ethyl acetate (30 mL) and filtered through Celite. The filtrate was washed with brine, dried over Na_2SO_4, filtered and concentrated under reduced pressure. The residue was purified by column chromatography on silica gel (eluent: 3% NH_4OH+15% CH_3CN in DCM) to give the compound **2q** in 60% yield.

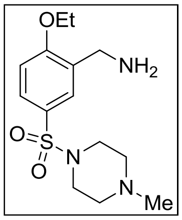

(2-Ethoxy-4-((4-methylpiperazin-1-yl)sulfonyl)phenyl)methanamine (2r): White solid, m.p. 179-180 °C; ^{1}H-NMR ($CDCl_3$, 600 MHz): δ 7.67 (d, *J* = 2.4 Hz, 1H), 7.59 (dd, *J* = 8.4, 2.4 Hz, 1H), 6.89 (d, *J* = 8.4 Hz, 1H), 4.09 (q, *J* = 7.2 Hz, 2H), 3.78 (s, 2H), 2.82-3.10 (m, 4H), 2.37-2.55 (m, 4H), 2.23 (s, 3H), 1.41 (t, *J* = 7.2 Hz, 3H); ^{13}C-NMR ($CDCl_3$, 150 MHz): δ 160.5, 129.6, 129.0, 128.8, 126.4, 110.9, 64.2, 54.1, 47.9, 46.1, 45.8, 14.7; IR(neat): 3354, 2937, 2851, 2791, 1602, 1349, 1121 cm^{-1}; HRMS (ESI): m/z calcd for $C_{14}H_{24}N_3O_3S$ $[M+H]^+$ 314.1532 Found 314.1540.

2) Synthesis and Characterization of Sildenafil

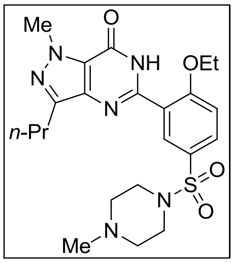

Sildenafil (5-(2-ethoxy-4-((4-methylpiperazin-1-yl)sulfonyl)phenyl)-1-methyl-3-propyl-6,7a-dihydro-1*H*-pyrazolo[4,3-*d*]pyrimidin-7(3a*H*)-one): Sildenafil was prepared by the General Procedure A (150 °C, 24 h) using **1o** (0.2 mmol, 36.4 mg) and **2r** (0.24 mmol, 75 mg). The residue was purified by column chromatography on silica gel (eluent: 1% MeOH in dichloromethane). 47 mg (50%); white solid; The ^{1}H NMR and ^{13}C NMR spectra for this compound are consistent with previously reported literature data.[21] ^{1}H-NMR ($CDCl_3$, 600 MHz): δ 10.82 (s, 1H), 8.81 (d, *J* = 2.4 Hz, 1H), 7.82 (dd, *J* = 8.4, 2.4 Hz, 1H), 7.14 (d, *J* = 8.4 Hz, 1H), 4.34-4.38 (m, 2H), 4.27 (s, 3H), 3.10 (m, 4H), 2.90-2.93 (m, 2H), 2.50 (m, 4H), 2.27 (s, 3H), 1.83-1.86 (m, 2H), 1.62-1.64 (m, 3H), 1.01 (t, *J* = 7.8 Hz, 3H); ^{13}C-NMR ($CDCl_3$, 150 MHz): δ 159.4, 153.8, 147.1, 146.5, 138.5, 131.8, 131.3, 129.0, 124.6, 121.2, 113.2, 66.2, 54.2, 46.1, 45.8, 38.4, 27.9, 22.4, 14.7, 14.2.

References

1. Parua, S. P.; Das, S.; Sikari, R.; Sinha, S.; Paul, N. D. One-Pot Cascade Synthesis of Quinazolin-4(3*H*)-ones via Nickel-Catalyzed Dehydrogenative Coupling of *o*-Aminobenzamides with Alcohols. *J. Org. Chem.* **2017**, *82*, 7165-7175.
2. Dhiman, S.; Saini, H. K.; Nandwana, N. K.; Kumar, D. Copper-Catalyzed Synthesis of Quinoline Derivatives *via* Tandem Knoevenagel Condensation, Amination and Cyclization. *RSC Adv*. **2016**, *6*, 23987-23994.
3. Tu, T.; Wang, Z.; Liu, Z.; Feng, X.; Wang, Q. Efficient and Practical Transition Metal-Free Catalytic

Hydration of Organonitriles to amides. *Green Chem.* **2012**, *14*, 921-924.

4. Mizutani, T.; Nagase, T.; Ito, S.; Miyamoto, Y.; Tanaka, T.; Takenaga, N.; Tokita, S.; Sato, N. Development of Novel 2-[4-(Aminoalkoxy)phenyl]-4(3*H*)-Quinazolinone Derivatives as Potent and Selective Histamine H_3 Receptor Inverse Agonists. *Bioorg. Med. Chem.* **2008**, *18*, 6041-6045.
5. Sutherell, C.; Ley, S. V. On the Synthesis and Reactivity of 2,3-Dihydropyrrolo[1,2-a]quinazolin-5(1*H*)-ones. *Synthesis* **2017**, *49*, 135-144.
6. Long, L.; Wang, Y. H.; Zhuo, J. X.; Tu, Z. C.; Wu, R.; Yan, M.; Liu, Q.; Lu, G. Structure-Based Drug Design: Synthesis and Biological Evaluation of Quinazolin-4-amine Derivatives as Selective Aurora A kinase inhibitors. *Eur. J. Med. Chem.* **2018**, *157*, 1361-1375.
7. Nathubhai, A.; Haikarainen, T.; Hayward, P. C.; Muñoz-Descalzo, S.; Thompson, A. S.; Lloyd, M. D.; Lehtiö, L.; Threadgill, M. D. Structure-Activity Telationships of 2-Arylquinazolin-4-ones as Highly Selective and Potent Inhibitors of the Tankyrases. *Eur. J. Med. Chem.* **2016**, *118*, 316-327.
8. Wang, Z.; Tang, Y. Mechanistic Insights into a Catalyst-Free Method to Construct Quinazolinones through Multiple Oxidative Cyclization. *Tetrahedron* **2016**, *72*, 1330-1336.
9. (a) Kim, H. Y.; Oh, K. A Facile Access to 4-Substituted-2-naphthols *via* a Tandem Friedel-Crafts Reaction: A *β*-Chlorovinyl Ketone Pathway. *Org. Lett.* **2014**, *16*, 5934-5936; (b) Kim, H. Y.; Takizawa, S.; Oh, K. Copper-catalyzed Divergent Oxidative Pathways of 2-Naphthol Derivatives: *ortho*-Naphthoquinones *versus* 2-BINOLs. *Org. Biomol. Chem.* **2016**, *14*, 7191-7196; (c) Goriya, Y.; Kim, H. Y.; Oh, K. *o*-Naphthoquinone-catalyzed Aerobic Oxidation of Amines to (Ket)imines: A Modular Catalyst Approach. *Org. Lett.* **2016**, *18*, 5174−5177.
10. Hikawa, H.; Ino, Y.; Suzuki, H.; Yokoyama, Y. Pd-Catalyzed Benzylic C–H Amidation with Benzyl Alcohols in Water: A Strategy to Construct Quinazolinones. *J. Org. Chem.* **2012**, *77*, 7046-7051.
11. Zhou, J.; Fang, J. One-pot Synthesis of Quinazolinones via Iridium-Catalyzed Hydrogen Transfers. *J. Org. Chem.* **2011**, *76*, 7730-7736.
12. Tian, X.; Song, L.; Li, E.; Wang, Q.; Yu, W.; Chang, J. Metal-free One-pot Synthesis of 1,3-Diazaheterocyclic Compounds via I_2-Mediated Oxidative C–N Bond Formation. *RSC. Adv.* **2015**, *5*, 62194-62201.
13. Hu, Y.; Chen, L.; Li, B. Iron Nitrate/TEMPO-catalyzed Aerobic Oxidative Synthesis of Quinazolinones

from Alcohols and 2-Aminobenzamides with Air as the Oxidant. *RSC. Adv.* **2016**, *6*, 65196-65204.

14. Liu, W.; Gao, W.; Ding, J.; Huang, X.; Liu, M.; Wu, H. Palladium-catalyzed Oxidative C=C Bond Cleavage with Molecular Oxygen: One-pot Synthesis of Quinazolinones from 2-Amino Benzamides and Alkenes. *Org. Chem. Front.* **2018**, *5*, 2734-2738.
15. Iqbal, M. A.; Lu, L.; Mehmood, H.; Khan, D. M.; Hua, R. Quinazolinone Synthesis through Base-Promoted S_NAr Reaction of *ortho*-Fluorobenzamides with Amides Followed by Cyclization. *ACS Omega,* **2019**, *4*, 8207-8213.
16. Wang, Q.; Lv, M.; Liu, J.; Li, Y.; Xu, Q.; Zhang, X.; Cao, H. Efficient Synthesis of Quinazolinones by Transition-Metal-Free Direct Aerobic Oxidative Cascade Annulation of Alcohols with *o*-Aminoarylnitriles. *ChemSusChem*, **2019**, *12*, 3043-3048.
17. Xu, W.; Jin, Y.; Liu, H.; Jiang, Y.; Fu, H. Copper-Catalyzed Domino Synthesis of Quinazolinones via Ullmann-Type Coupling and Aerobic Oxidative C-H Amidation. *Org. Lett.* **2011**, *13*, 1274-1277.
18. Yang, X.; Cheng, G.; Shen, J.; Kuai, C.; Cui, X. Cleavage of the C-C Triple Bond of Ketoalkynes: Synthesis of 4(3*H*)-Quinazolinones. *Org. Chem. Front.* **2015**, *2*, 366-368.
19. Chen, K.; Gao, B.; Shang, Y.; Du, J.; Gu, Q.; Wang, J. I_2-Catalyzed Cross Dehydrogenative Coupling: Rapid Access to Benzoxazinones and Quinazolinones. *Org. Biomol. Chem.* **2017**, *15*, 8770-8779.
20. Krapf, M. K.; Gallus, J.; Vahdati, S.; Wises, M. New Inhibitors of Breast Cancer Resistance Protein (ABCG2) Containing a 2,4-Disubstituted Pyridopyrimidine Scaffold. *J. Med. Chem.* **2018**, *61*, 3389-3408.
21. Laha, J. K.; Patel, K. V.; Tummalapalli, S.; Dayal, N. Formation of Amides, their Intramolecular Reactions for the Synthesis of *N*-Heterocycles, and Preparation of a Marketed Drug, Sildenafil: A Comprehensive Coverage. *Chem. Commun.* **2016**, *52*, 10245-10248.
22. Liu, H.; Zhai, T.; Ding, S.; Hou, Y.; Zhang, X.; Feng, L.; Ma, C. Direct and Metal-Free Oxidative Amination of sp^3 C-H bonds for the Construction of 2-hetarylquinazolin-4(3*H*)-ones. *Org. Chem. Front.* **2016**, *3*, 1096-1099.
23. Viji, M.; Nagarajan, R. Copper-Catalysed Synthesis of Indolylquinazolinone Alkaloid Bouchardatine. *J. Chem. Sci.* **2014**, *126*, 1075-1080.

Spectra:

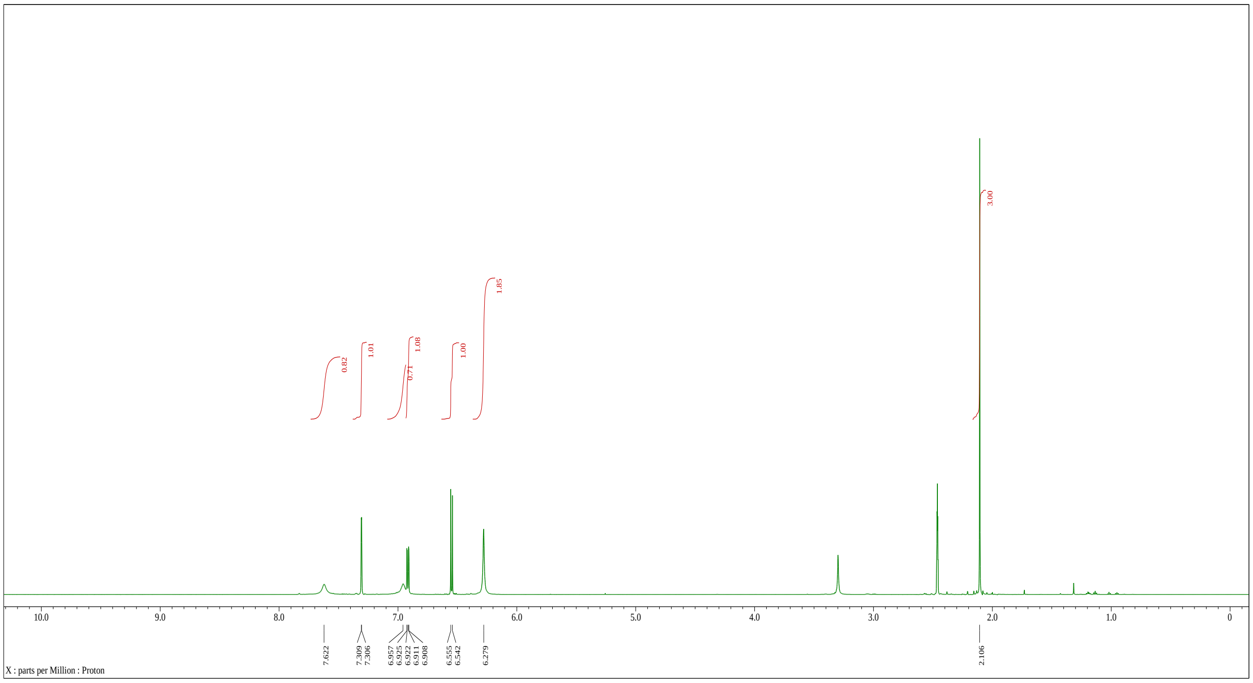

0.82
1.01
0.71
1.08
1.00
1.85
3.00
10.0
9.0
8.0
7.0
6.0
5.0
4.0
3.0
2.0
1.0
0
7.622
7.309
7.306
6.957
6.925
6.922
6.911
6.908
6.555
6.542
6.279
2.106
X : parts per Million : Proton

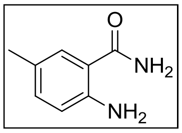

O
NH2
NH2

1d

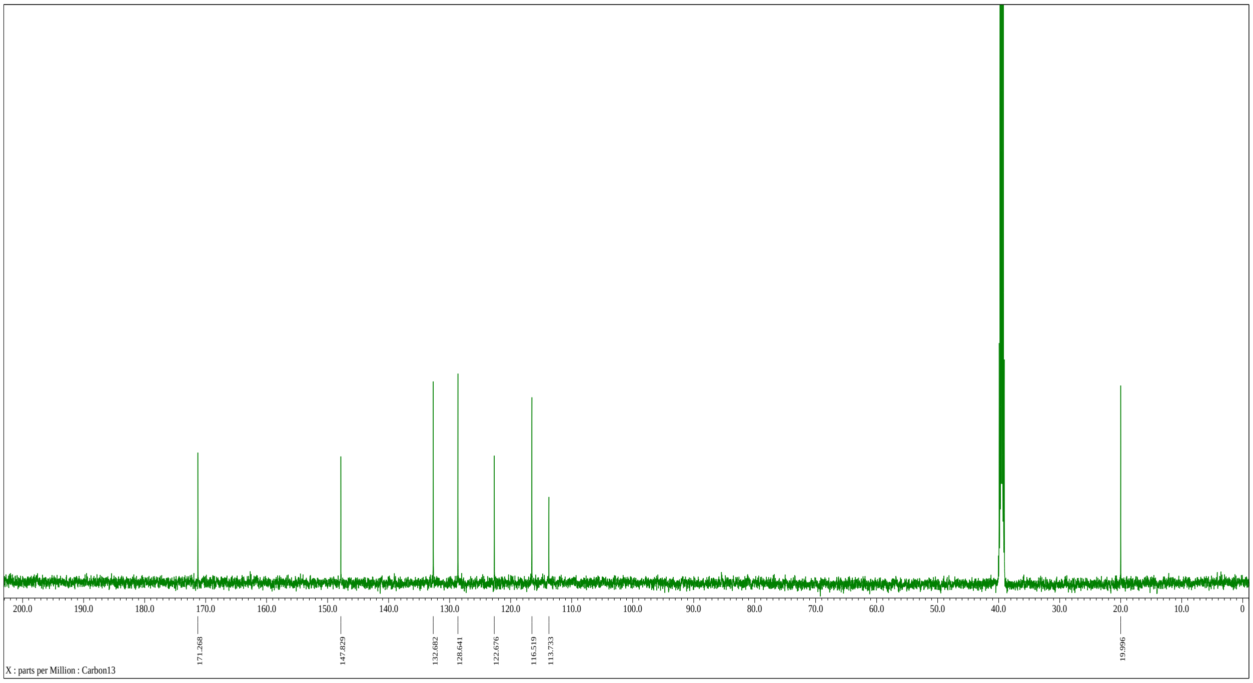

200.0
190.0
180.0
170.0
160.0
150.0
140.0
130.0
120.0
110.0
100.0
90.0
80.0
70.0
60.0
50.0
40.0
30.0
20.0
10.0
0
171.268
147.829
132.682
128.641
122.676
116.519
113.733
19.996
X : parts per Million : Carbon13

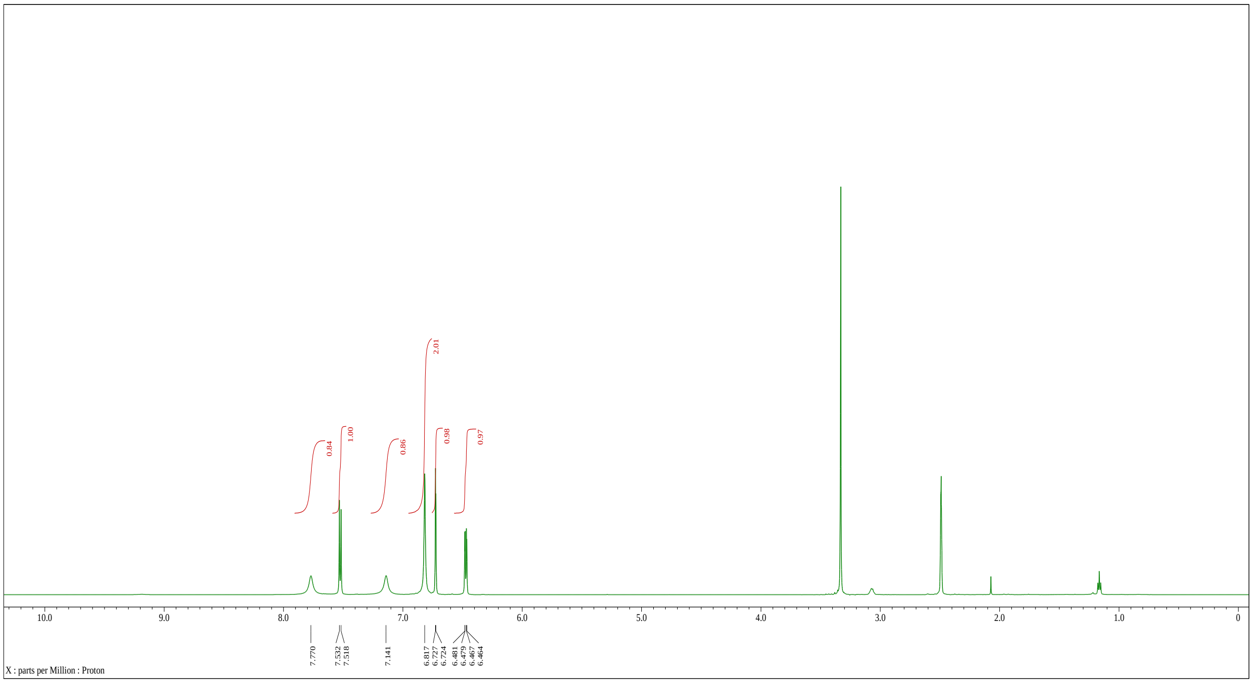
0.84
1.00
0.86
2.01
0.98
0.97
10.0
9.0
8.0
7.0
6.0
5.0
4.0
3.0
2.0
1.0
0
7.770
7.532
7.518
7.141
6.817
6.727
6.724
6.481
6.479
6.467
6.464
X : parts per Million : Proton

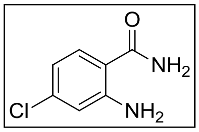
O
NH2
Cl
NH2

1e

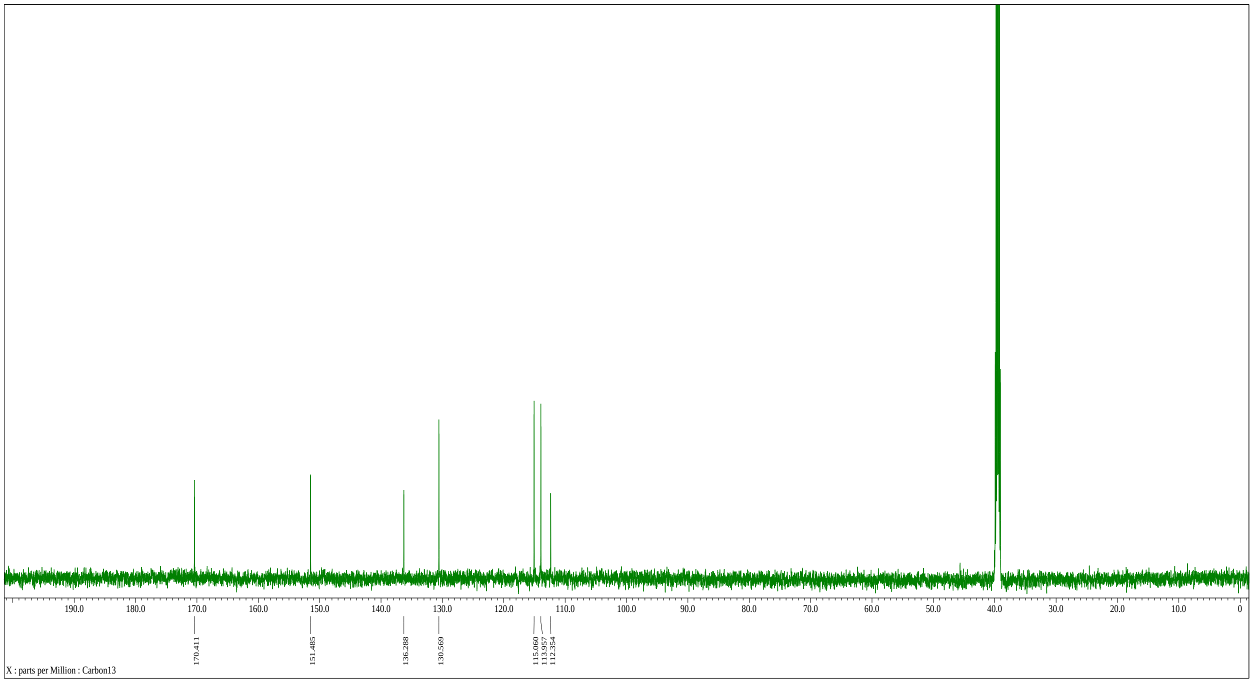
190.0
180.0
170.0
160.0
150.0
140.0
130.0
120.0
110.0
100.0
90.0
80.0
70.0
60.0
50.0
40.0
30.0
20.0
10.0
0
170.411
151.485
136.288
130.569
115.060
113.957
112.354
X : parts per Million : Carbon13

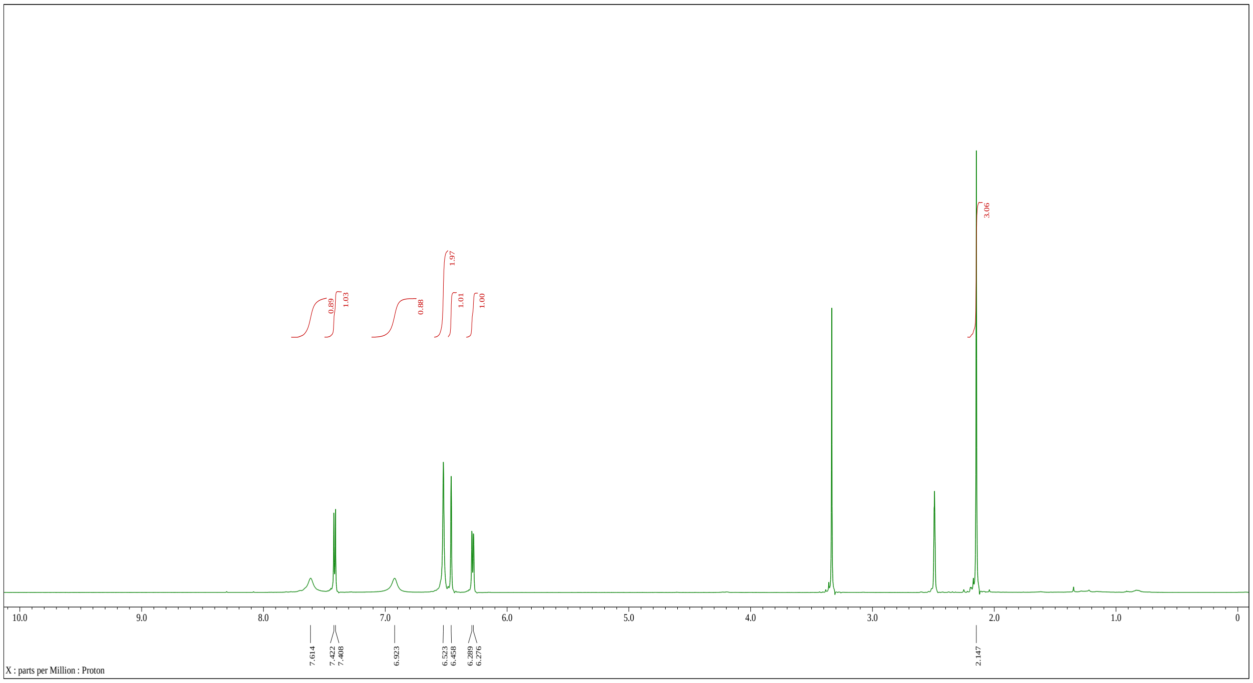
0.89
1.03
0.88
1.97
1.01
1.00
3.06
10.0
9.0
8.0
7.0
6.0
5.0
4.0
3.0
2.0
1.0
0
7.614
7.422
7.408
6.923
6.523
6.458
6.289
6.276
2.147
X : parts per Million : Proton

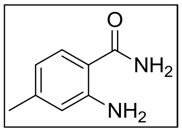
O
NH2
NH2

1f

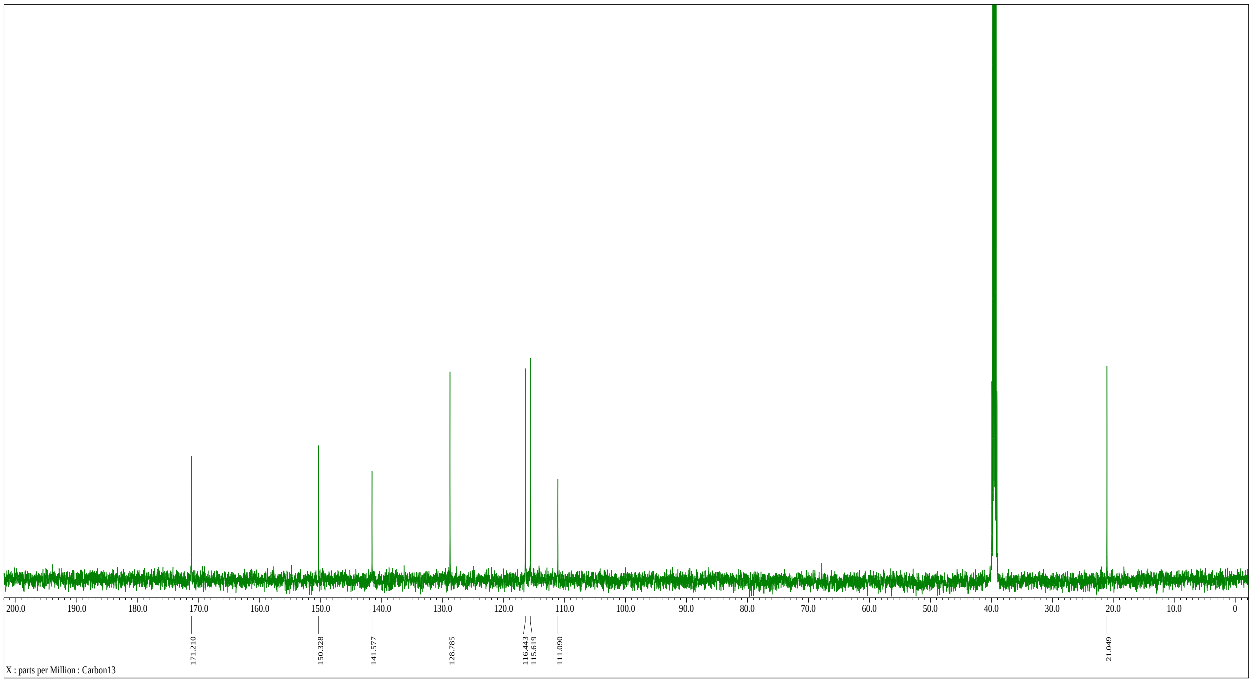
200.0
190.0
180.0
170.0
160.0
150.0
140.0
130.0
120.0
110.0
100.0
90.0
80.0
70.0
60.0
50.0
40.0
30.0
20.0
10.0
0
171.210
150.328
141.577
128.785
116.443
115.619
111.090
21.049
X : parts per Million : Carbon13

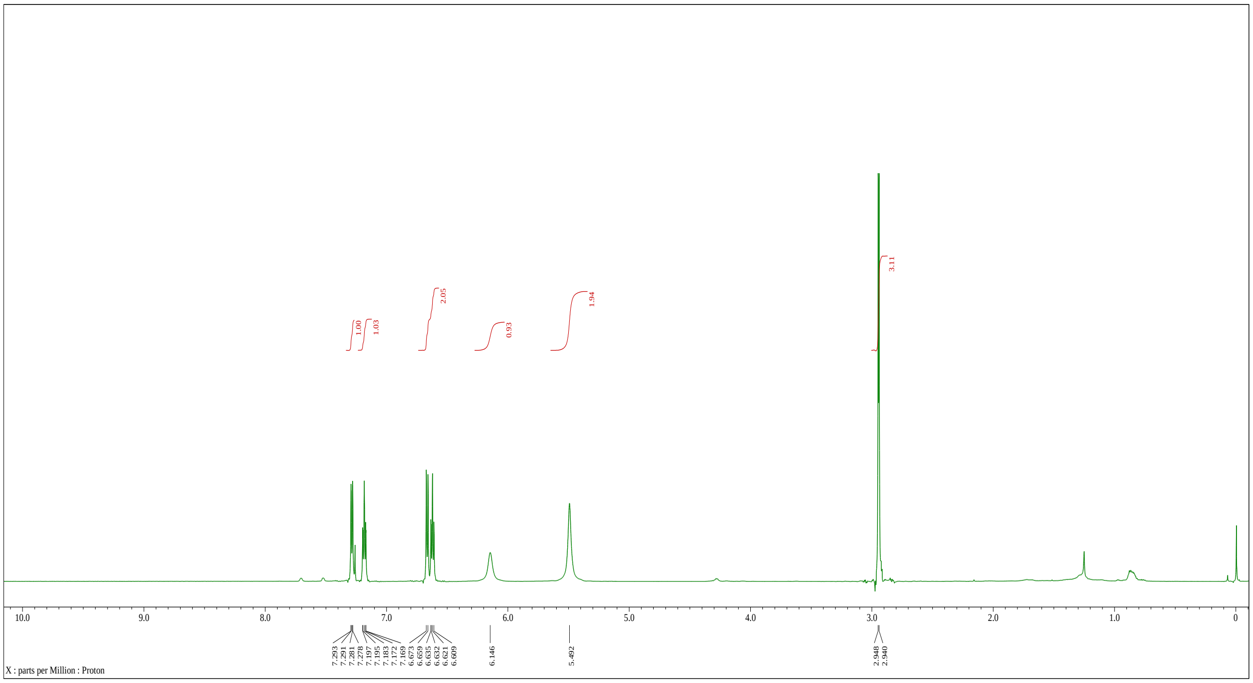
7.293
7.291
7.281
7.278
7.197
7.195
7.183
7.172
7.169
6.673
6.659
6.635
6.632
6.621
6.609
6.146
5.492
2.948
2.940
1.00
1.03
2.05
0.93
1.94
3.11
X : parts per Million : Proton

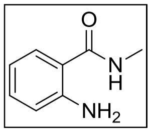
O
N
H
NH2

1g

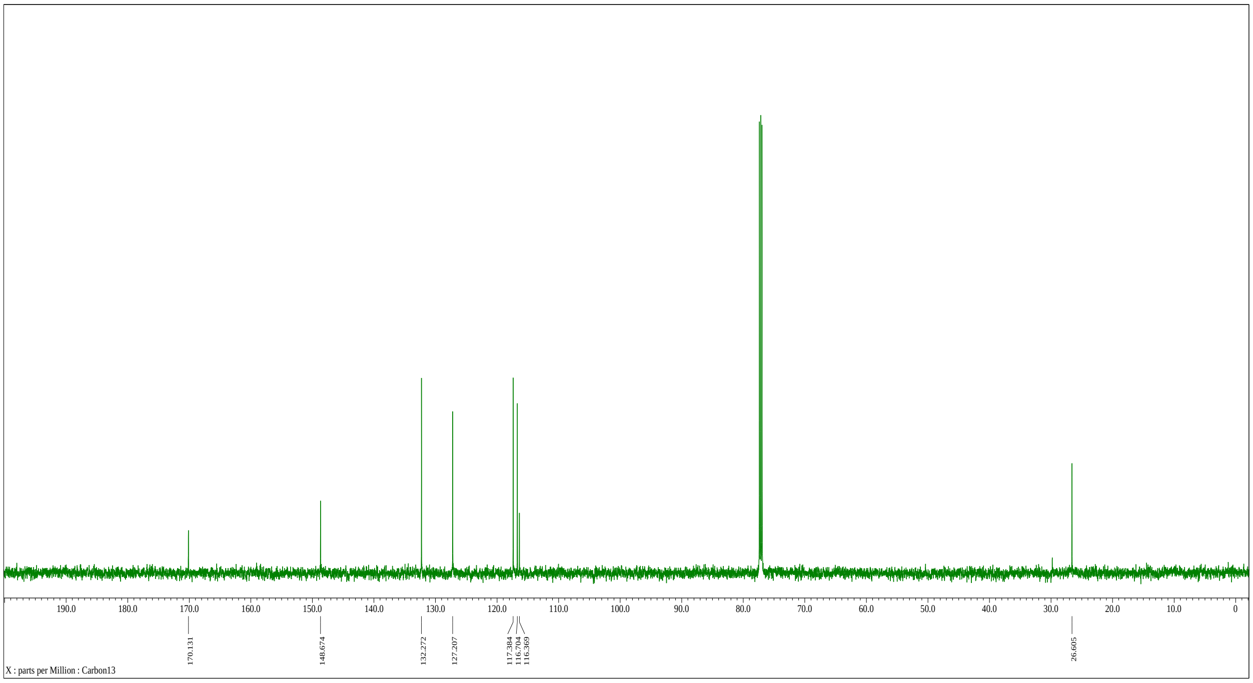
170.131
148.674
132.272
127.207
117.384
116.704
116.369
26.605
X : parts per Million : Carbon13

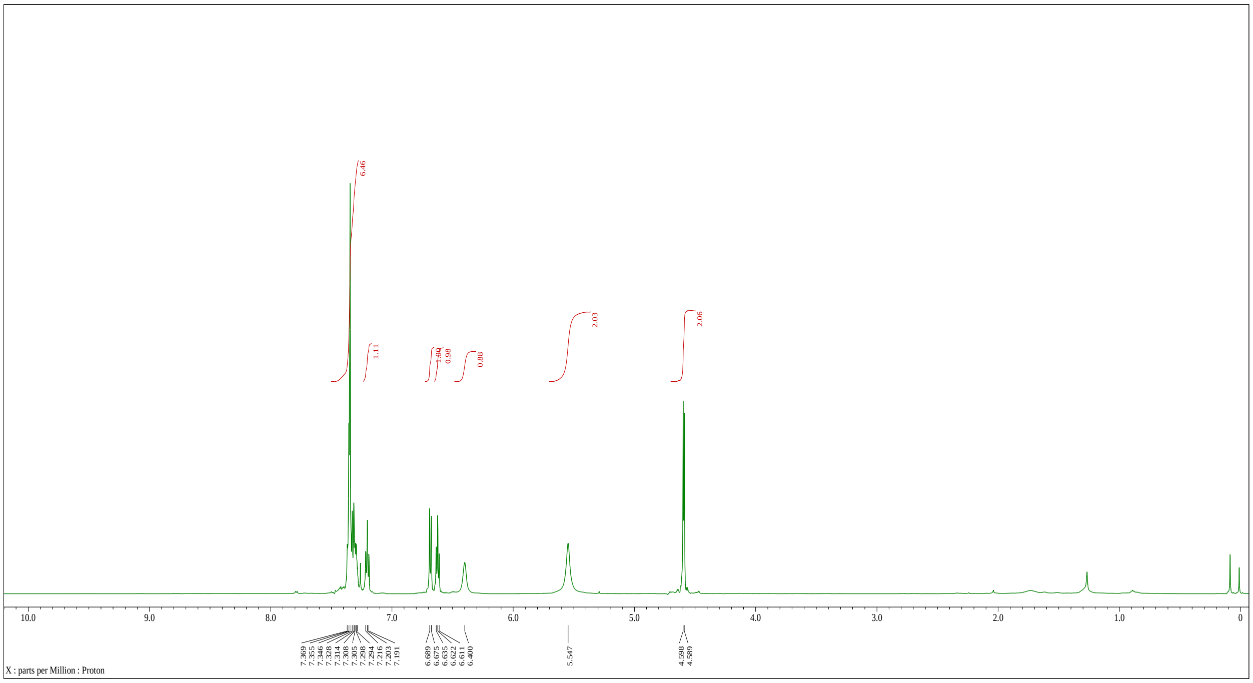
6.46
1.11
1.00
0.98
0.88
2.03
2.06
10.0
9.0
8.0
7.0
6.0
5.0
4.0
3.0
2.0
1.0
0
7.369
7.355
7.346
7.328
7.314
7.308
7.305
7.298
7.294
7.216
7.203
7.191
6.689
6.675
6.635
6.622
6.611
6.400
5.547
4.598
4.589
X : parts per Million : Proton

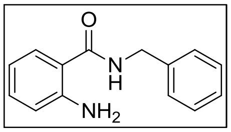
O
N
H
NH2

1h

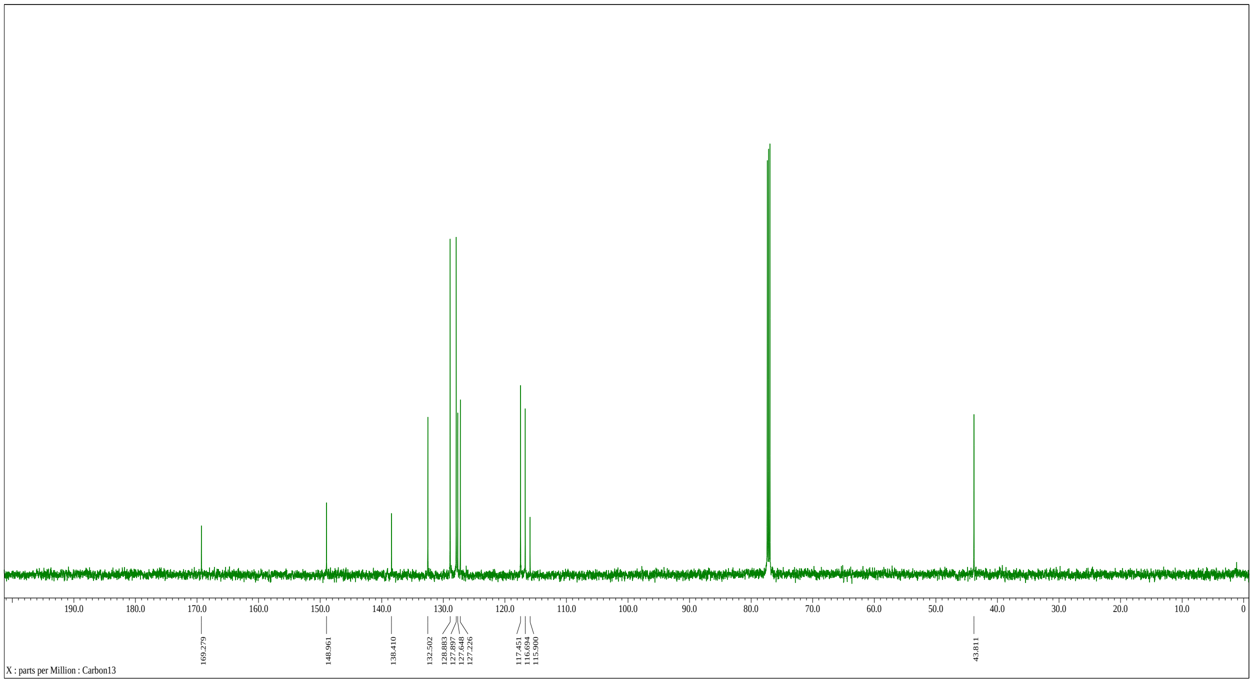
190.0
180.0
170.0
160.0
150.0
140.0
130.0
120.0
110.0
100.0
90.0
80.0
70.0
60.0
50.0
40.0
30.0
20.0
10.0
0
169.279
148.961
138.410
132.502
128.883
127.897
127.648
127.226
117.451
116.694
115.960
43.811
X : parts per Million : Carbon13

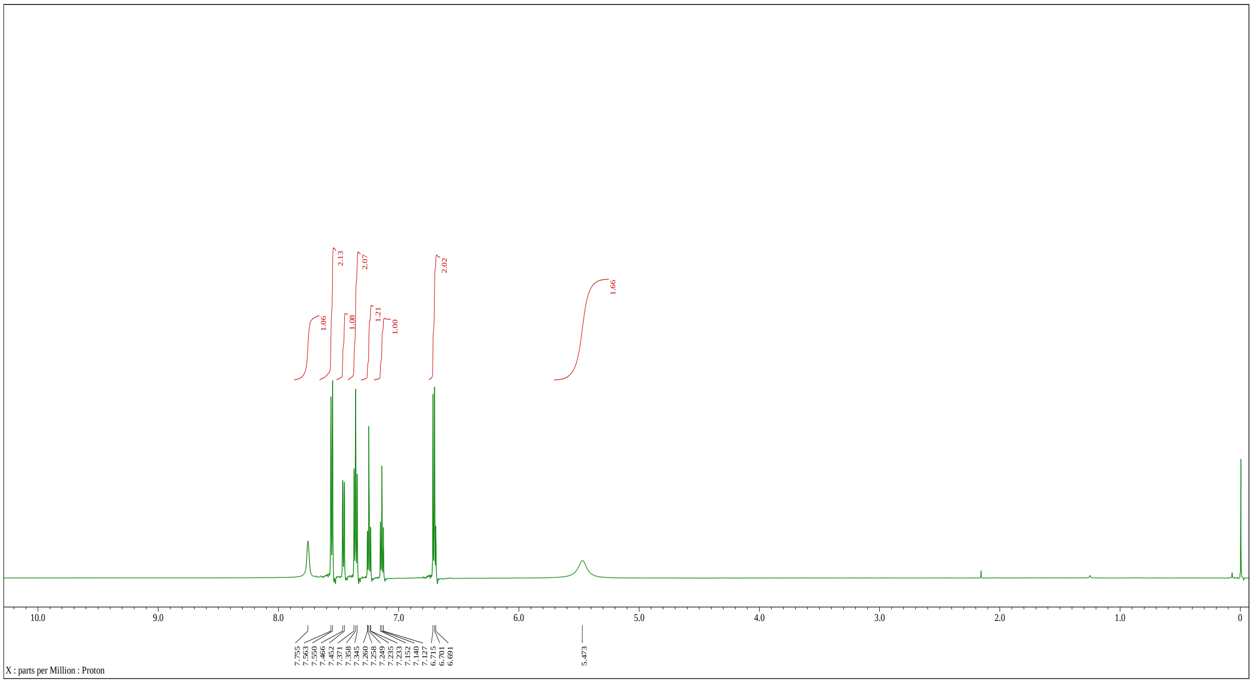
1.06
2.13
1.08
2.07
1.21
1.00
2.02
1.66
7.755
7.563
7.550
7.466
7.452
7.371
7.358
7.345
7.260
7.258
7.249
7.235
7.233
7.152
7.140
7.127
6.715
6.701
6.691
5.473
X : parts per Million : Proton

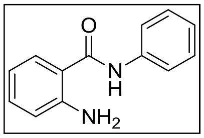
O
N
H
NH2

1i

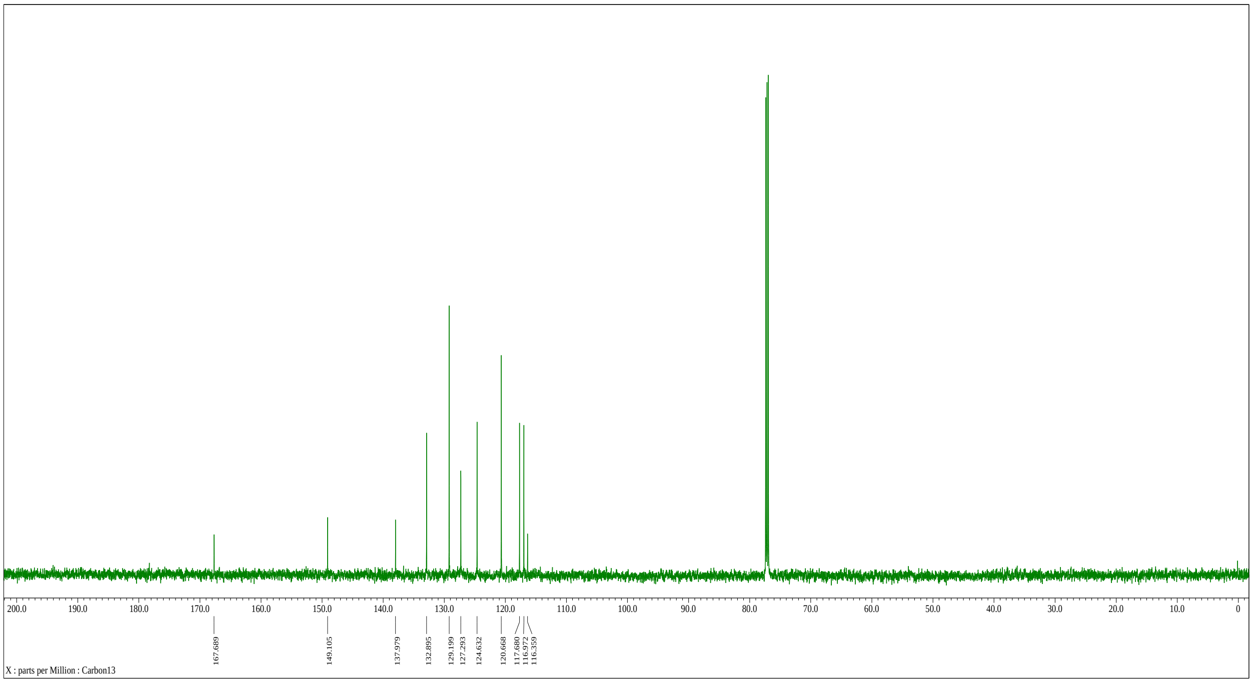
167.689
149.105
137.979
132.895
129.199
127.293
124.632
120.668
117.680
116.972
116.359
X : parts per Million : Carbon13

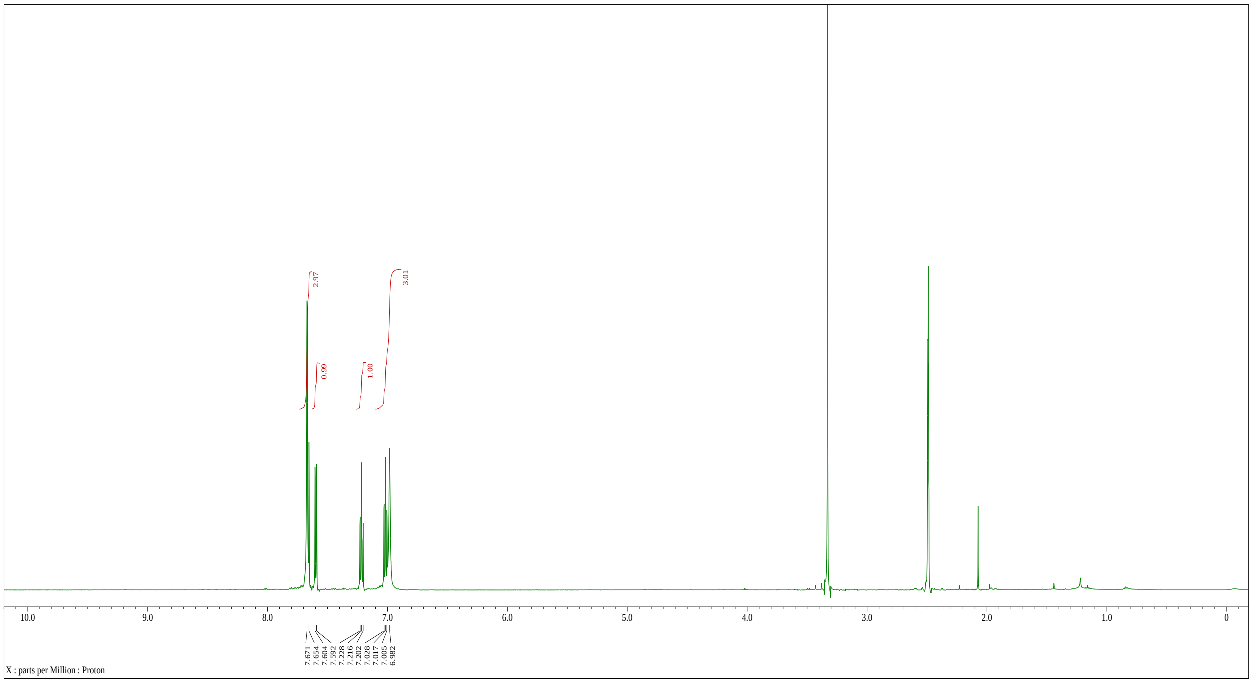
2.97
0.99
1.00
3.01
10.0
9.0
8.0
7.0
6.0
5.0
4.0
3.0
2.0
1.0
0
7.671
7.654
7.604
7.592
7.228
7.216
7.202
7.028
7.017
7.005
6.982
X : parts per Million : Proton

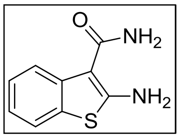
O
NH2
NH2
S

1l

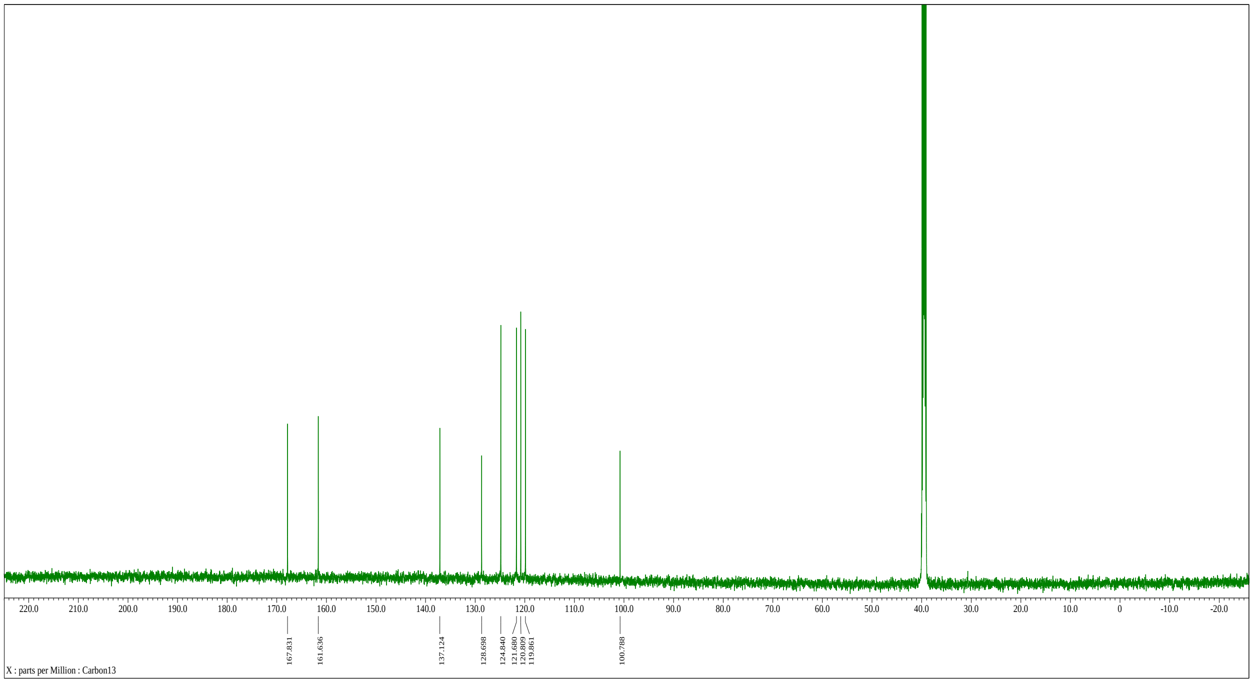
220.0
210.0
200.0
190.0
180.0
170.0
160.0
150.0
140.0
130.0
120.0
110.0
100.0
90.0
80.0
70.0
60.0
50.0
40.0
30.0
20.0
10.0
0
-10.0
-20.0
167.831
161.636
137.124
128.698
124.840
121.680
120.809
119.861
100.788
X : parts per Million : Carbon13

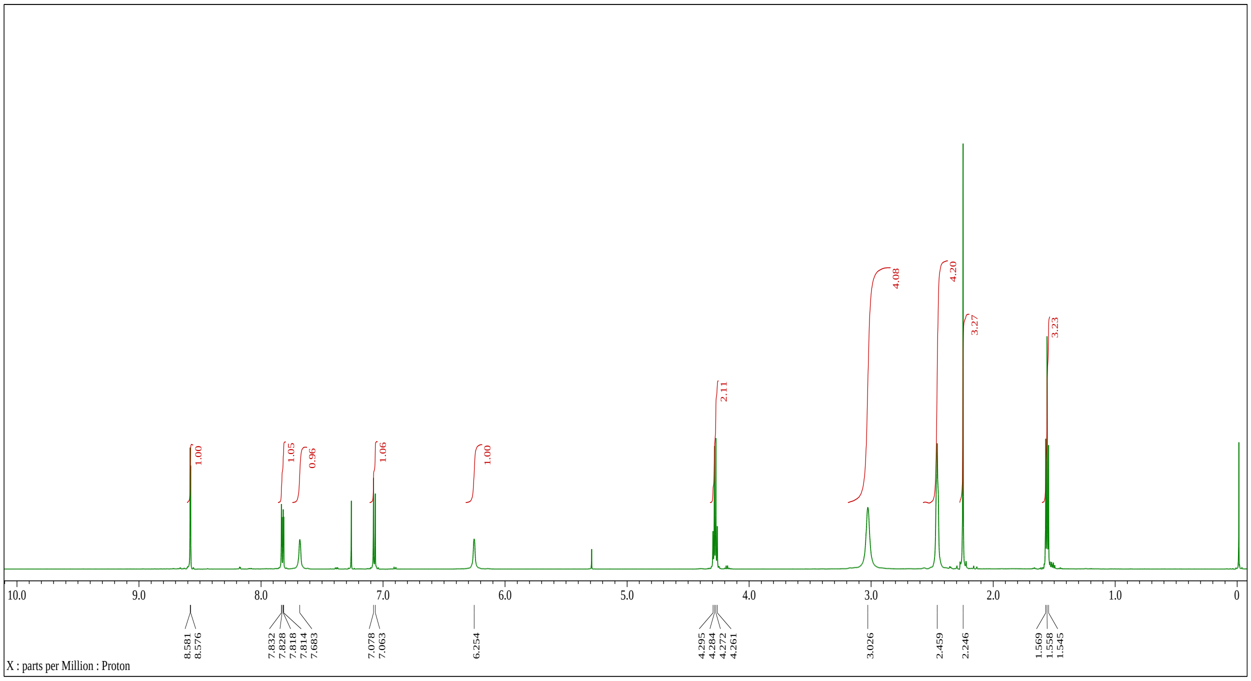

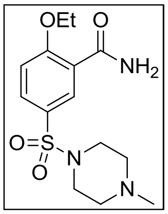

2r-Benzamide

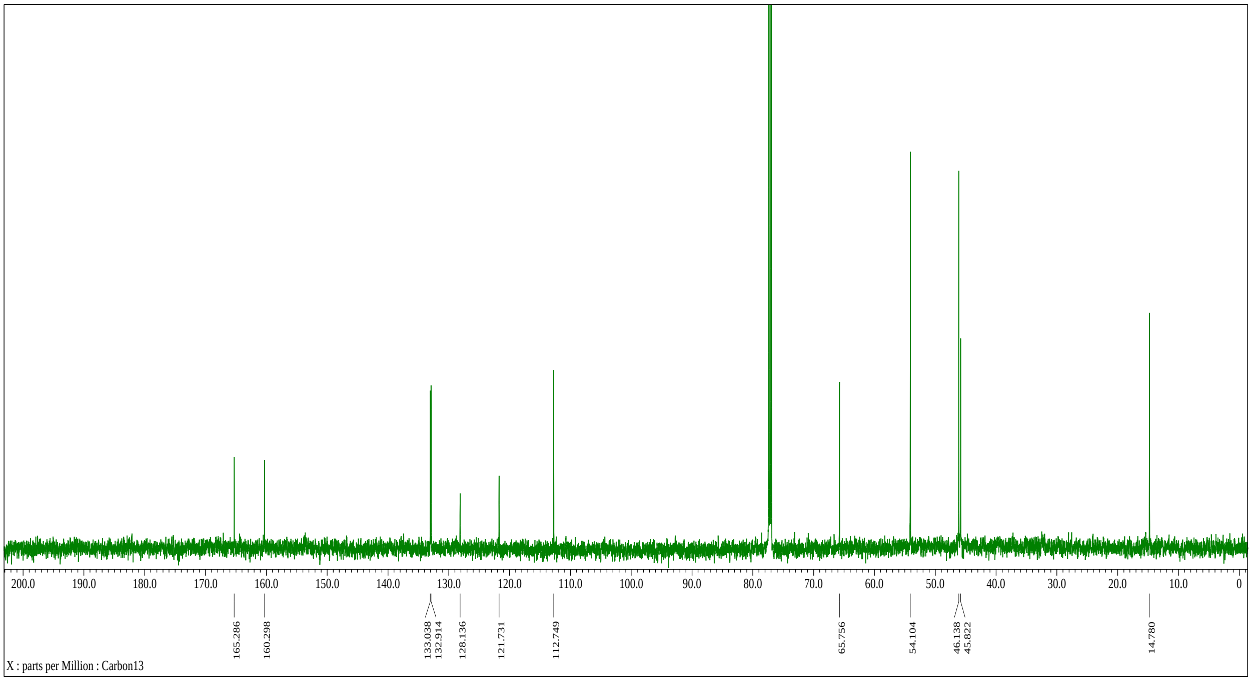

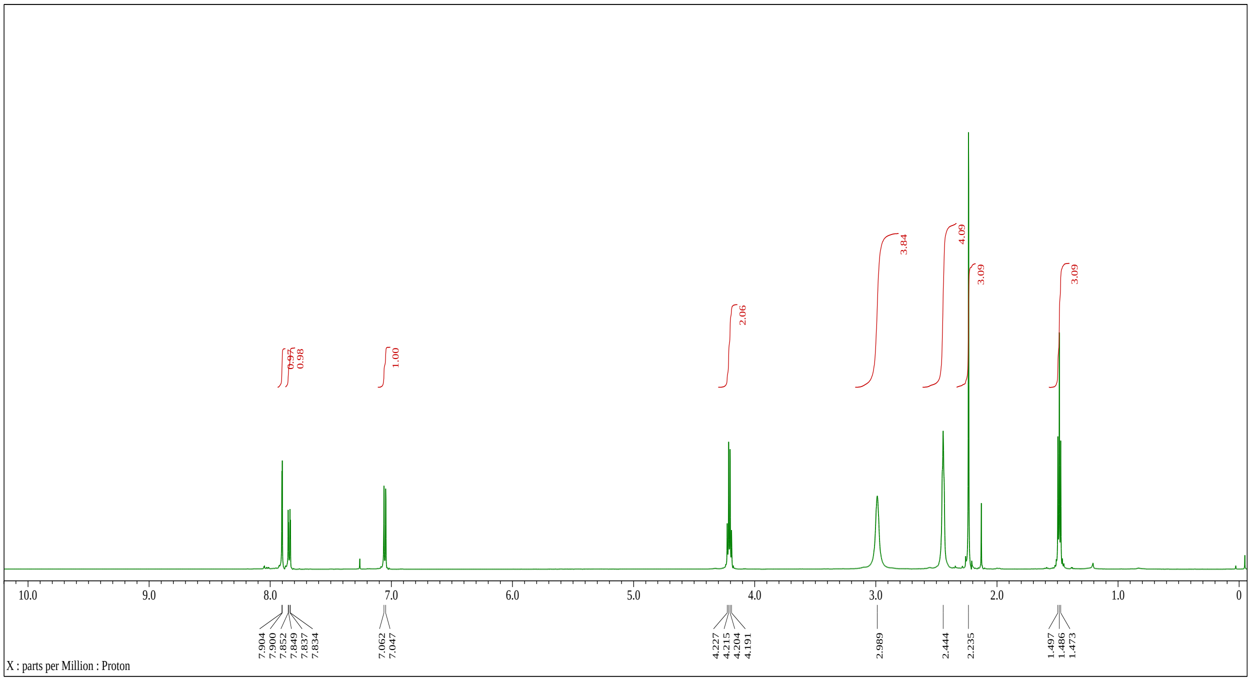

X : parts per Million : Proton
10.0 9.0 8.0 7.0 6.0 5.0 4.0 3.0 2.0 1.0 0
7.904 7.900 7.852 7.849 7.837 7.834
7.062 7.047
4.227 4.215 4.204 4.191
2.989
2.444
2.235
1.497 1.486 1.473
0.98 1.00 2.06 3.84 4.09 3.09 3.09

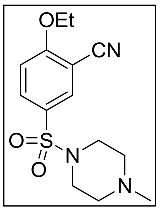

OEt
CN
O=S
O
N
N

2r-Nitrile

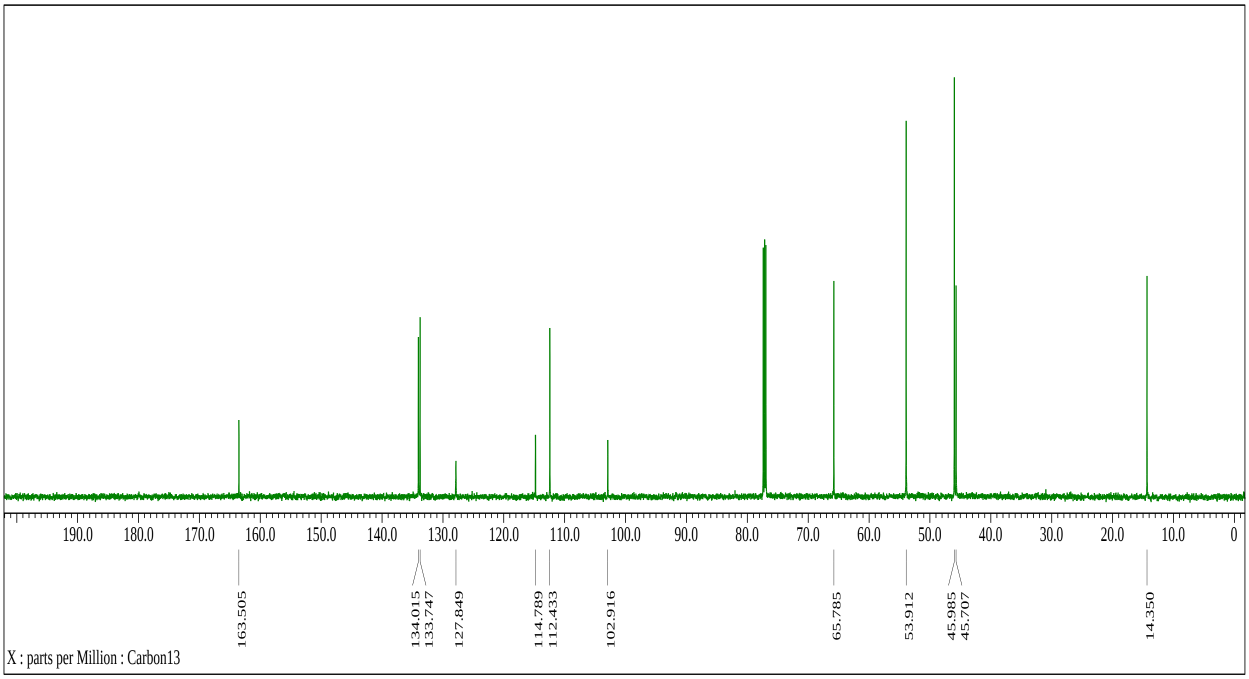

X : parts per Million : Carbon13
190.0 180.0 170.0 160.0 150.0 140.0 130.0 120.0 110.0 100.0 90.0 80.0 70.0 60.0 50.0 40.0 30.0 20.0 10.0 0
163.505
134.015 133.747
127.849
114.789 112.433
102.916
65.785
53.912
45.985 45.707
14.350

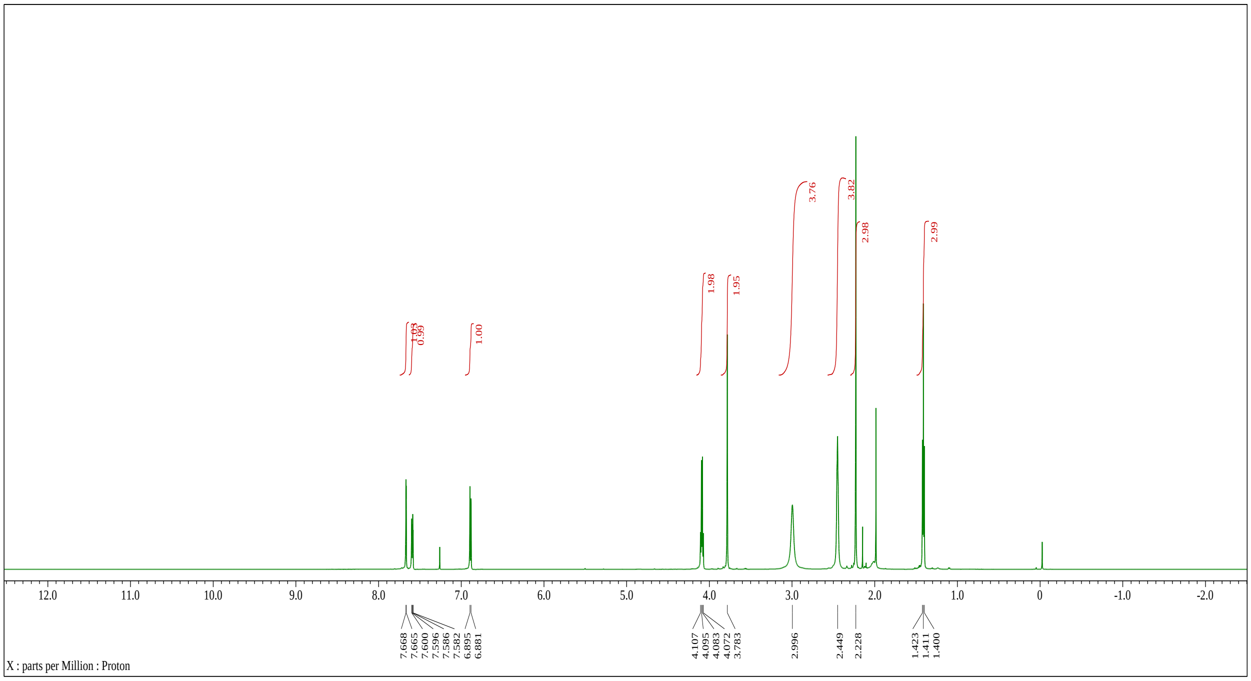
X : parts per Million : Proton

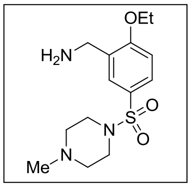
OEt
H_2N
S
O
O
N
N
Me

2r

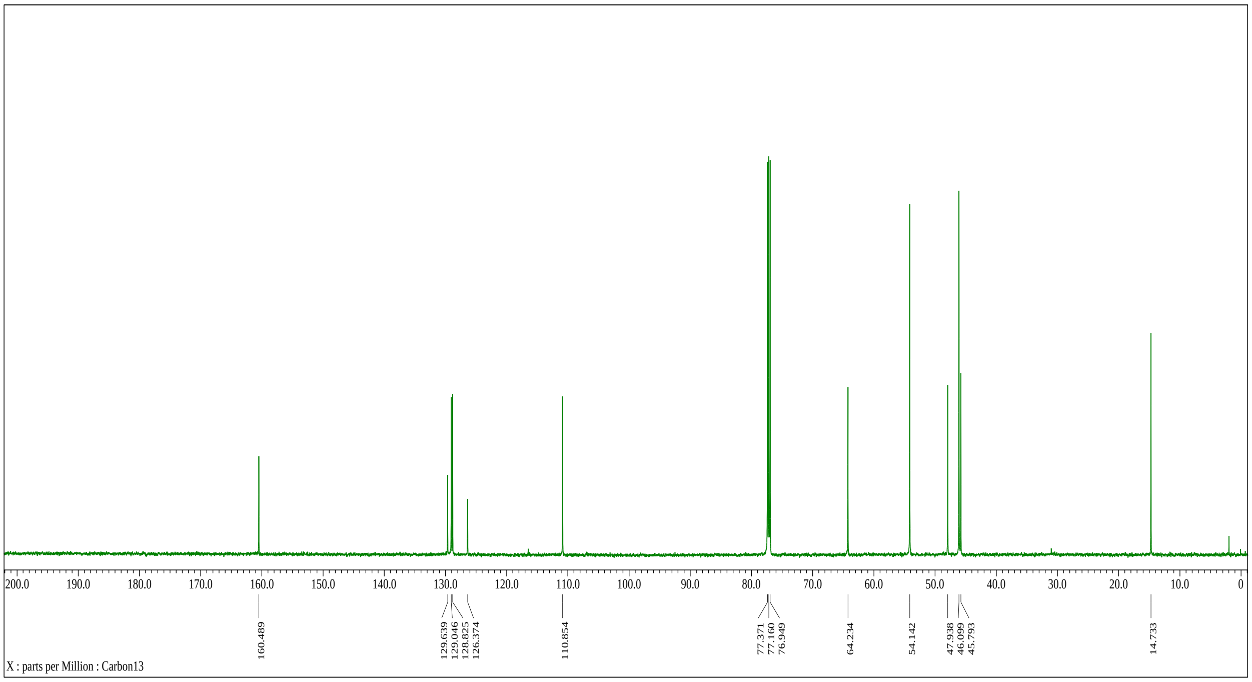
X : parts per Million : Carbon13

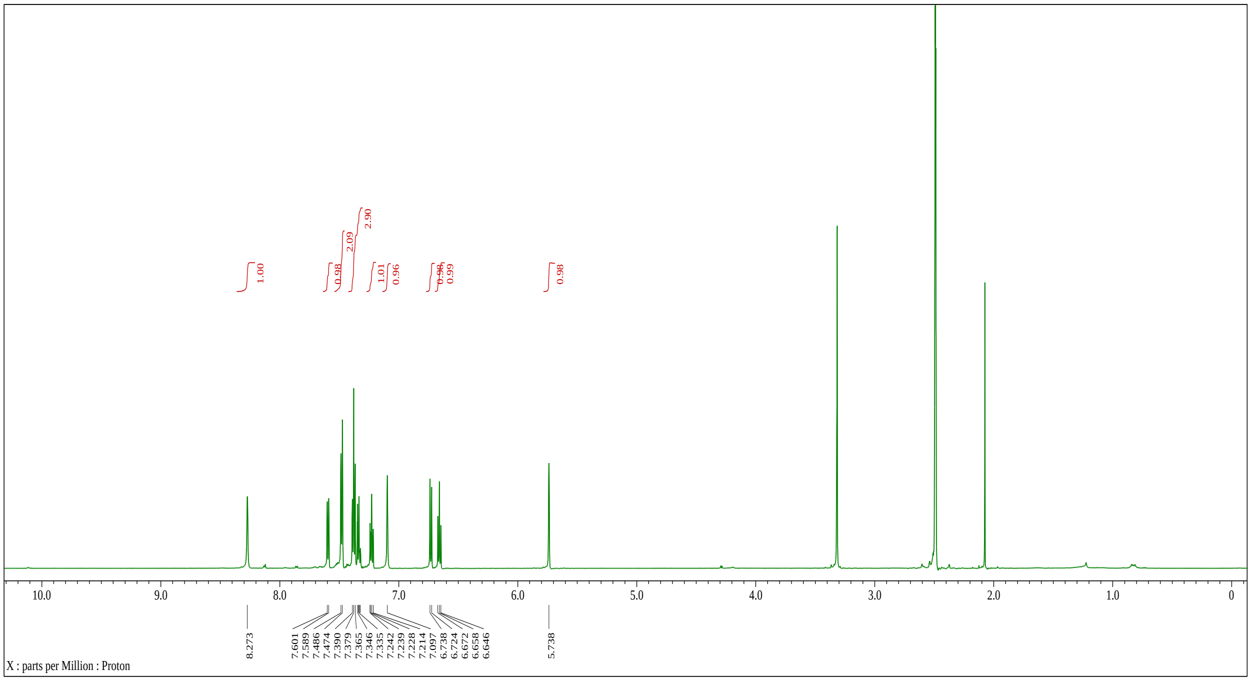

1.00
0.98
2.09
2.90
1.01
0.96
0.98
0.99
0.98
10.0 9.0 8.0 7.0 6.0 5.0 4.0 3.0 2.0 1.0 0
8.273
7.601 7.589 7.486 7.474 7.390 7.379 7.365 7.346 7.335 7.242 7.239 7.228 7.214 7.097 6.738 6.724 6.672 6.658 6.646
5.738
X : parts per Million : Proton

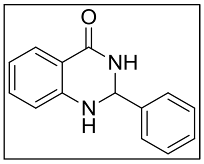

O
NH
N
H

3a

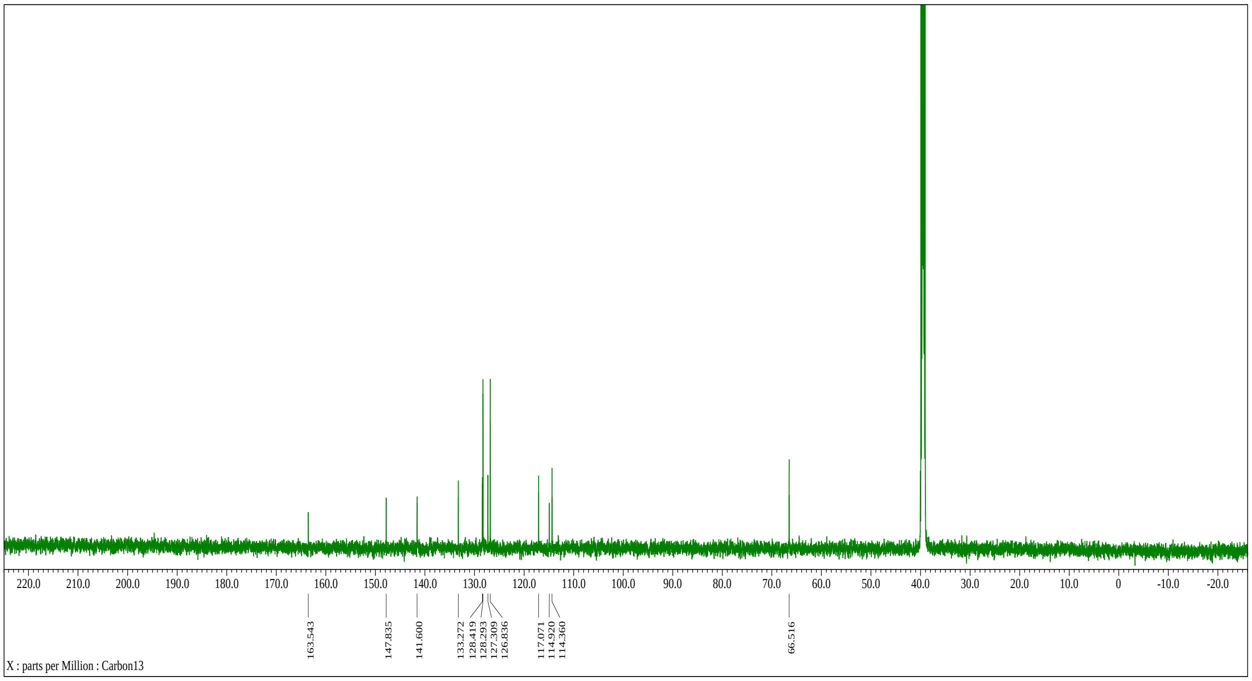

220.0 210.0 200.0 190.0 180.0 170.0 160.0 150.0 140.0 130.0 120.0 110.0 100.0 90.0 80.0 70.0 60.0 50.0 40.0 30.0 20.0 10.0 0 -10.0 -20.0
163.543
147.835
141.600
133.272 128.419 128.293 127.309 126.836
117.071 114.920 114.360
66.516
X : parts per Million : Carbon13

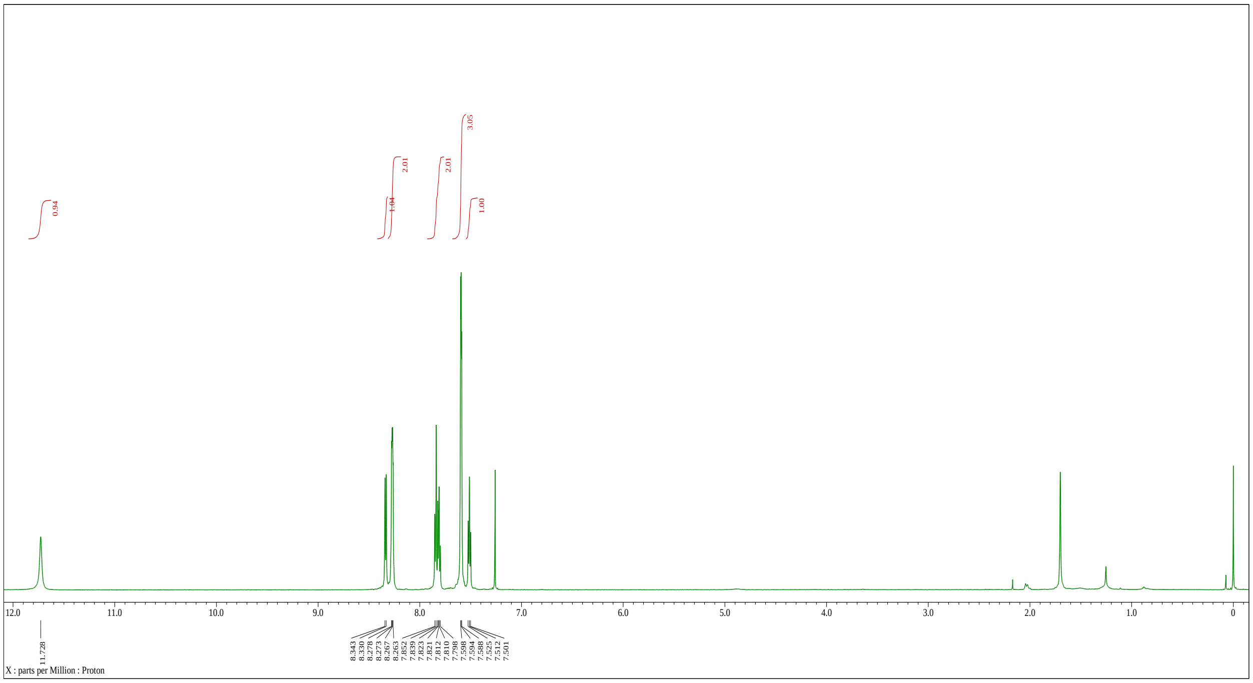
0.94
1.04
2.01
2.01
3.05
1.00
11.728
8.343
8.330
8.278
8.273
8.267
8.263
7.852
7.839
7.823
7.821
7.812
7.810
7.798
7.598
7.594
7.588
7.525
7.512
7.501
X : parts per Million : Proton

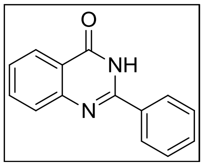

4a

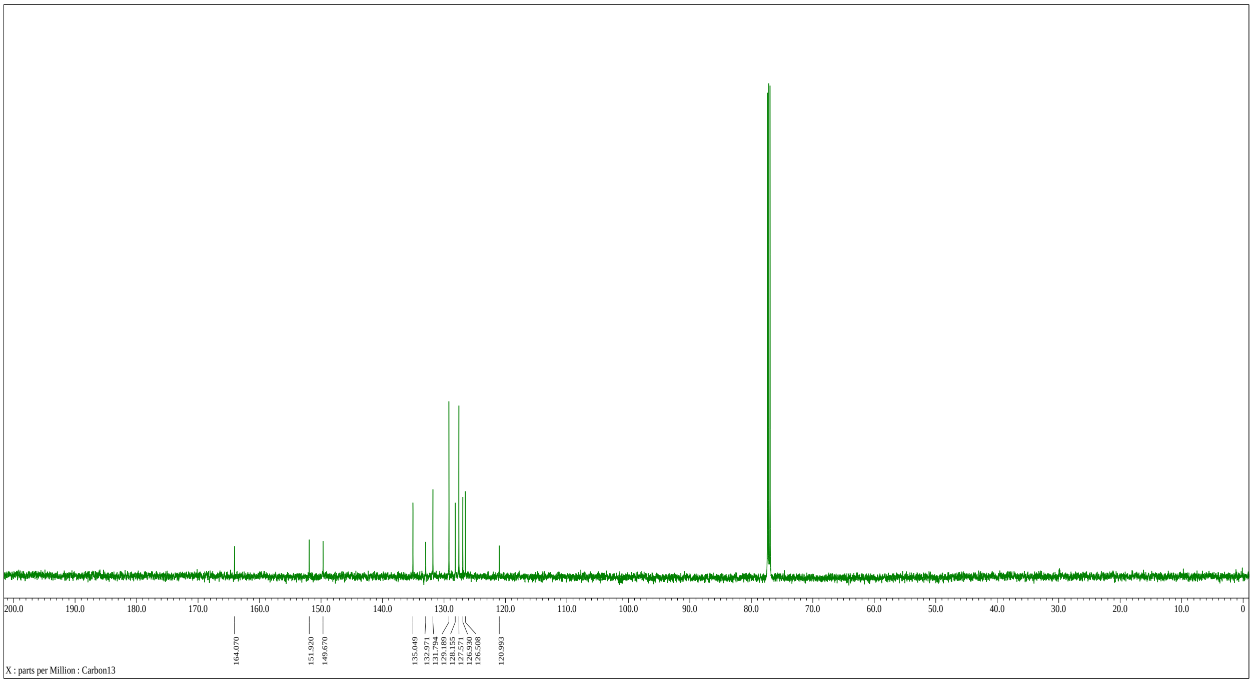
164.070
151.920
149.670
135.049
132.971
131.794
129.189
128.155
127.571
126.930
126.508
120.993
X : parts per Million : Carbon13

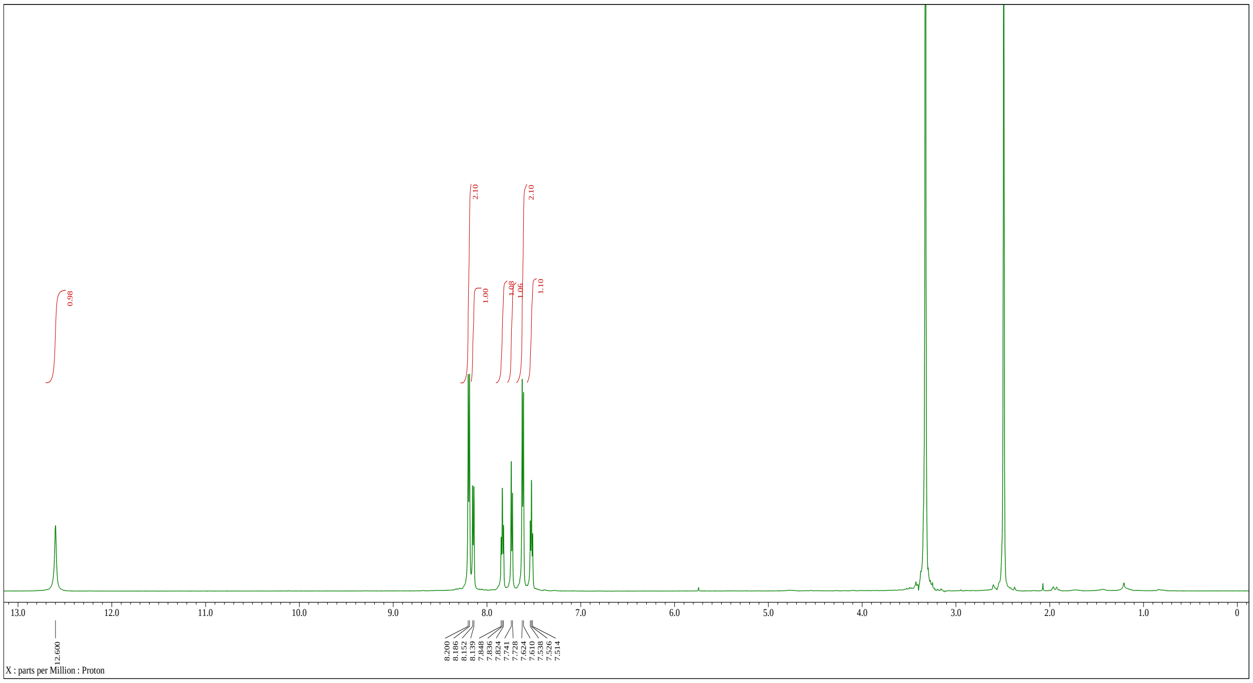

0.98
2.10
1.00
1.08
1.86
2.10
1.10
12.600
8.200
8.186
8.152
8.139
7.848
7.836
7.824
7.741
7.728
7.624
7.610
7.538
7.526
7.514
X : parts per Million : Proton

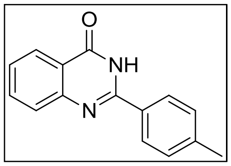

O
NH
N

4b

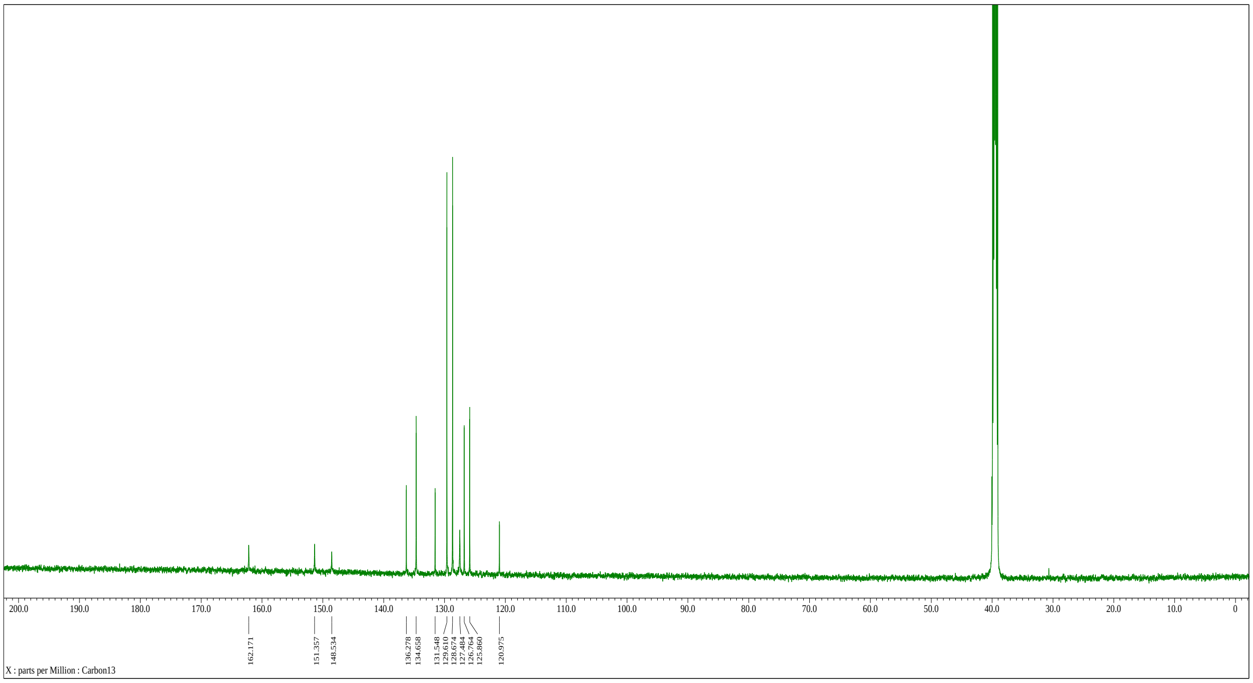

162.171
151.357
148.534
136.278
134.658
131.548
129.610
128.674
127.484
126.764
125.860
120.975
X : parts per Million : Carbon13

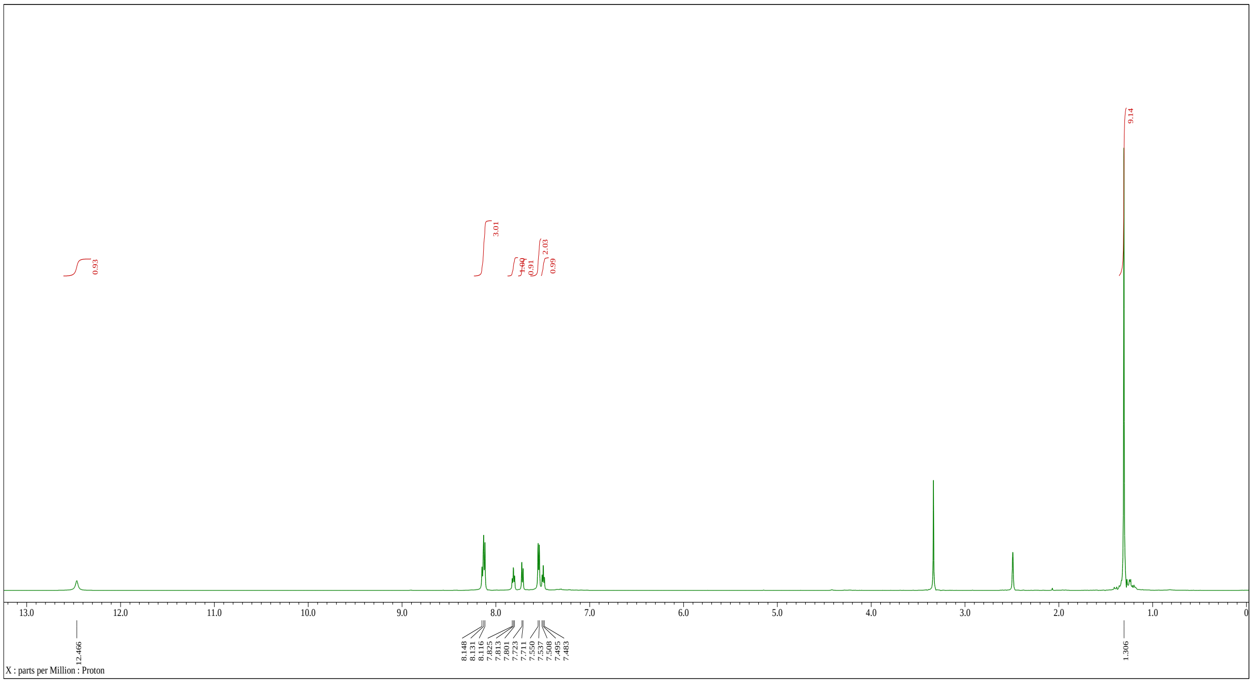
0.93
3.01
1.00
0.91
2.03
0.99
9.14
13.0 12.0 11.0 10.0 9.0 8.0 7.0 6.0 5.0 4.0 3.0 2.0 1.0 0
12.466
8.148
8.131
8.116
7.825
7.813
7.801
7.723
7.711
7.550
7.537
7.508
7.495
7.483
1.306
X : parts per Million : Proton

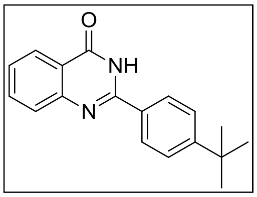
O
NH
N

4c

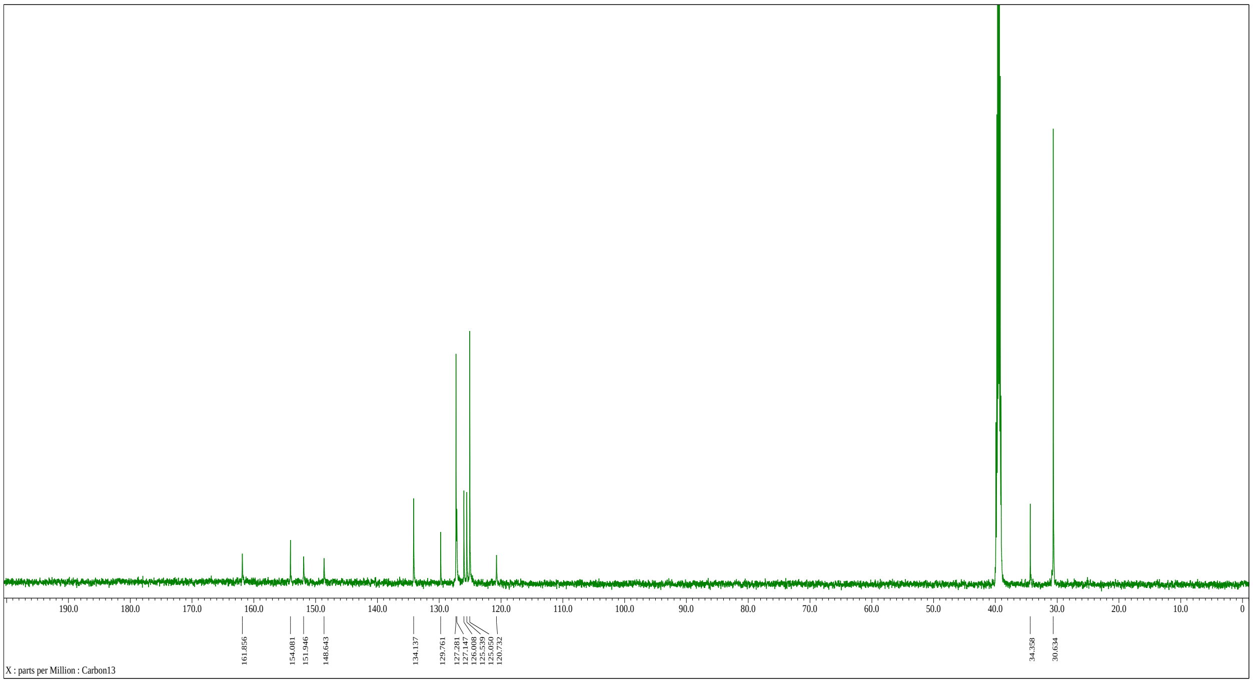
190.0 180.0 170.0 160.0 150.0 140.0 130.0 120.0 110.0 100.0 90.0 80.0 70.0 60.0 50.0 40.0 30.0 20.0 10.0 0
161.856
154.081
151.946
148.643
134.137
129.761
127.281
127.147
126.008
125.539
125.050
120.732
34.358
30.634
X : parts per Million : Carbon13

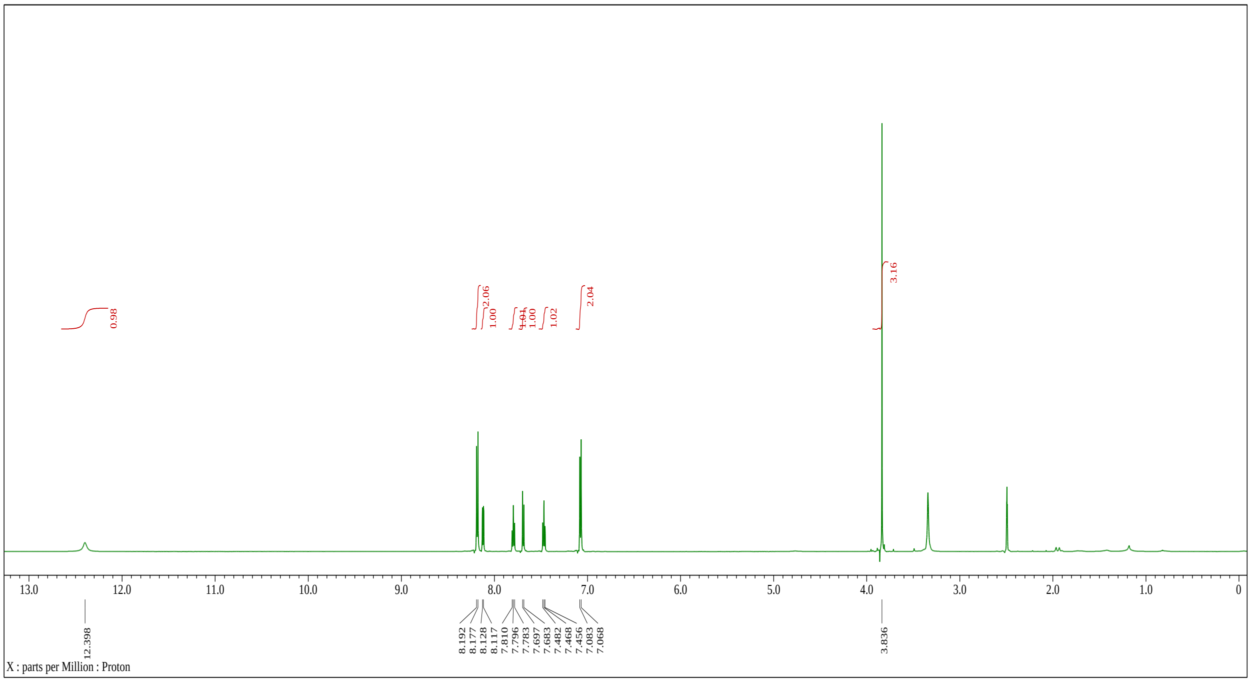
0.98
2.06
1.00
1.01
1.00
1.02
2.04
3.16
13.0
12.0
11.0
10.0
9.0
8.0
7.0
6.0
5.0
4.0
3.0
2.0
1.0
0
12.398
8.192
8.177
8.128
8.117
7.810
7.796
7.783
7.697
7.683
7.482
7.468
7.456
7.083
7.068
3.836
X : parts per Million : Proton

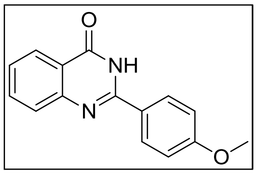
O
NH
N
O

4d

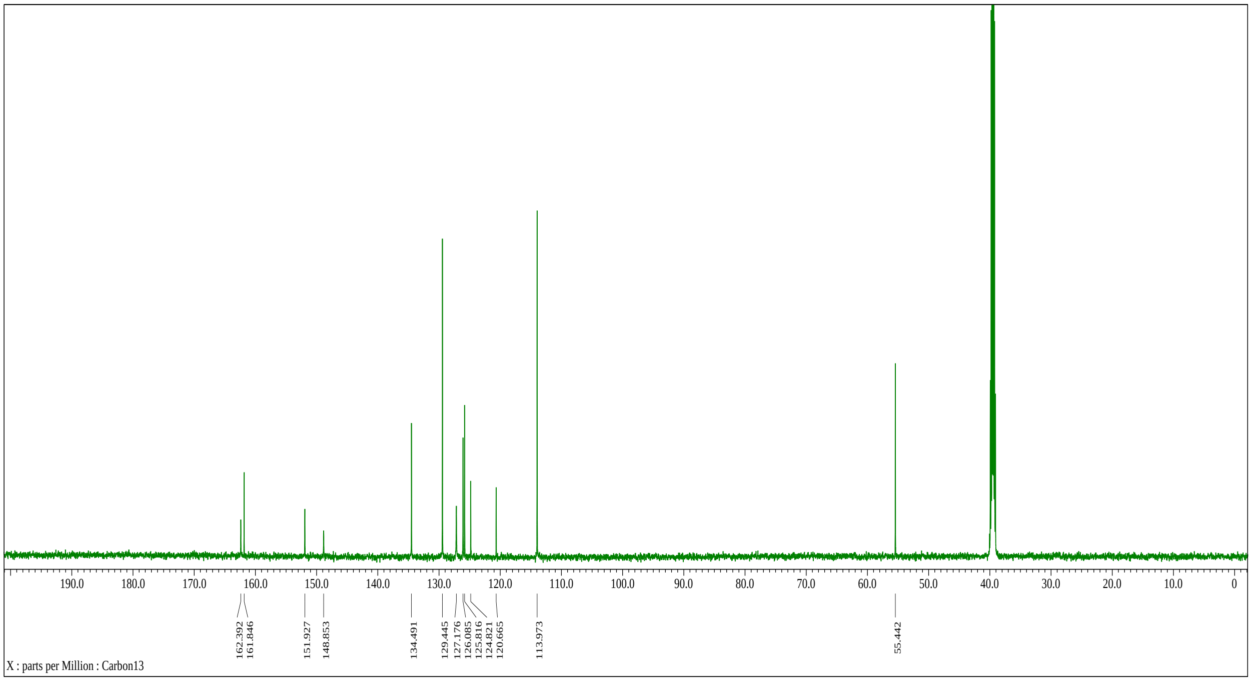
190.0
180.0
170.0
160.0
150.0
140.0
130.0
120.0
110.0
100.0
90.0
80.0
70.0
60.0
50.0
40.0
30.0
20.0
10.0
0
162.392
161.846
151.927
148.853
134.491
129.445
127.176
126.085
125.816
124.821
120.665
113.973
55.442
X : parts per Million : Carbon13

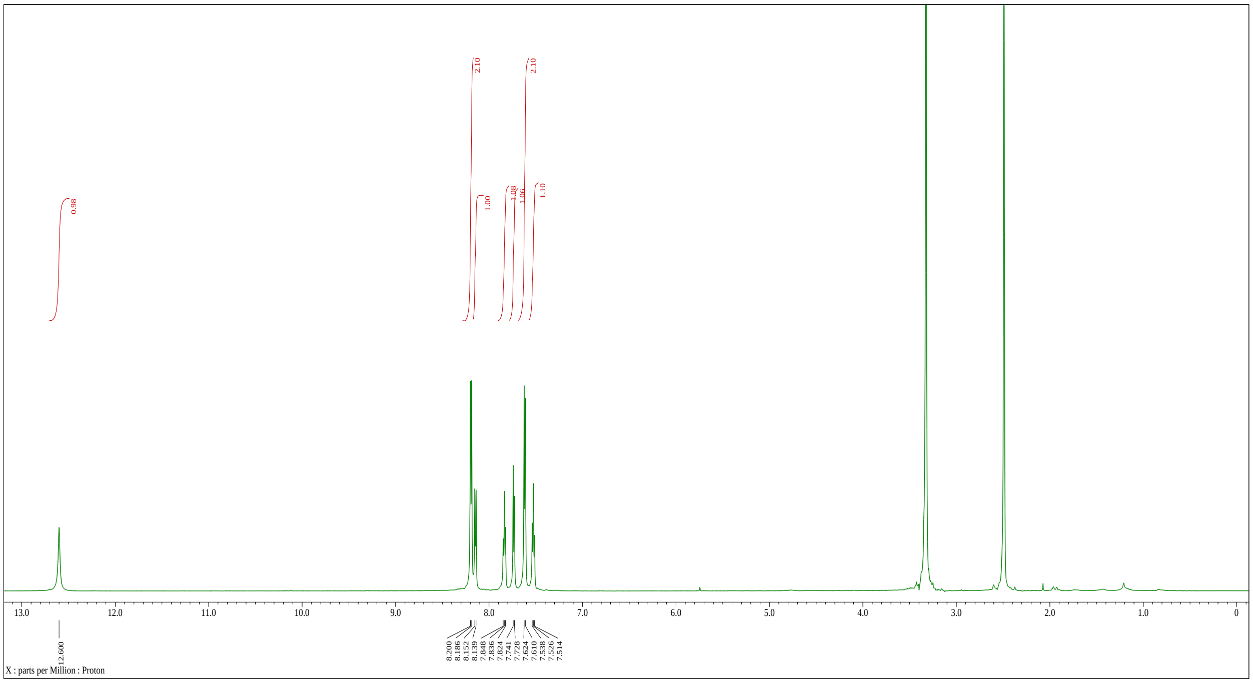

0.98
1.00
2.10
1.08
1.06
2.10
1.10
13.0
12.0
11.0
10.0
9.0
8.0
7.0
6.0
5.0
4.0
3.0
2.0
1.0
0
12.600
8.200
8.186
8.152
8.139
7.848
7.836
7.824
7.741
7.728
7.624
7.610
7.538
7.526
7.514
X : parts per Million : Proton

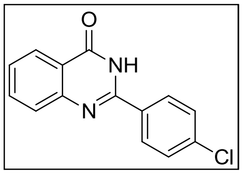

O
NH
N
Cl

4e

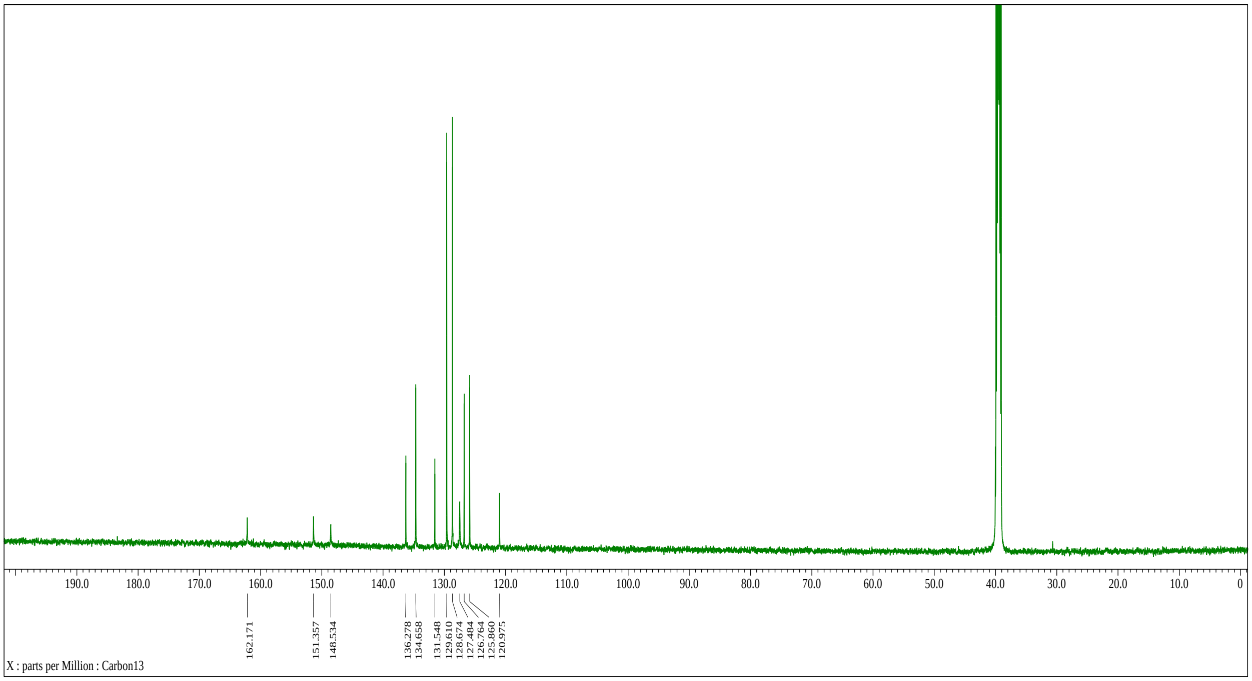

190.0
180.0
170.0
160.0
150.0
140.0
130.0
120.0
110.0
100.0
90.0
80.0
70.0
60.0
50.0
40.0
30.0
20.0
10.0
0
162.171
151.357
148.534
136.278
134.658
131.548
129.610
128.674
127.484
126.764
125.860
120.975
X : parts per Million : Carbon13

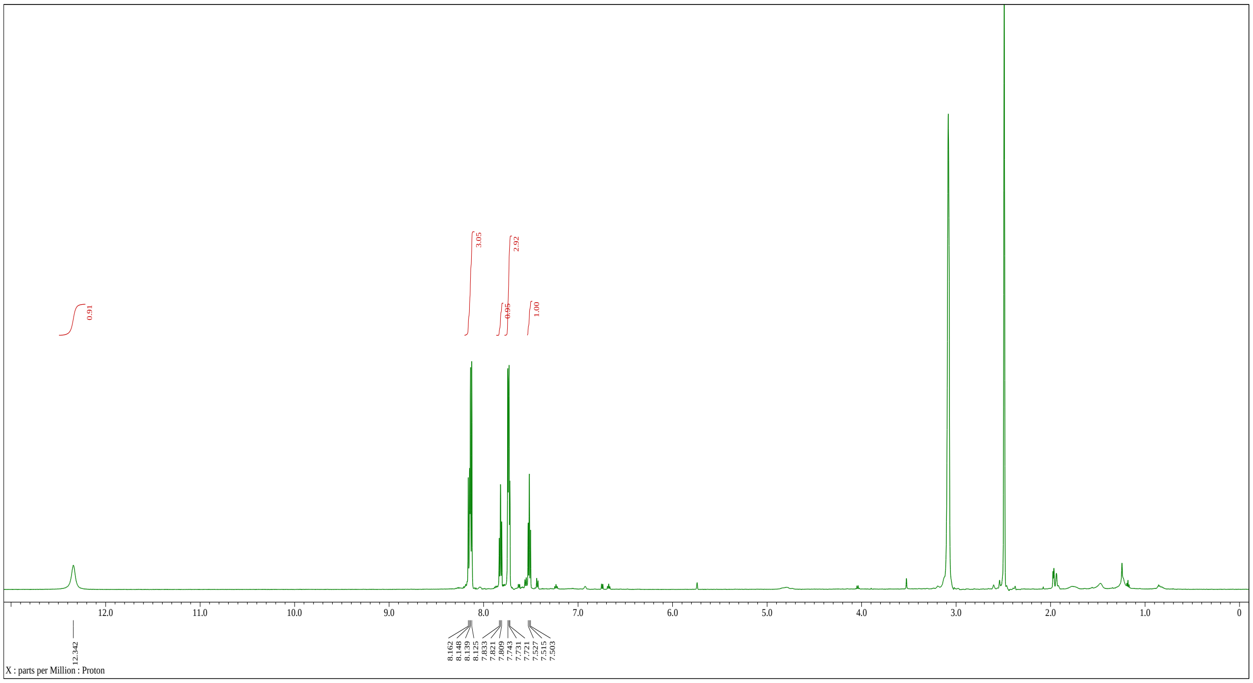
0.91
3.05
0.95
2.92
1.00
12.0 11.0 10.0 9.0 8.0 7.0 6.0 5.0 4.0 3.0 2.0 1.0 0
12.342
8.162 8.148 8.139 8.125 7.833 7.821 7.809 7.743 7.731 7.721 7.527 7.515 7.503
X : parts per Million : Proton

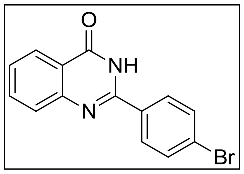
O
NH
N
Br

4f

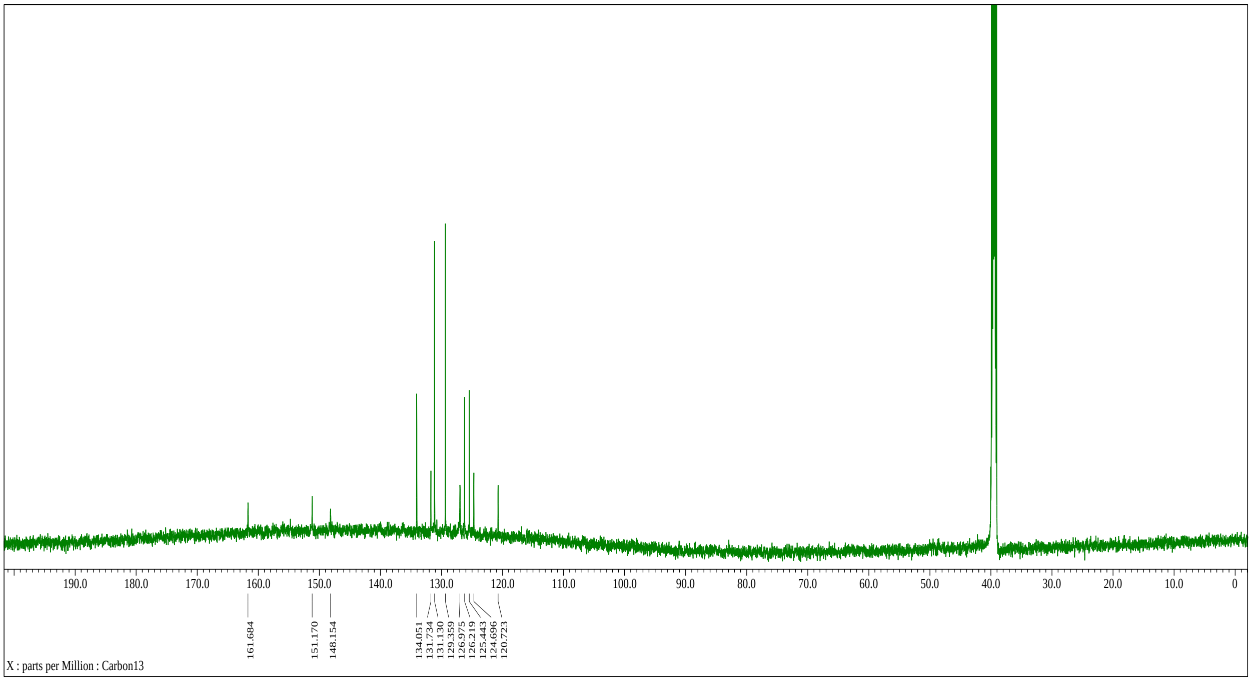
190.0 180.0 170.0 160.0 150.0 140.0 130.0 120.0 110.0 100.0 90.0 80.0 70.0 60.0 50.0 40.0 30.0 20.0 10.0 0
161.684
151.170
148.154
134.051 131.734 131.130 129.359 126.975 126.219 125.443 124.696 120.723
X : parts per Million : Carbon13

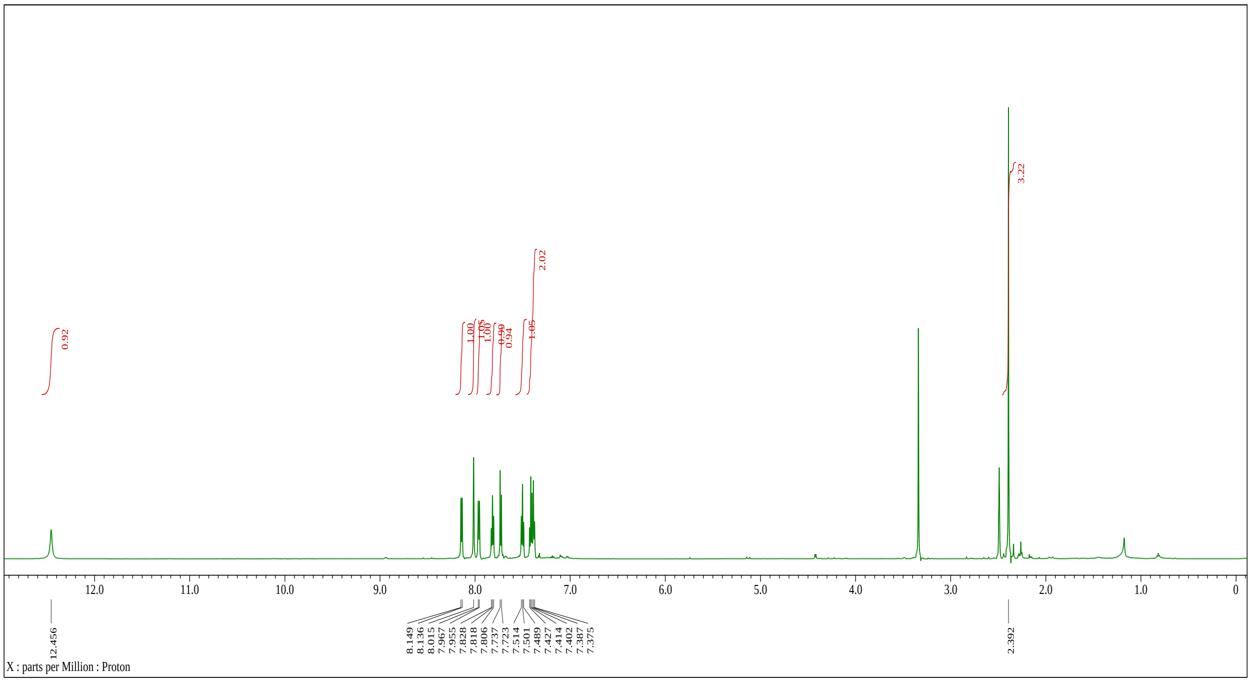

0.92
1.00
1.05
1.00
0.90
0.94
1.05
2.02
3.22
12.456
8.149
8.136
8.015
7.967
7.955
7.828
7.818
7.806
7.737
7.723
7.514
7.501
7.489
7.427
7.414
7.402
7.387
7.375
2.392
X : parts per Million : Proton

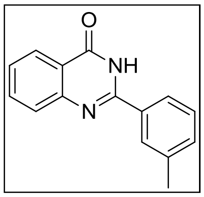

O
NH
N

4g

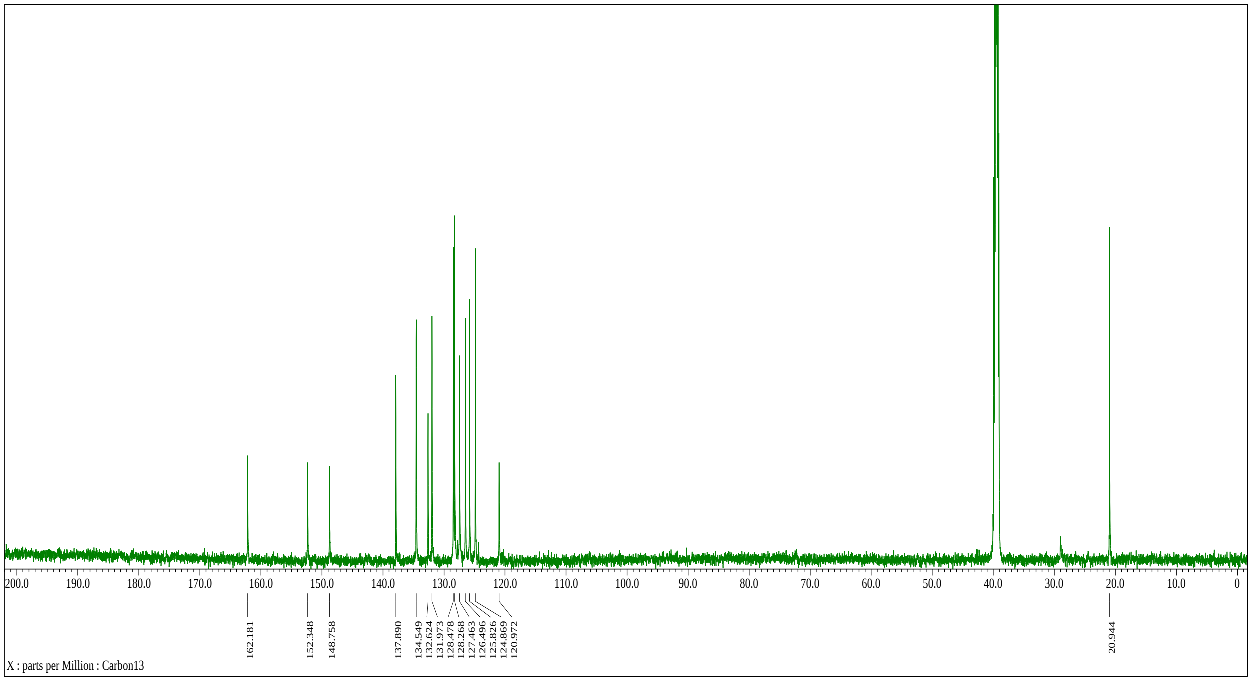

162.181
152.348
148.758
137.890
134.549
132.624
131.973
128.478
128.268
127.463
126.496
125.826
124.869
120.972
20.944
X : parts per Million : Carbon13

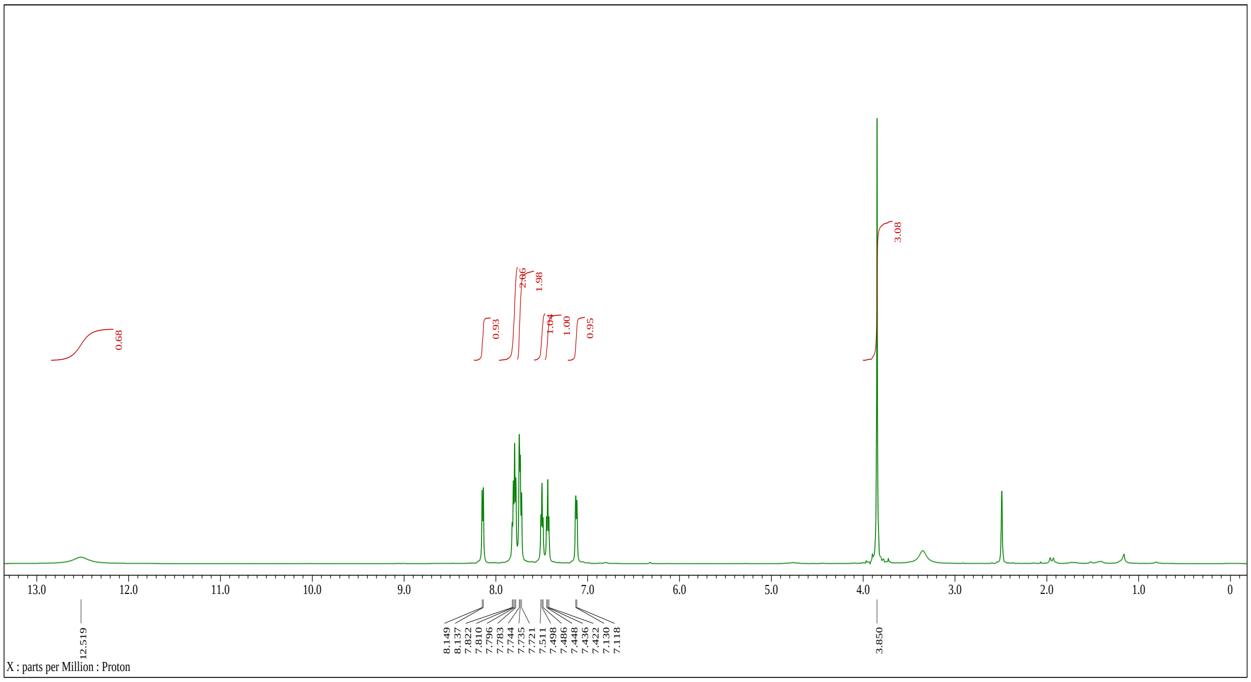
0.68
0.93
2.06
1.98
1.04
1.00
0.95
3.08
13.0 12.0 11.0 10.0 9.0 8.0 7.0 6.0 5.0 4.0 3.0 2.0 1.0 0
12.519
8.149 8.137 7.822 7.810 7.796 7.783 7.744 7.735 7.721 7.511 7.498 7.486 7.448 7.436 7.422 7.130 7.118
3.850
X : parts per Million : Proton

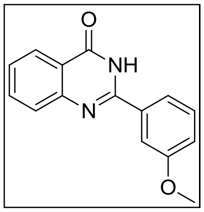
O
NH
N
O

4h

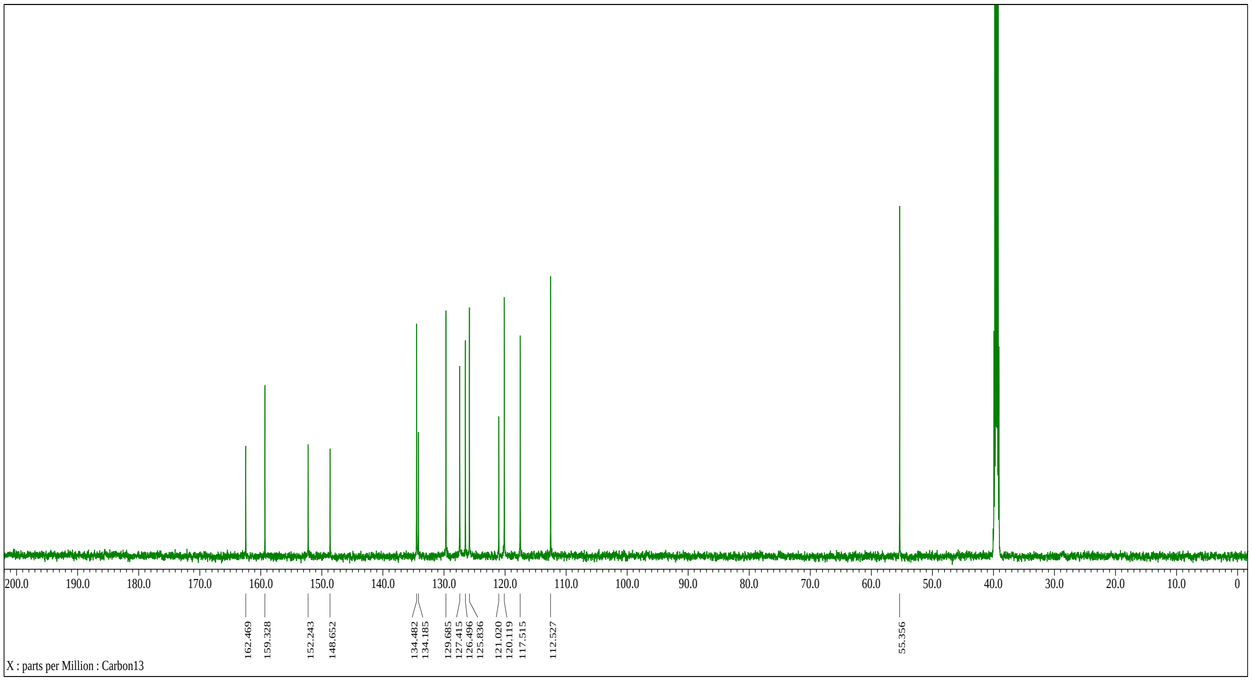
200.0 190.0 180.0 170.0 160.0 150.0 140.0 130.0 120.0 110.0 100.0 90.0 80.0 70.0 60.0 50.0 40.0 30.0 20.0 10.0 0
162.469 159.328 152.243 148.652 134.482 134.185 129.685 127.415 126.496 125.836 121.020 120.119 117.515 112.527 55.356
X : parts per Million : Carbon13

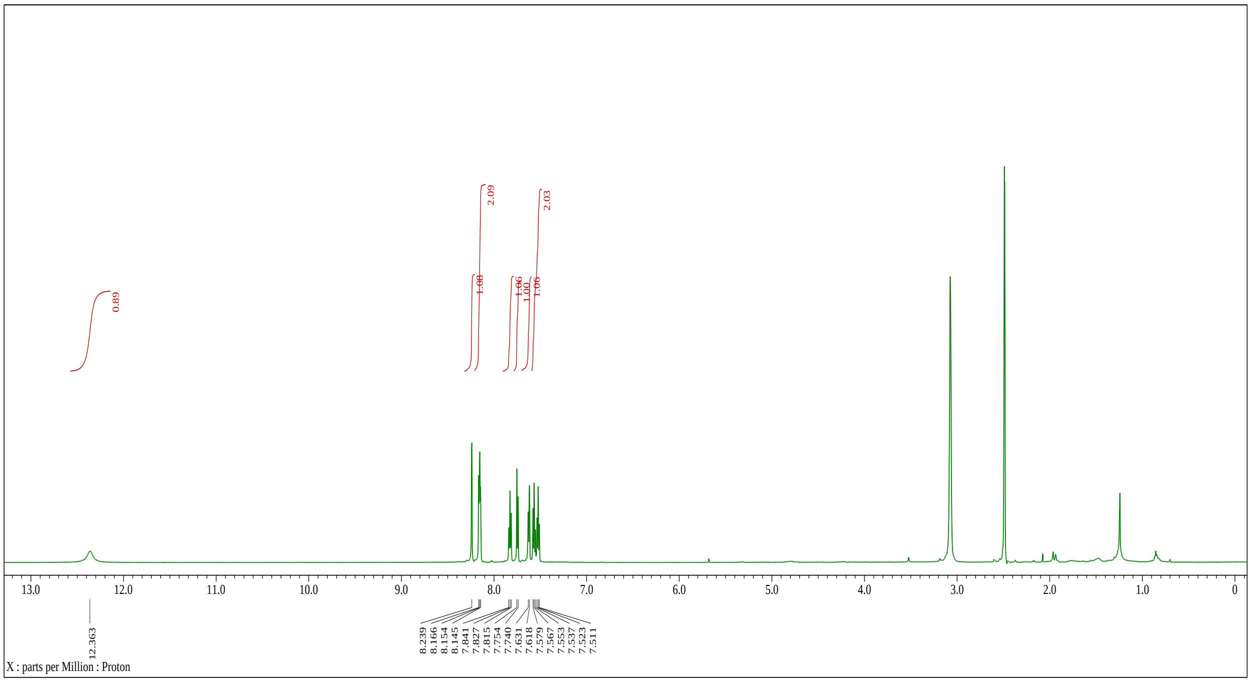
2.09
2.03
0.89
1.08
1.06
1.00
1.06
13.0
12.0
11.0
10.0
9.0
8.0
7.0
6.0
5.0
4.0
3.0
2.0
1.0
0
12.363
8.239
8.166
8.154
8.145
7.841
7.827
7.815
7.754
7.740
7.631
7.618
7.579
7.567
7.553
7.537
7.523
7.511
X : parts per Million : Proton

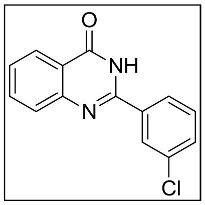
O
NH
N
Cl

4i

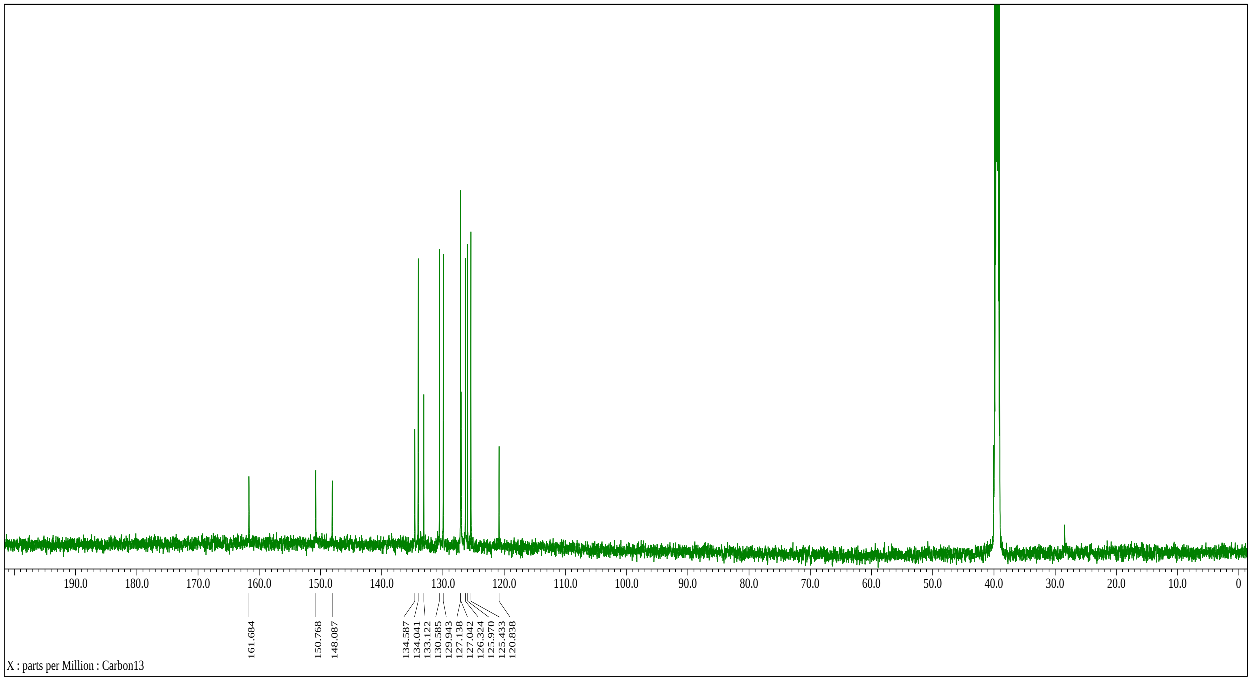
190.0
180.0
170.0
160.0
150.0
140.0
130.0
120.0
110.0
100.0
90.0
80.0
70.0
60.0
50.0
40.0
30.0
20.0
10.0
0
161.684
150.768
148.087
134.587
134.041
133.122
130.585
129.943
127.138
127.042
126.324
125.970
125.433
120.838
X : parts per Million : Carbon13

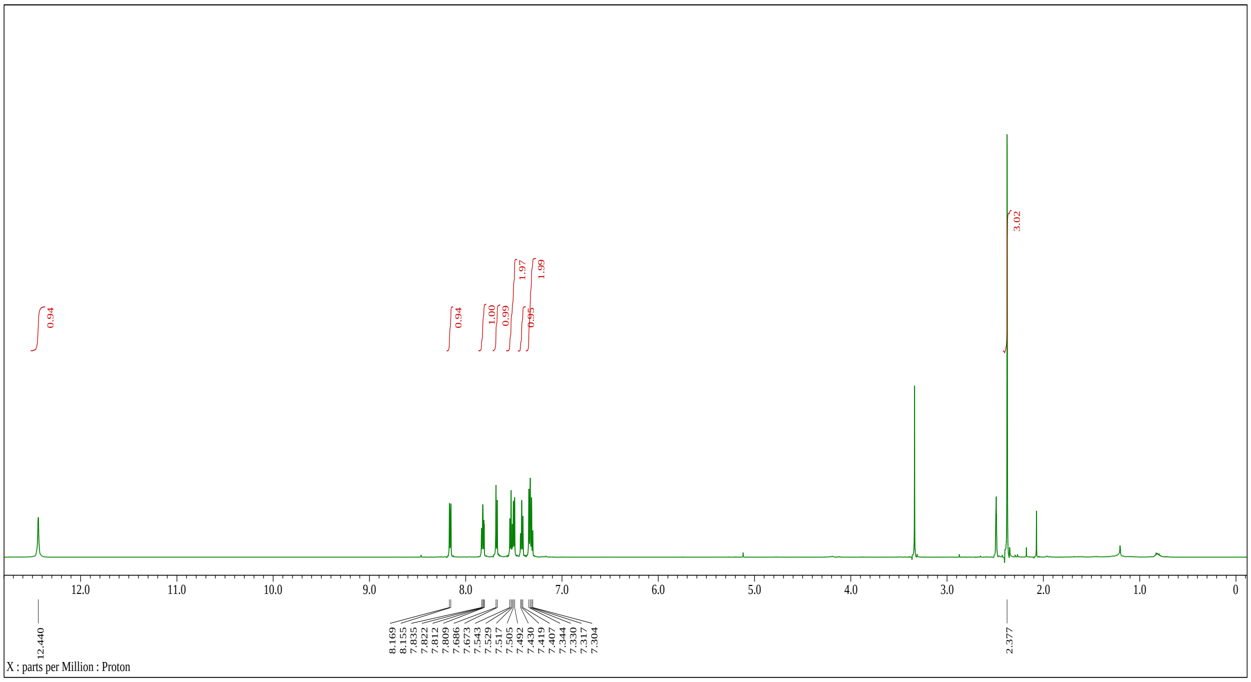
0.94
0.94
1.00
0.99
1.97
0.95
1.99
3.02
12.0
11.0
10.0
9.0
8.0
7.0
6.0
5.0
4.0
3.0
2.0
1.0
0
12.440
8.169
8.155
7.835
7.822
7.812
7.809
7.686
7.673
7.543
7.529
7.517
7.505
7.492
7.430
7.419
7.407
7.344
7.330
7.317
7.304
2.377
X : parts per Million : Proton

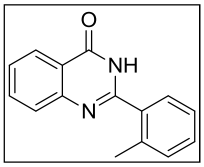
O
NH
N

4j

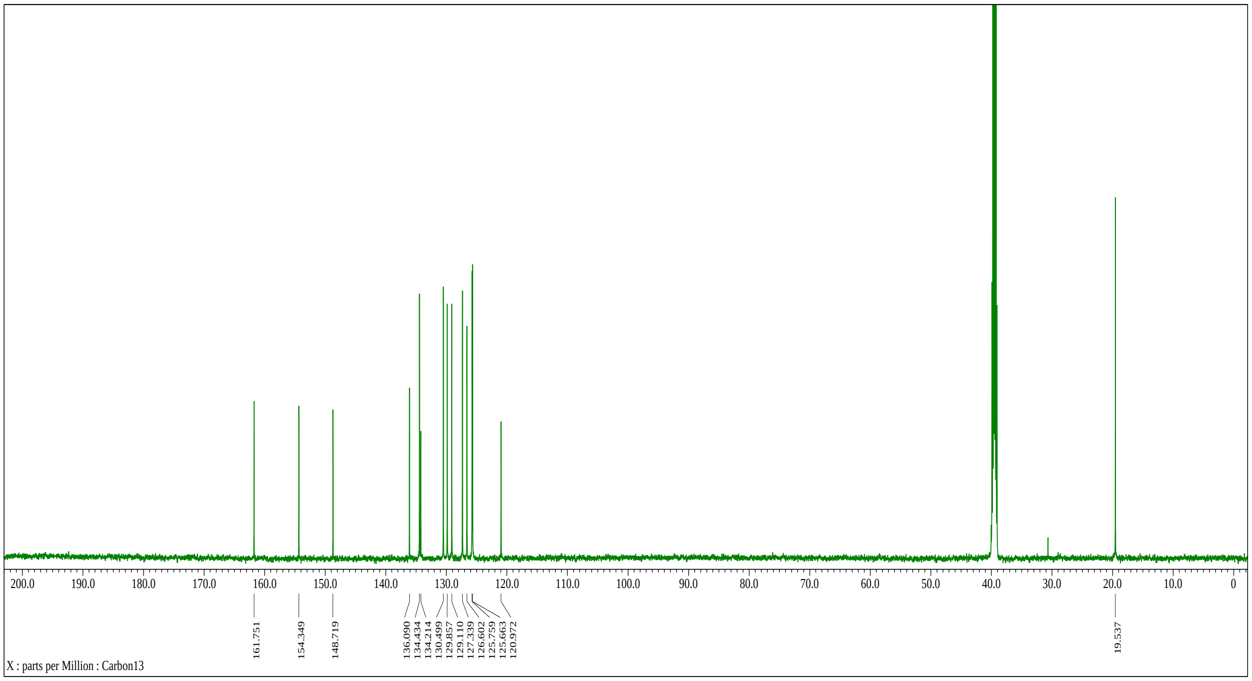
200.0
190.0
180.0
170.0
160.0
150.0
140.0
130.0
120.0
110.0
100.0
90.0
80.0
70.0
60.0
50.0
40.0
30.0
20.0
10.0
0
161.751
154.349
148.719
136.090
134.434
134.214
130.499
129.857
129.110
127.339
126.602
125.759
125.663
120.972
19.537
X : parts per Million : Carbon13

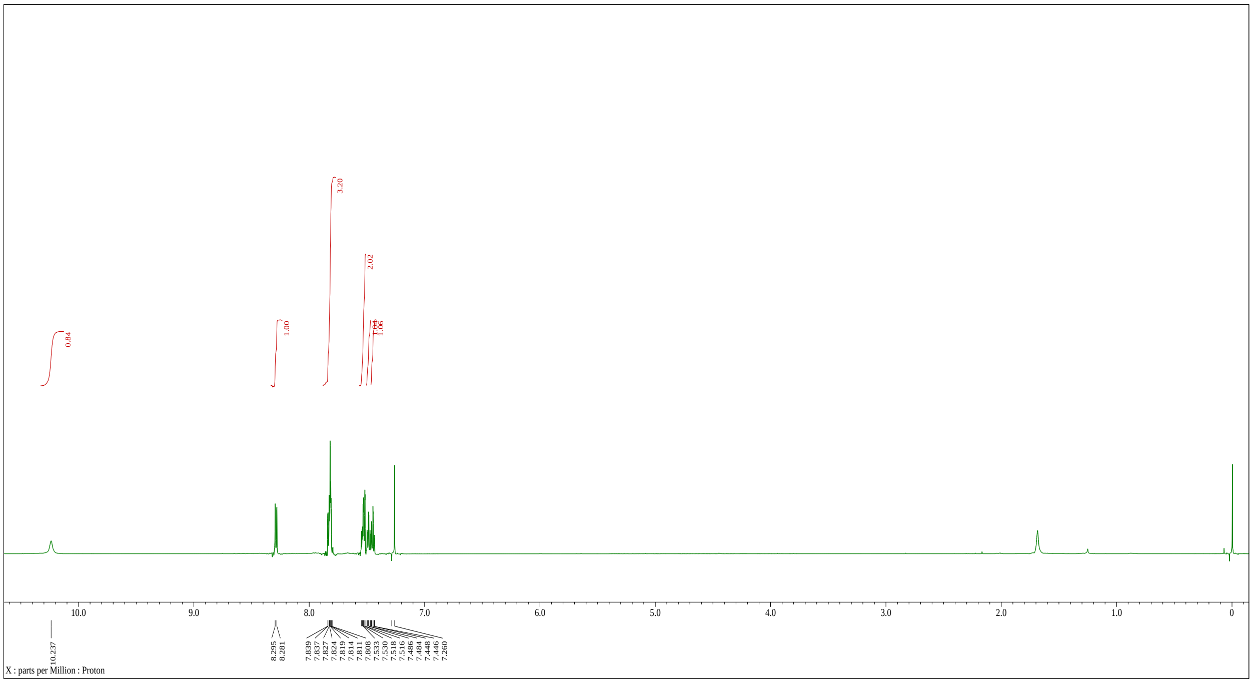
0.84
1.00
3.20
2.02
10.237
8.295
8.281
7.839
7.837
7.827
7.824
7.819
7.814
7.811
7.808
7.533
7.530
7.518
7.516
7.486
7.484
7.448
7.446
7.260
X : parts per Million : Proton

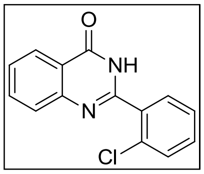
O
NH
N
Cl

4k

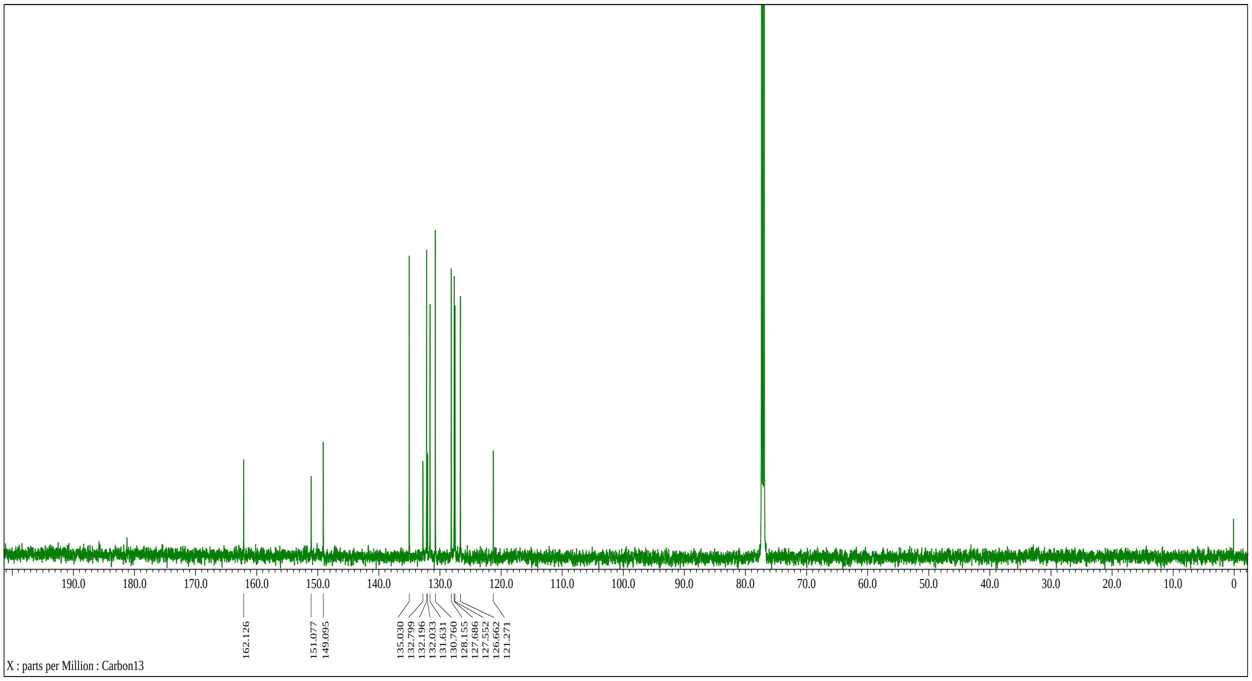
162.126
151.077
149.095
135.030
132.799
132.196
132.033
131.631
130.760
128.155
127.686
127.552
126.662
121.271
X : parts per Million : Carbon13

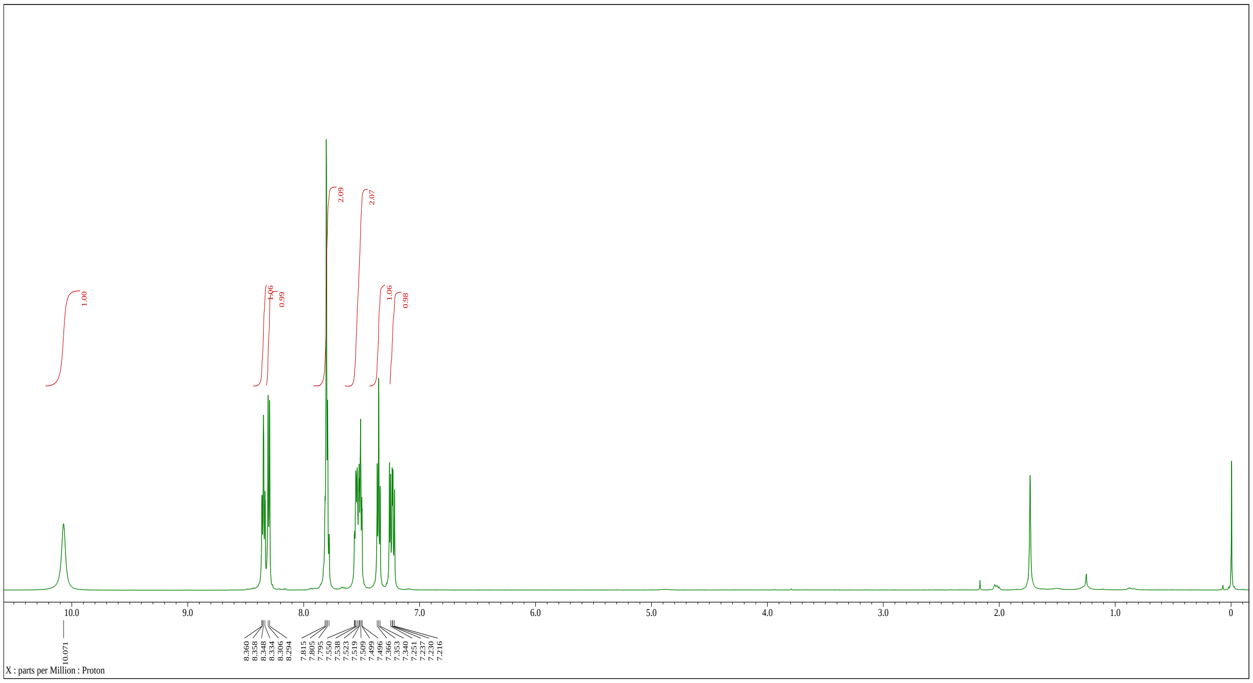
1.00
1.06
0.99
2.09
2.07
1.06
0.98
10.0
9.0
8.0
7.0
6.0
5.0
4.0
3.0
2.0
1.0
0
10.071
8.360
8.358
8.348
8.334
8.306
8.294
7.815
7.805
7.795
7.550
7.538
7.523
7.519
7.509
7.499
7.496
7.366
7.353
7.340
7.251
7.237
7.230
7.216
X : parts per Million : Proton

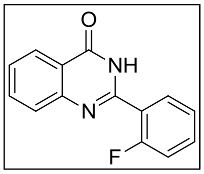
O
NH
N
F

4l

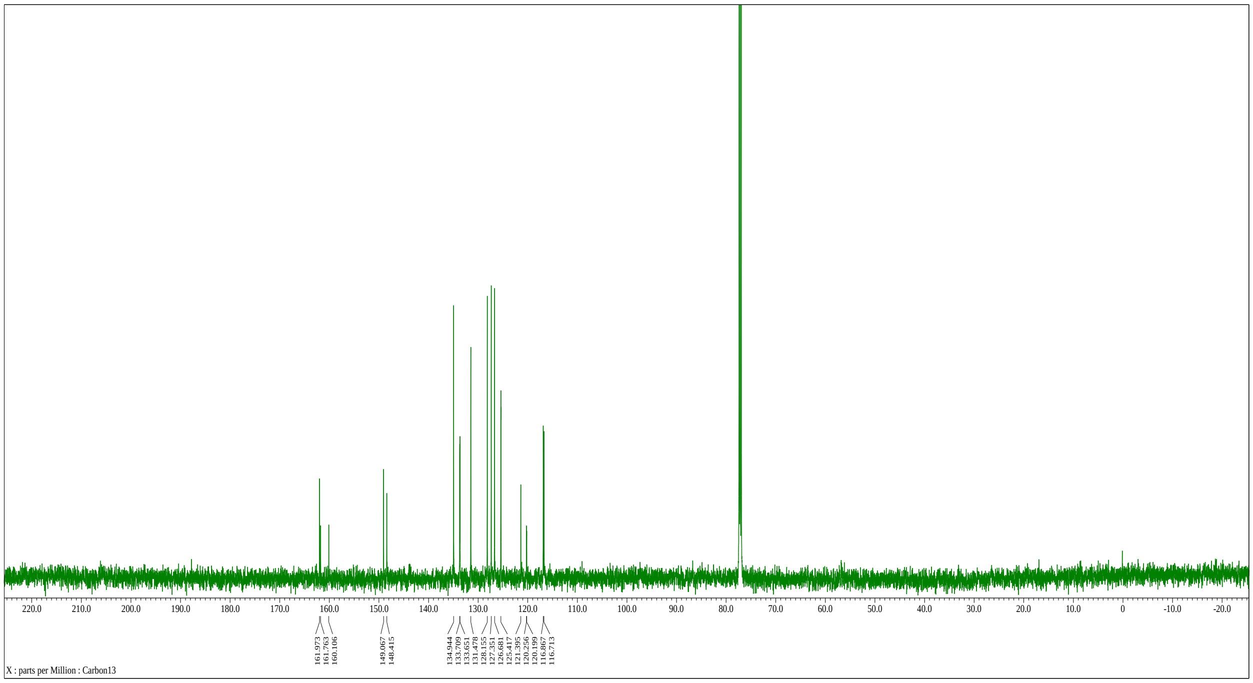
220.0
210.0
200.0
190.0
180.0
170.0
160.0
150.0
140.0
130.0
120.0
110.0
100.0
90.0
80.0
70.0
60.0
50.0
40.0
30.0
20.0
10.0
0
-10.0
-20.0
161.973
161.763
160.106
149.067
148.415
134.944
133.709
133.651
131.478
128.155
127.351
126.681
125.417
121.395
120.256
120.199
116.867
116.713
X : parts per Million : Carbon13

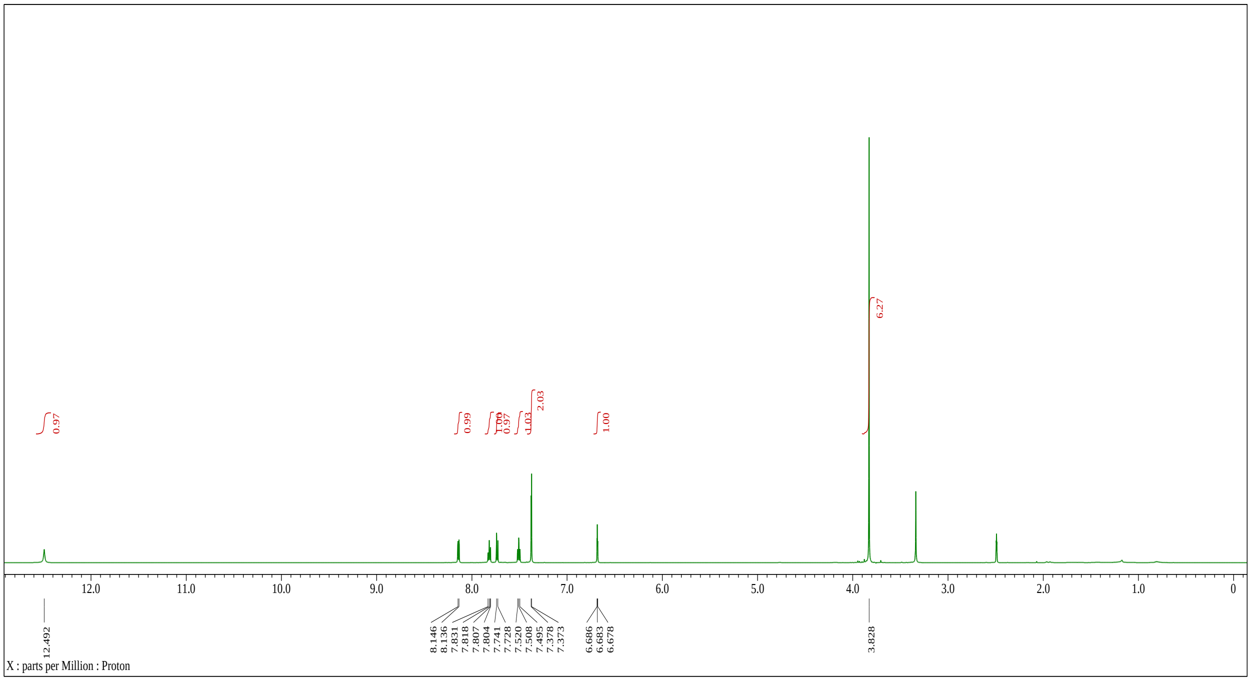
0.97
0.99
1.00
0.97
1.03
2.03
1.00
6.27
12.0
11.0
10.0
9.0
8.0
7.0
6.0
5.0
4.0
3.0
2.0
1.0
0
12.492
8.146
8.136
7.831
7.818
7.807
7.804
7.741
7.728
7.520
7.508
7.495
7.378
7.373
6.686
6.683
6.678
3.828
X : parts per Million : Proton

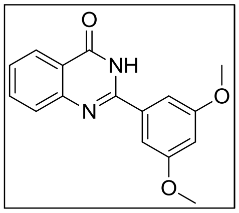
O
NH
N
O
O

4m

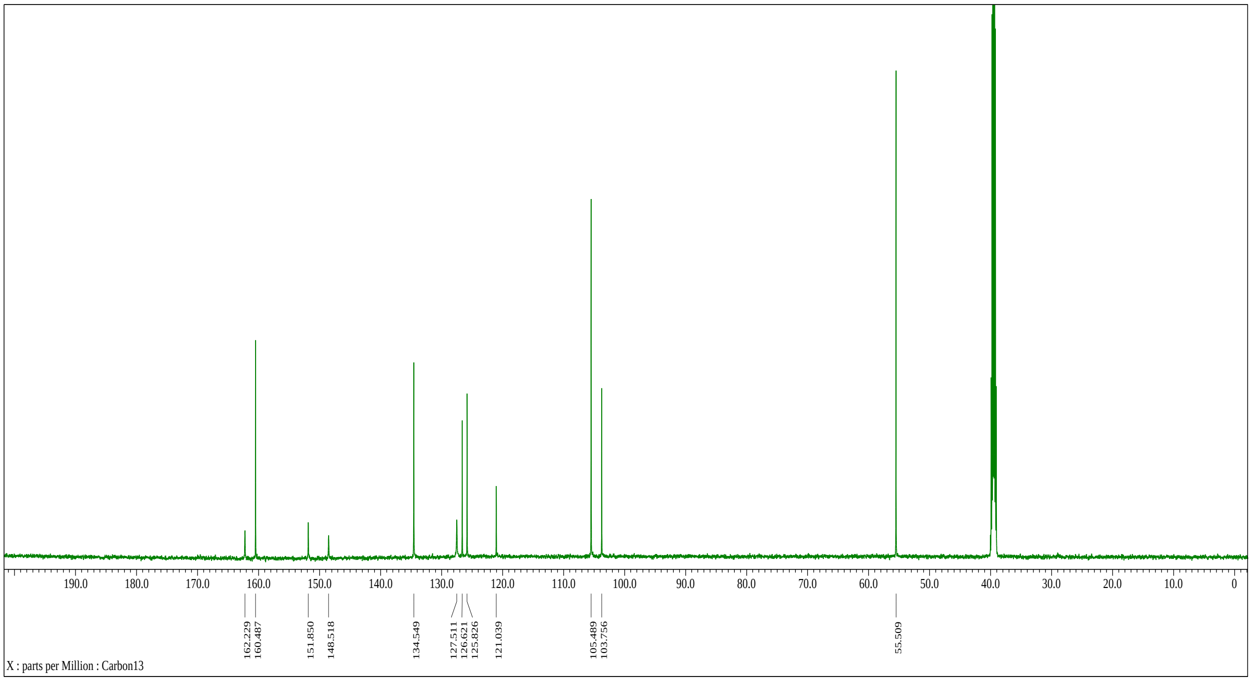
190.0
180.0
170.0
160.0
150.0
140.0
130.0
120.0
110.0
100.0
90.0
80.0
70.0
60.0
50.0
40.0
30.0
20.0
10.0
0
162.229
160.487
151.850
148.518
134.549
127.511
126.621
125.826
121.039
105.489
103.756
55.509
X : parts per Million : Carbon13

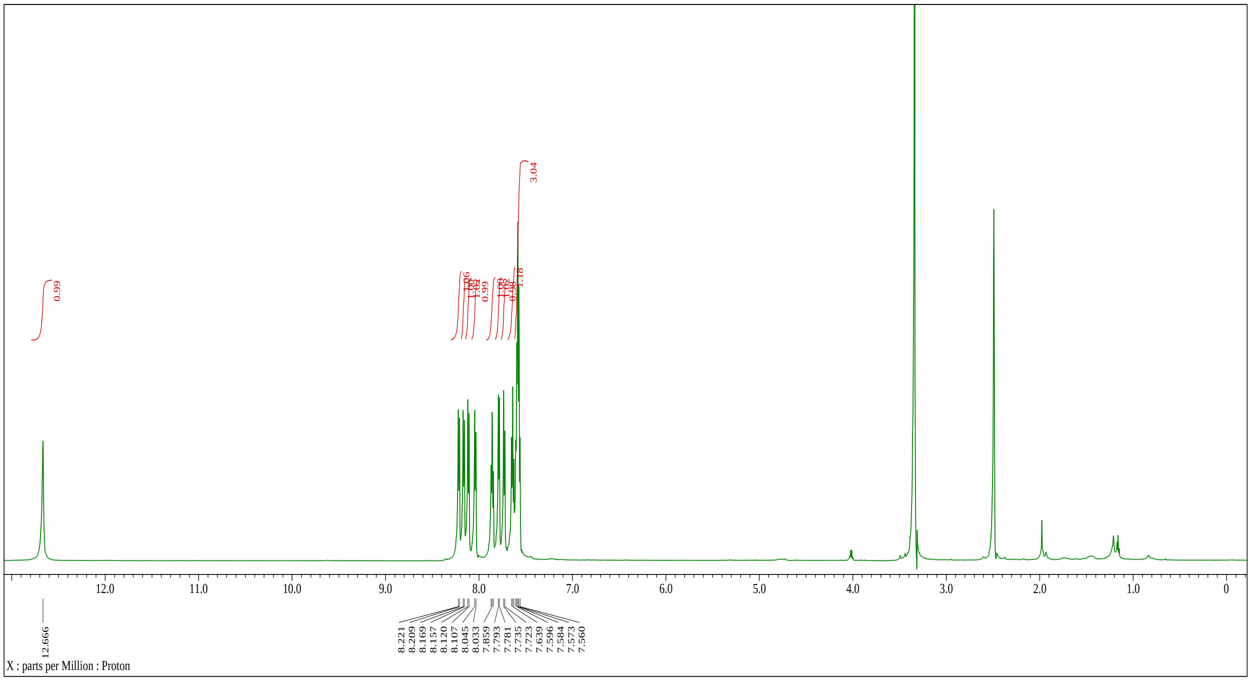
X : parts per Million : Proton

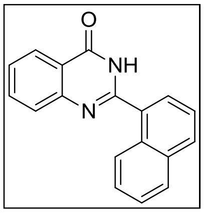
O
NH
N

4n

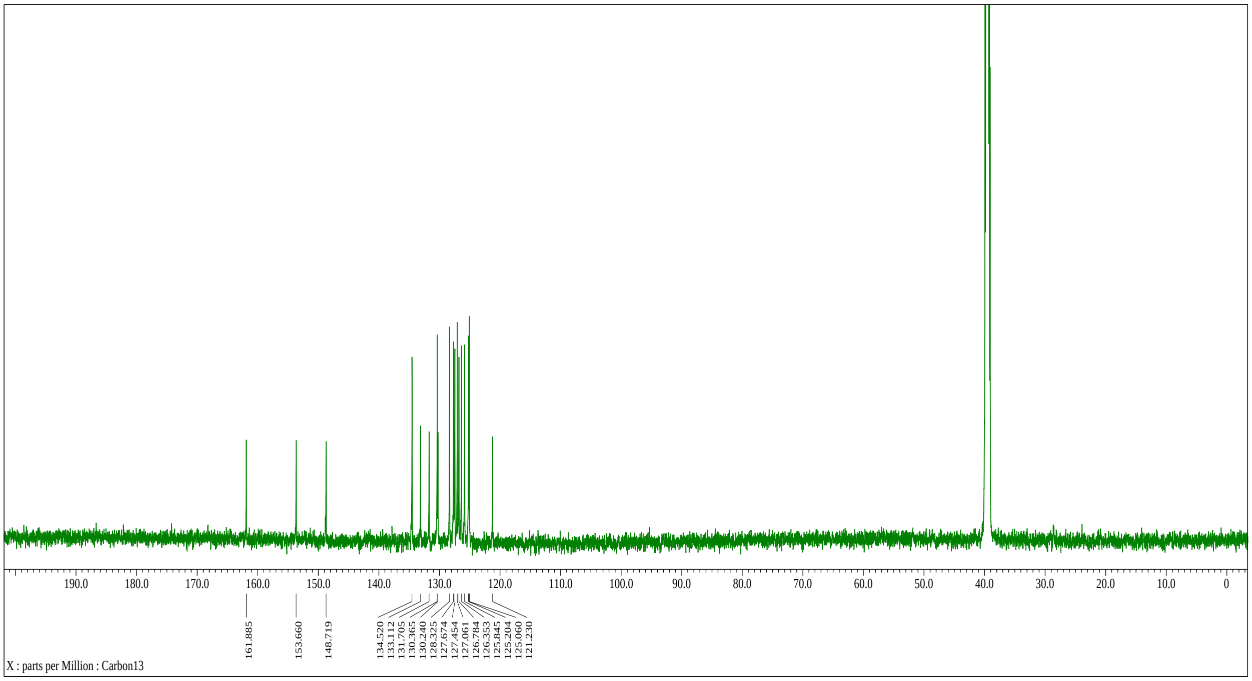
X : parts per Million : Carbon13

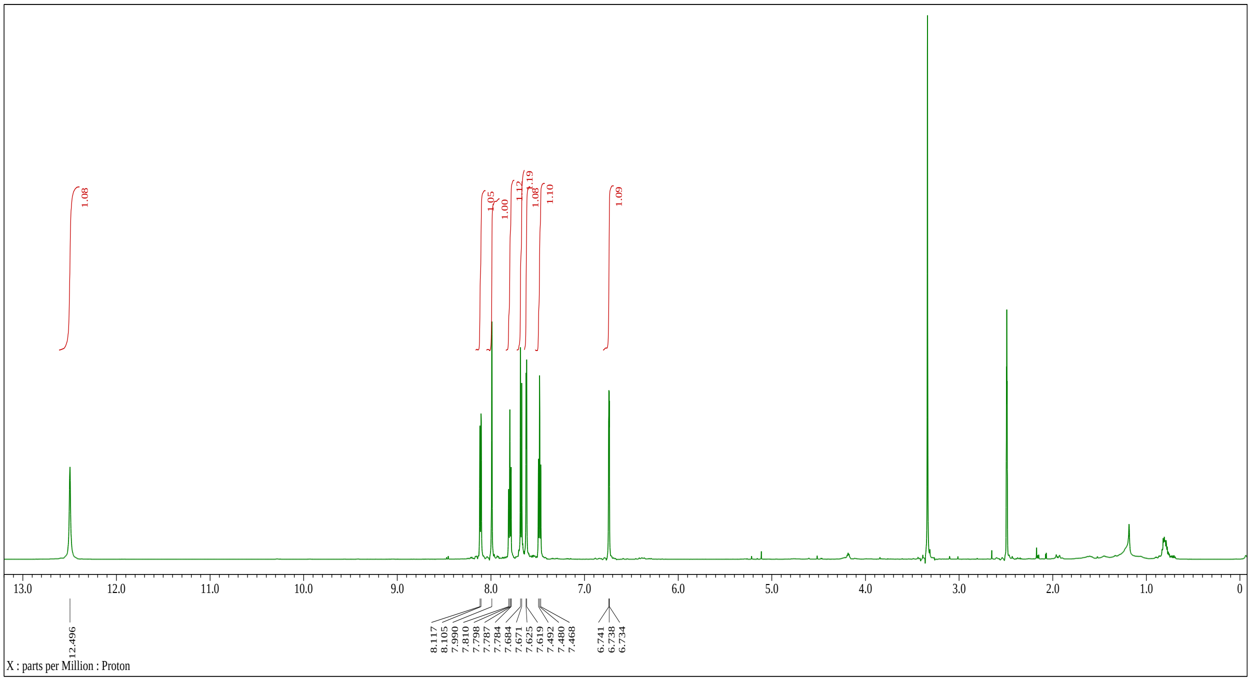
1.08
1.05
1.00
1.12
1.19
1.08
1.10
1.09
13.0
12.0
11.0
10.0
9.0
8.0
7.0
6.0
5.0
4.0
3.0
2.0
1.0
0
12.496
8.117
8.105
7.990
7.810
7.798
7.787
7.784
7.684
7.671
7.625
7.619
7.492
7.480
7.468
6.741
6.738
6.734
X : parts per Million : Proton

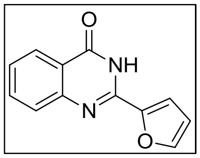
O
NH
N
O

4o

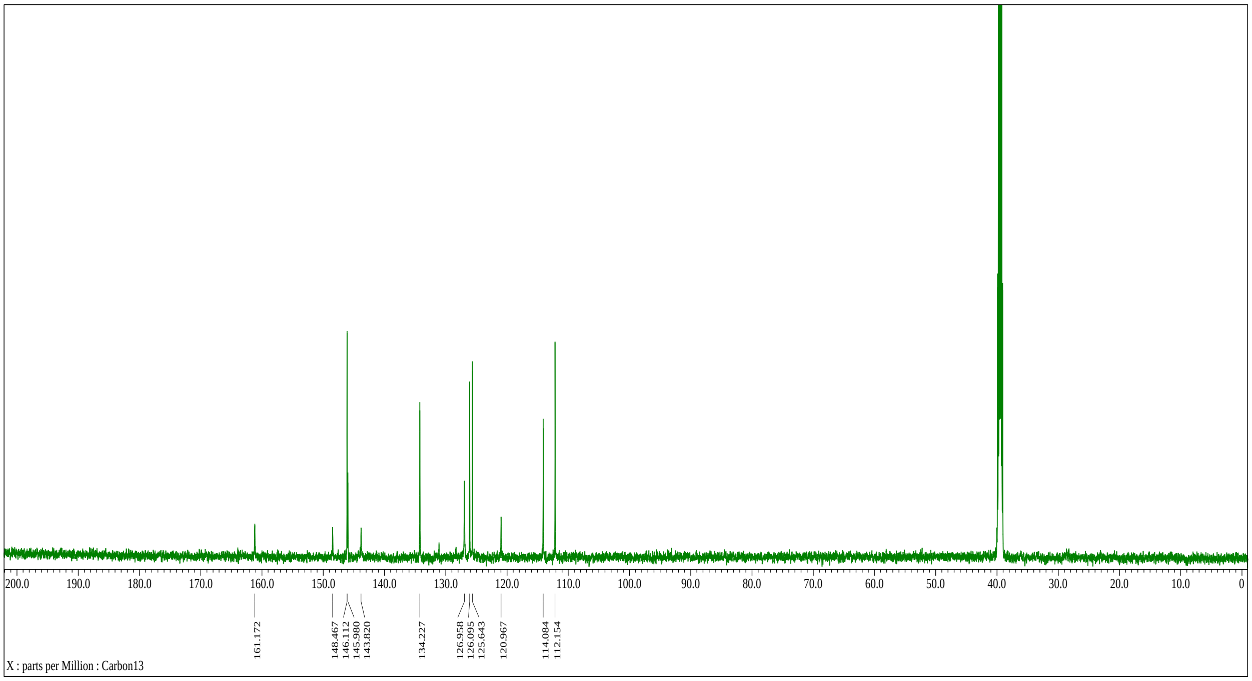
200.0
190.0
180.0
170.0
160.0
150.0
140.0
130.0
120.0
110.0
100.0
90.0
80.0
70.0
60.0
50.0
40.0
30.0
20.0
10.0
0
161.172
148.467
146.112
145.980
143.820
134.227
126.958
126.095
125.643
120.967
114.084
112.154
X : parts per Million : Carbon13

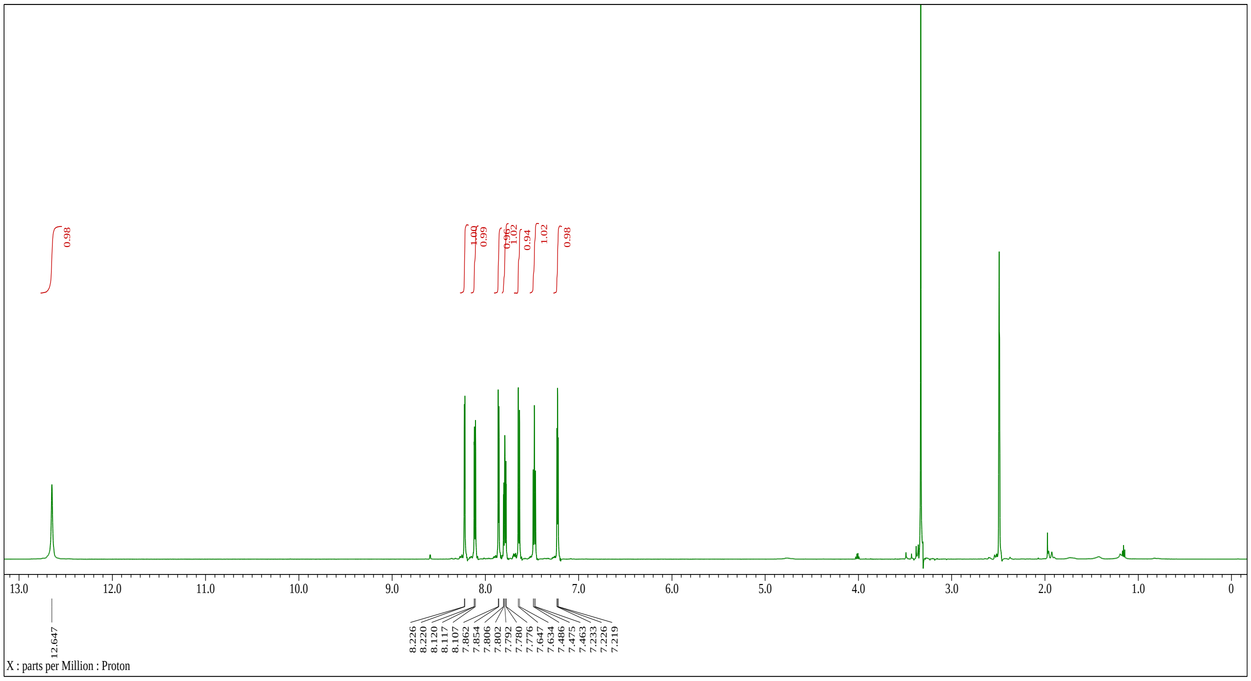
0.98
1.00
0.99
0.96
1.02
0.94
1.02
0.98
13.0 12.0 11.0 10.0 9.0 8.0 7.0 6.0 5.0 4.0 3.0 2.0 1.0 0
12.647
8.226 8.220 8.120 8.117 8.107 7.862 7.854 7.806 7.802 7.792 7.780 7.776 7.647 7.634 7.486 7.475 7.463 7.233 7.226 7.219
X : parts per Million : Proton

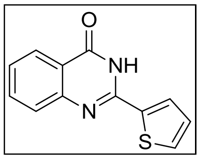
O
NH
N
S

4p

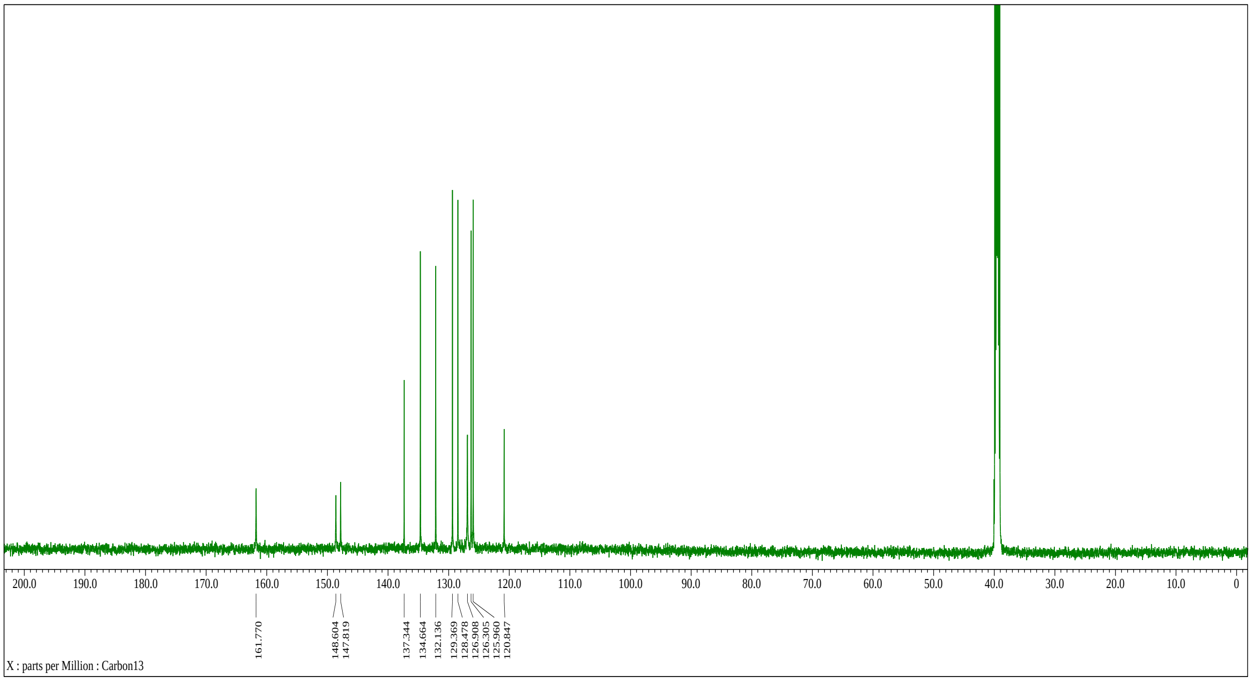
200.0 190.0 180.0 170.0 160.0 150.0 140.0 130.0 120.0 110.0 100.0 90.0 80.0 70.0 60.0 50.0 40.0 30.0 20.0 10.0 0
161.770
148.604 147.819
137.344 134.664 132.136 129.369 128.478 126.908 126.305 125.960 120.847
X : parts per Million : Carbon13

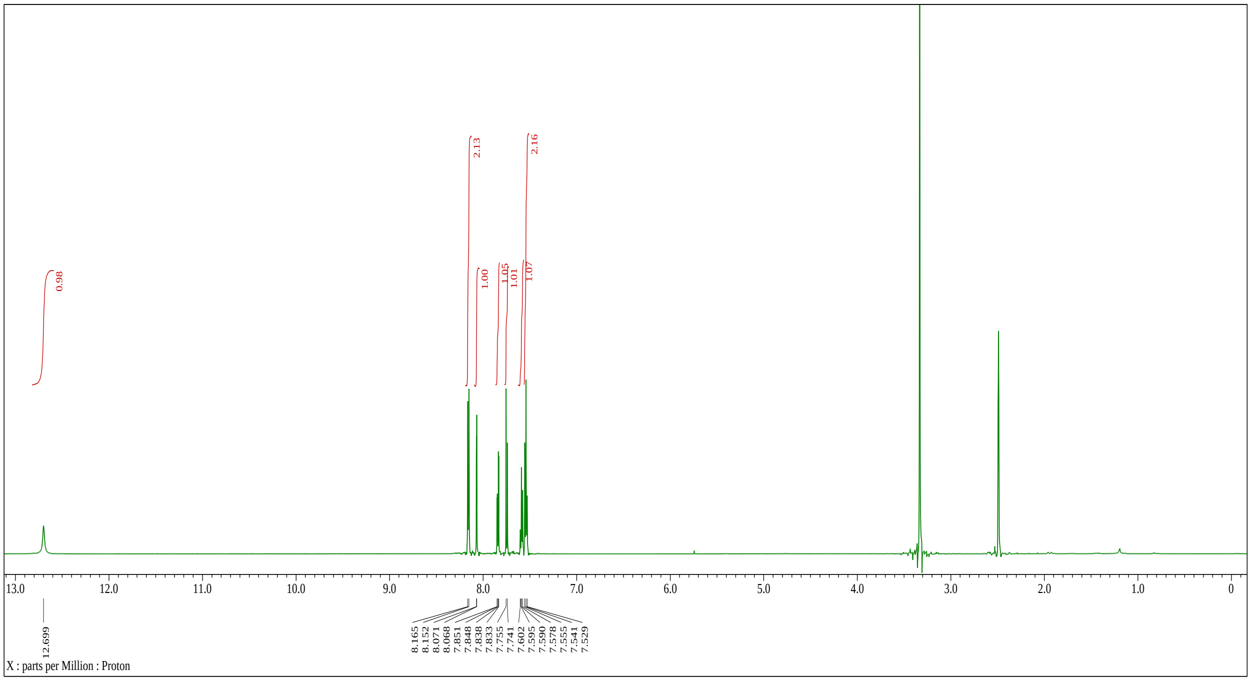
0.98
2.13
1.00
1.05
1.01
1.07
2.16
13.0
12.0
11.0
10.0
9.0
8.0
7.0
6.0
5.0
4.0
3.0
2.0
1.0
0
12.699
8.165
8.152
8.071
8.068
7.851
7.848
7.838
7.833
7.755
7.741
7.602
7.595
7.590
7.578
7.555
7.541
7.529
X : parts per Million : Proton

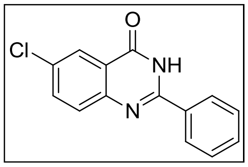
O
Cl
NH
N

4q

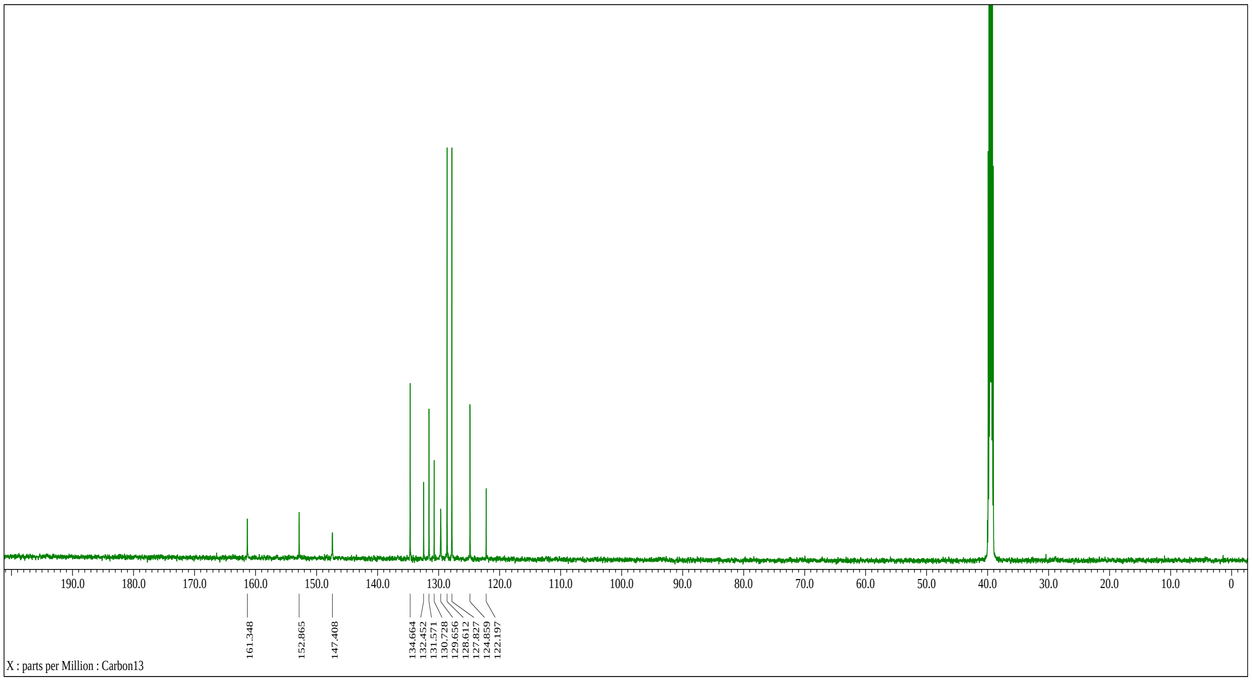
190.0
180.0
170.0
160.0
150.0
140.0
130.0
120.0
110.0
100.0
90.0
80.0
70.0
60.0
50.0
40.0
30.0
20.0
10.0
0
161.348
152.865
147.408
134.664
132.452
131.571
130.728
129.656
128.612
127.827
124.859
122.197
X : parts per Million : Carbon13

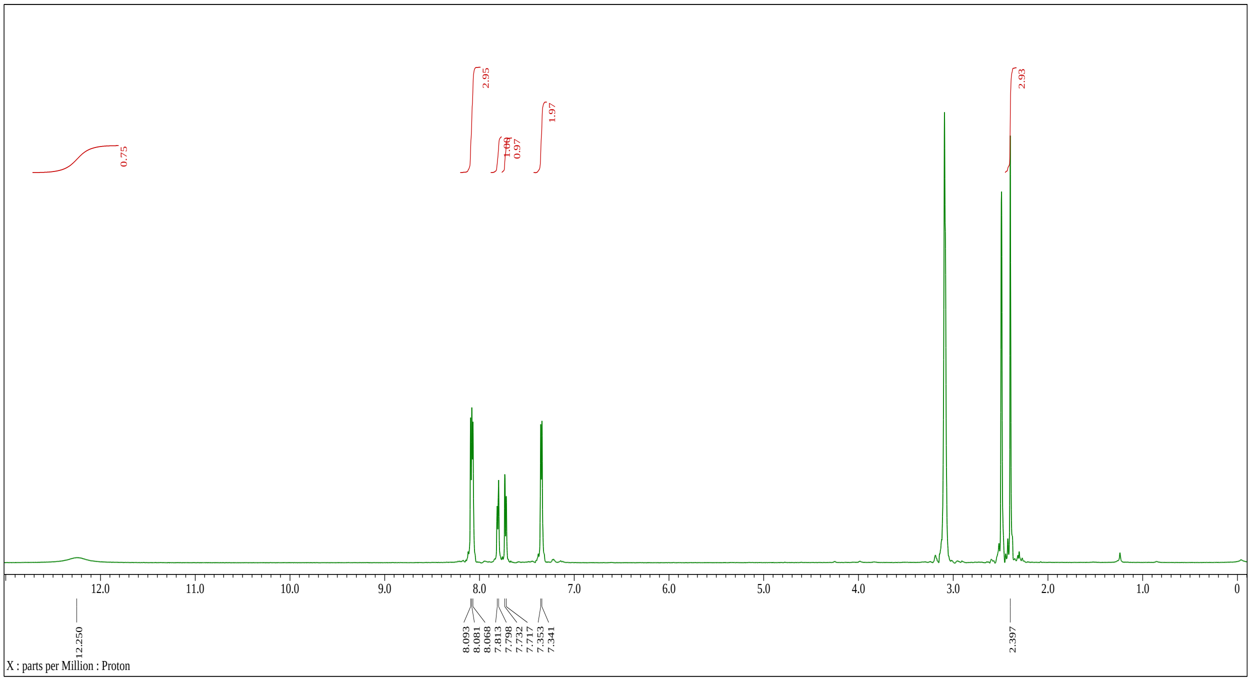
0.75
2.95
1.00
0.97
1.97
2.93
12.0 11.0 10.0 9.0 8.0 7.0 6.0 5.0 4.0 3.0 2.0 1.0 0
12.250
8.093
8.081
8.068
7.813
7.798
7.732
7.717
7.353
7.341
2.397
X : parts per Million : Proton

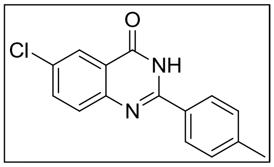
O
NH
Cl
N

4r

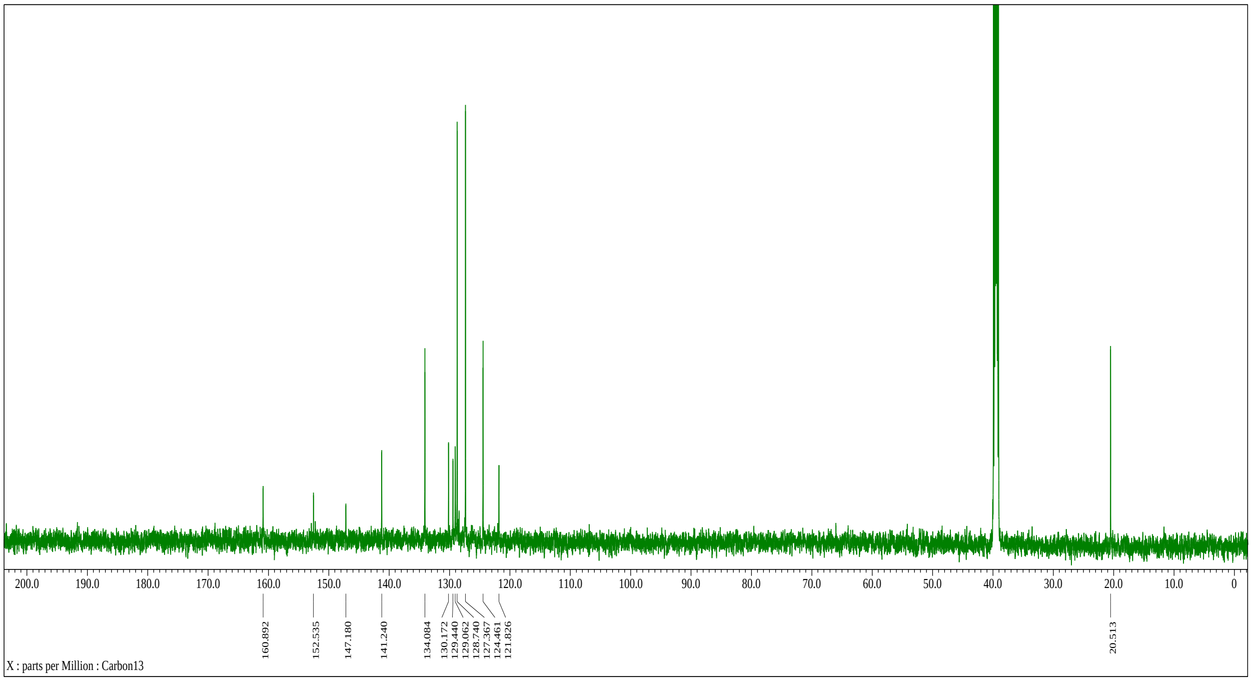
200.0 190.0 180.0 170.0 160.0 150.0 140.0 130.0 120.0 110.0 100.0 90.0 80.0 70.0 60.0 50.0 40.0 30.0 20.0 10.0 0
160.892
152.535
147.180
141.240
134.084
130.172
129.440
129.062
128.740
127.367
124.461
121.826
20.513
X : parts per Million : Carbon13

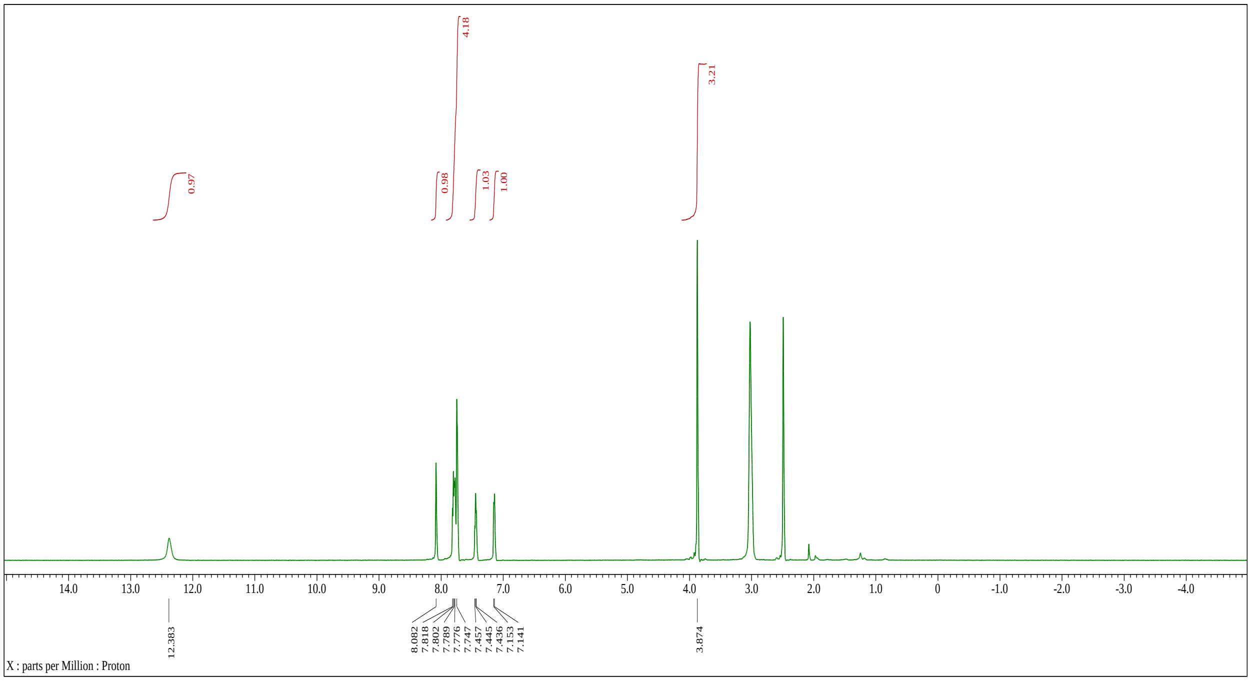
0.97
0.98
4.18
1.03
1.00
3.21
14.0 13.0 12.0 11.0 10.0 9.0 8.0 7.0 6.0 5.0 4.0 3.0 2.0 1.0 0 -1.0 -2.0 -3.0 -4.0
12.383
8.082 7.818 7.802 7.789 7.776 7.747 7.457 7.445 7.436 7.153 7.141
3.874
X : parts per Million : Proton

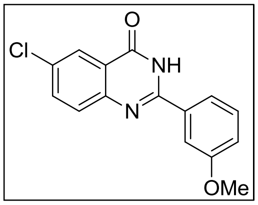
O
Cl
NH
N
OMe

4s

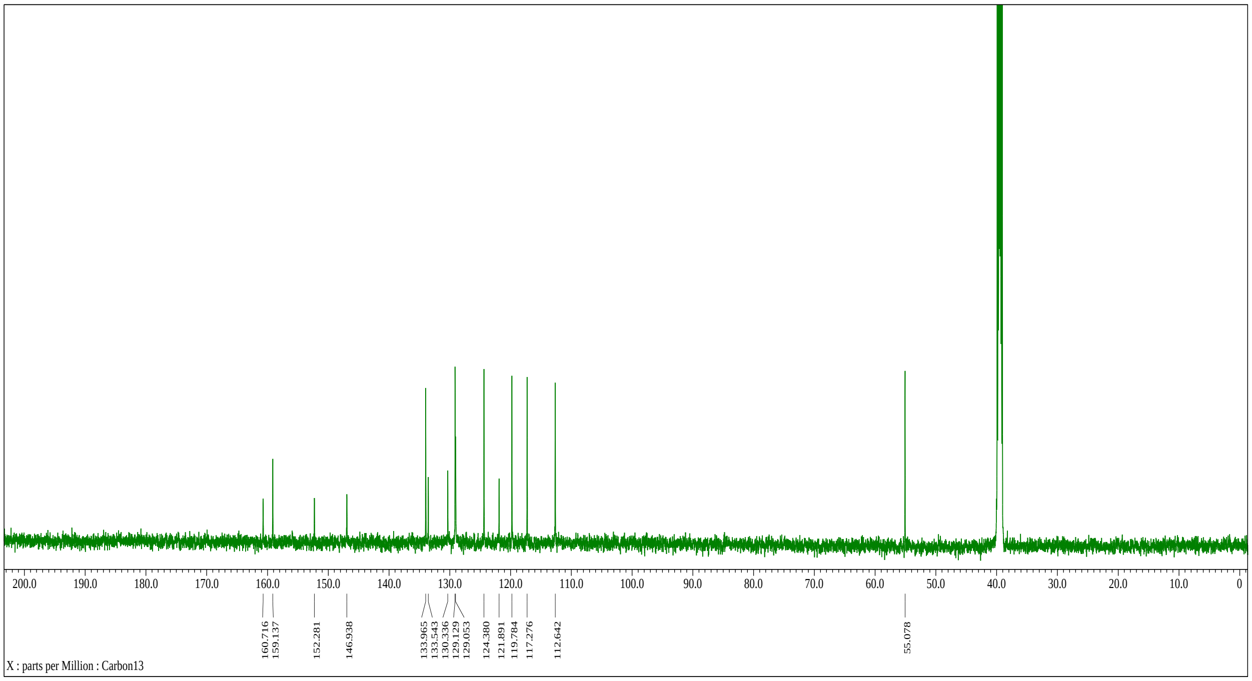
200.0 190.0 180.0 170.0 160.0 150.0 140.0 130.0 120.0 110.0 100.0 90.0 80.0 70.0 60.0 50.0 40.0 30.0 20.0 10.0 0
160.716 159.137
152.281
146.938
133.965 133.543 130.336 129.129 129.053
124.380 121.891 119.784 117.276
112.642
55.078
X : parts per Million : Carbon13

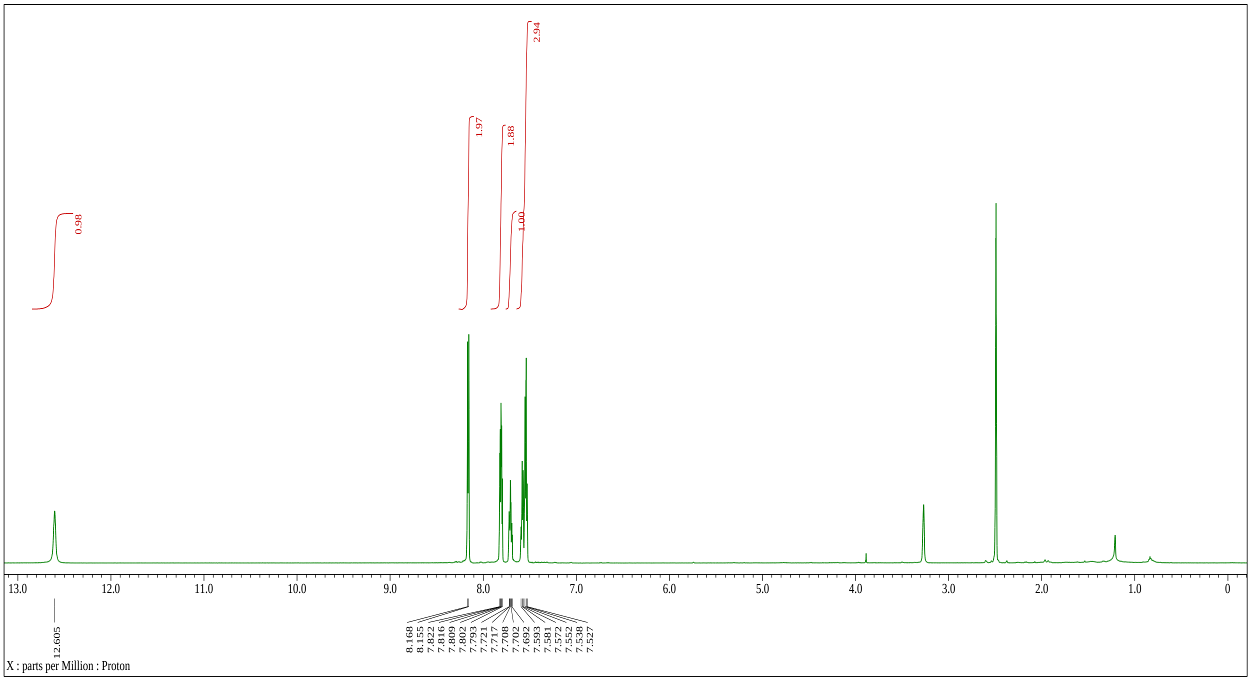

X : parts per Million : Proton

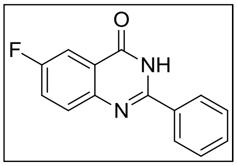

F
O
NH
N

4t

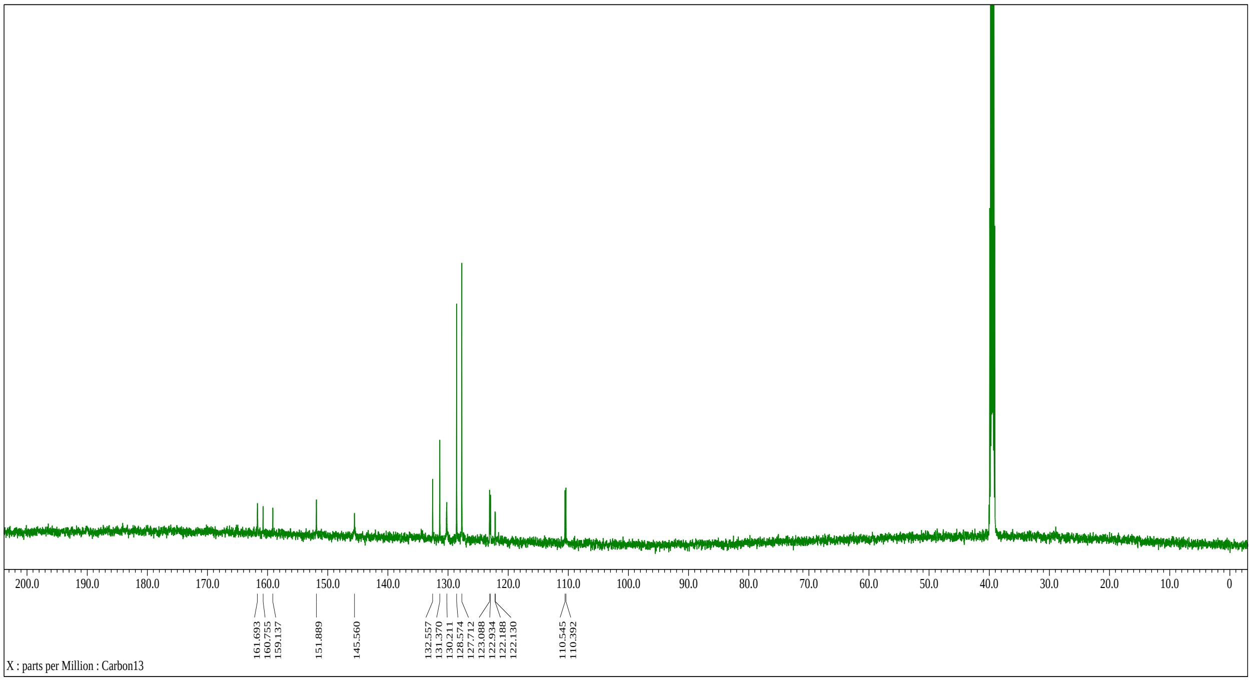

X : parts per Million : Carbon13

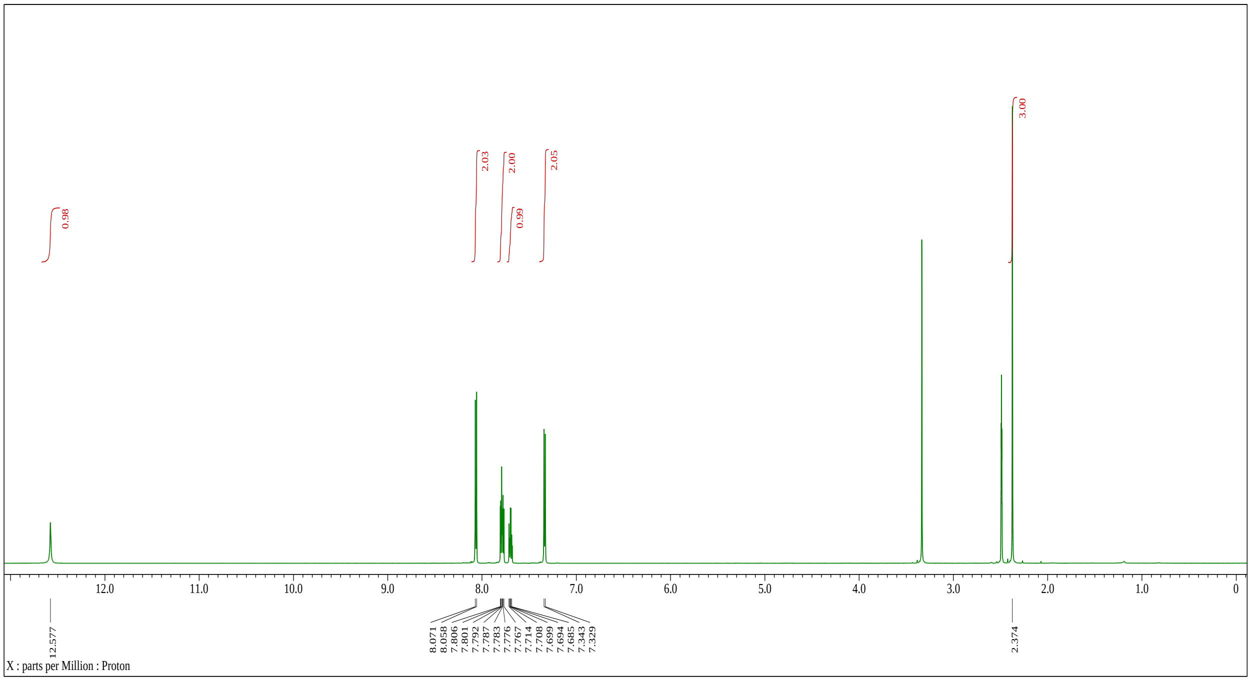
0.98
2.03
2.00
0.99
2.05
3.00
12.0
11.0
10.0
9.0
8.0
7.0
6.0
5.0
4.0
3.0
2.0
1.0
0
12.577
8.071
8.058
7.806
7.801
7.792
7.787
7.783
7.776
7.767
7.714
7.708
7.699
7.694
7.685
7.343
7.329
2.374
X : parts per Million : Proton

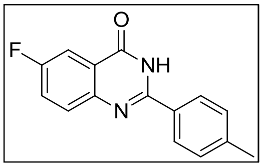
O
F
NH
N

4u

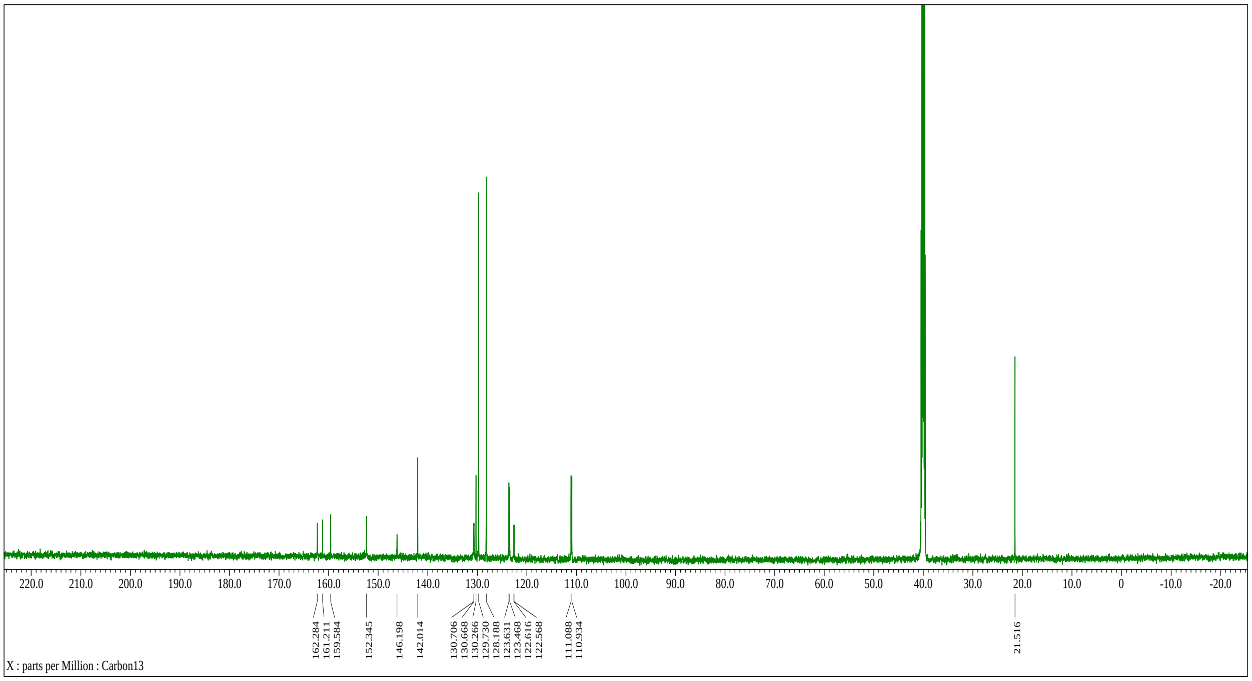
220.0
210.0
200.0
190.0
180.0
170.0
160.0
150.0
140.0
130.0
120.0
110.0
100.0
90.0
80.0
70.0
60.0
50.0
40.0
30.0
20.0
10.0
0
-10.0
-20.0
162.284
161.211
159.584
152.345
146.198
142.014
130.706
130.668
130.266
129.730
128.188
123.631
123.468
122.616
122.568
111.088
110.934
21.516
X : parts per Million : Carbon13

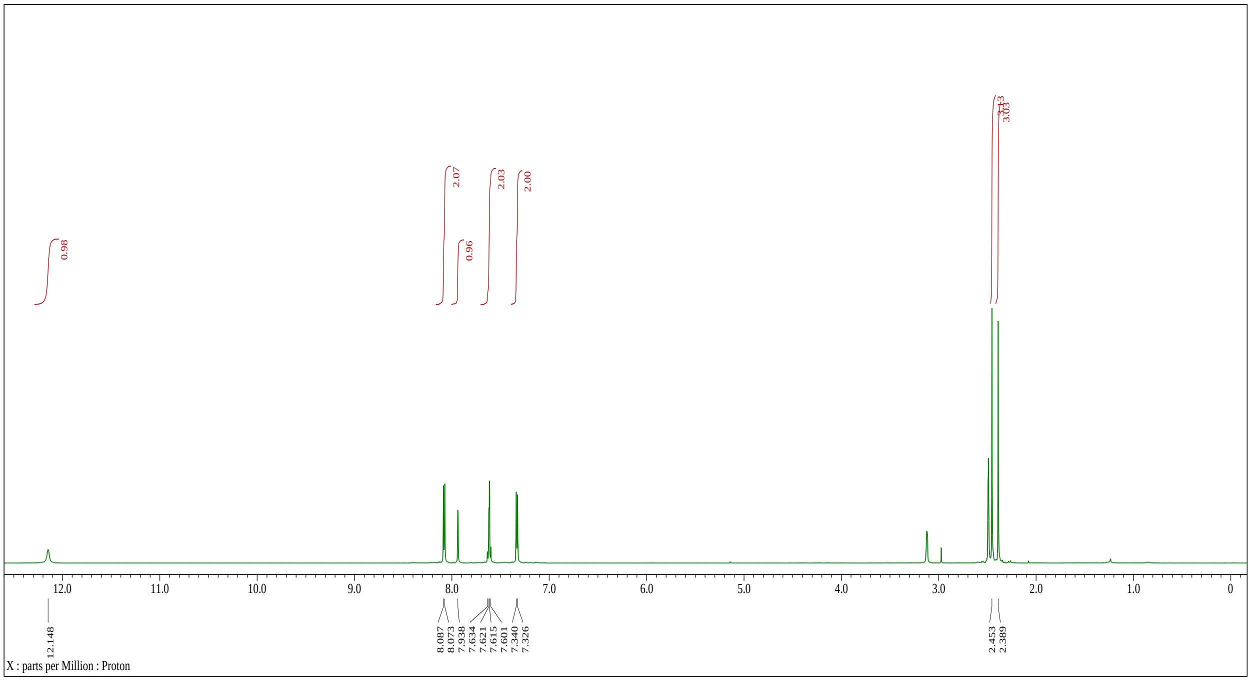
0.98
2.07
0.96
2.03
2.00
3.13
3.03
12.148
8.087
8.073
7.938
7.634
7.621
7.615
7.601
7.340
7.326
2.453
2.389
X : parts per Million : Proton

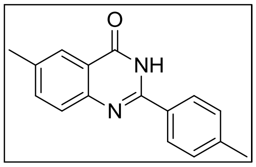
O
NH
N

4v

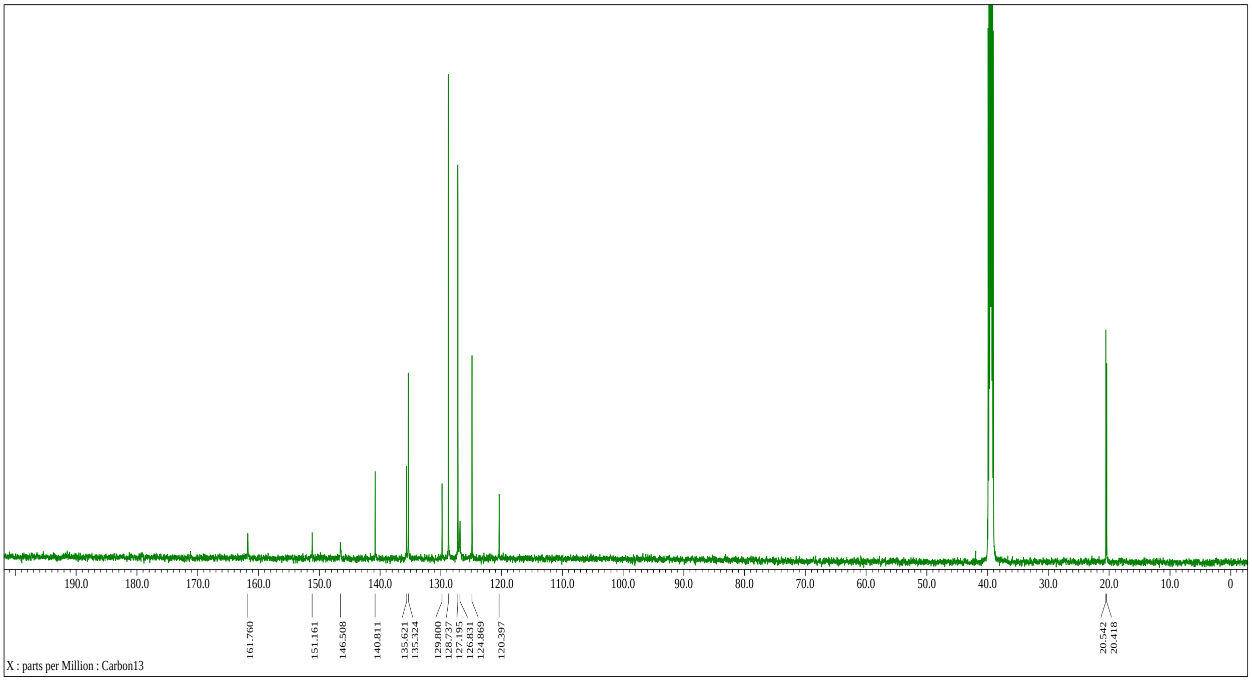
161.760
151.161
146.508
140.811
135.621
135.324
129.800
128.737
127.195
126.831
124.869
120.397
20.542
20.418
X : parts per Million : Carbon13

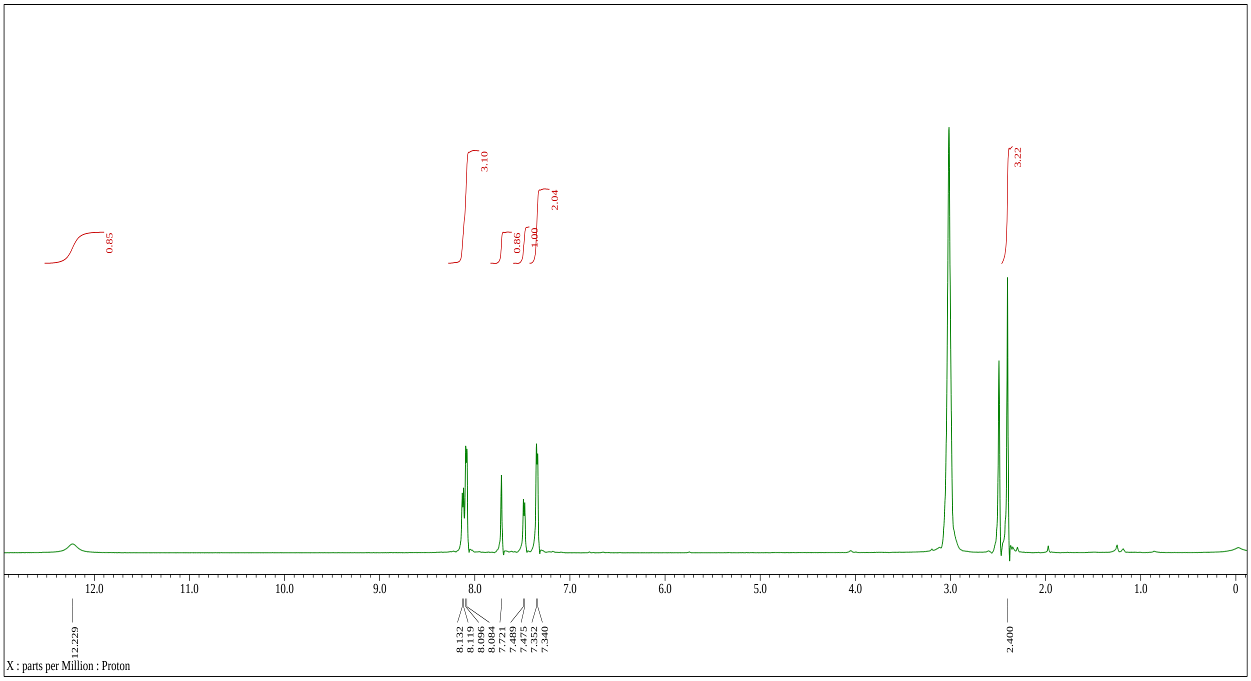
0.85
3.10
0.86
1.00
2.04
3.22
12.0
11.0
10.0
9.0
8.0
7.0
6.0
5.0
4.0
3.0
2.0
1.0
0
12.229
8.132
8.119
8.096
8.084
7.721
7.489
7.475
7.352
7.340
2.400
X : parts per Million : Proton

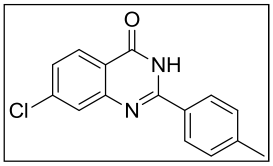
O
NH
Cl
N

4w

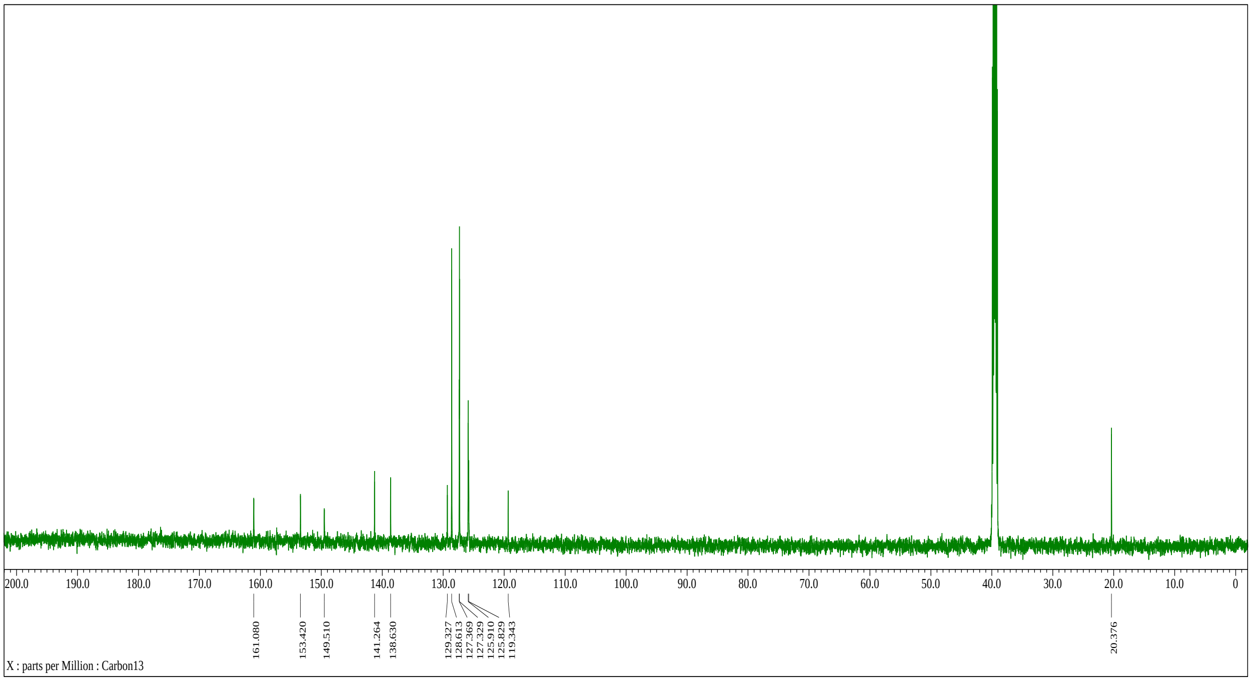
200.0
190.0
180.0
170.0
160.0
150.0
140.0
130.0
120.0
110.0
100.0
90.0
80.0
70.0
60.0
50.0
40.0
30.0
20.0
10.0
0
161.080
153.420
149.510
141.264
138.630
129.327
128.613
127.369
127.329
125.910
125.829
119.343
20.376
X : parts per Million : Carbon13

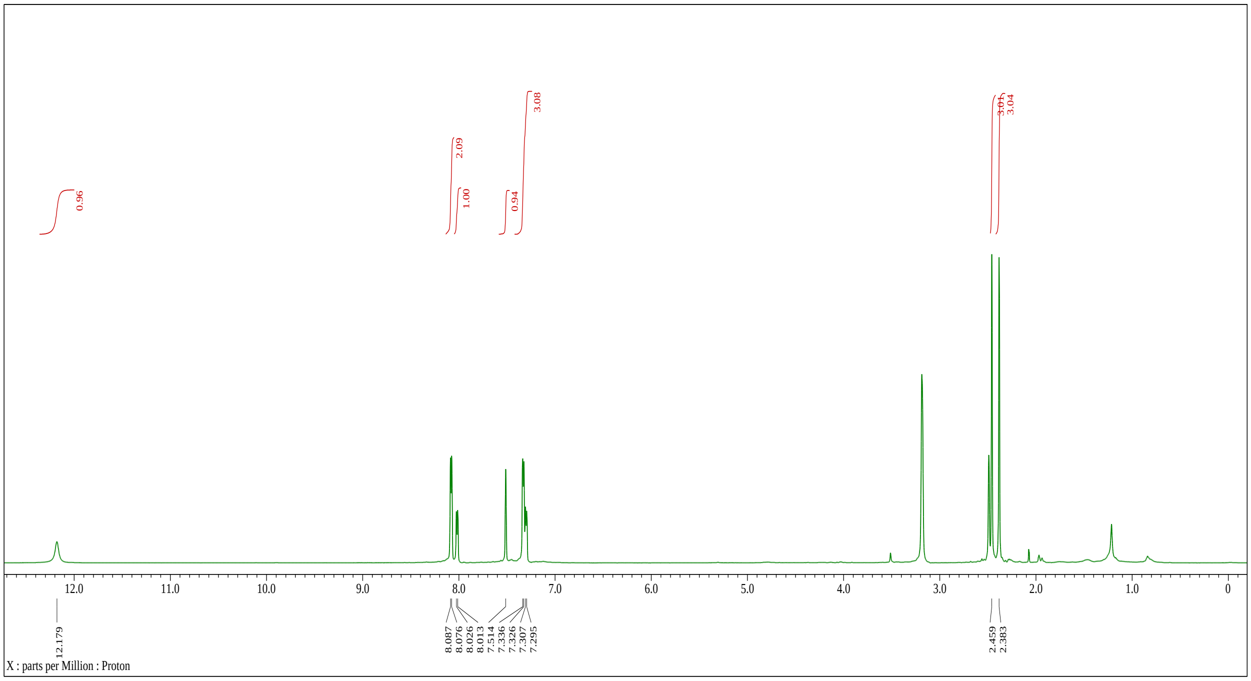
0.96
1.00
2.09
0.94
3.08
3.01
3.04
12.179
8.087
8.076
8.026
8.013
7.514
7.336
7.326
7.307
7.295
2.459
2.383
X : parts per Million : Proton

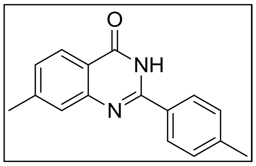

4x

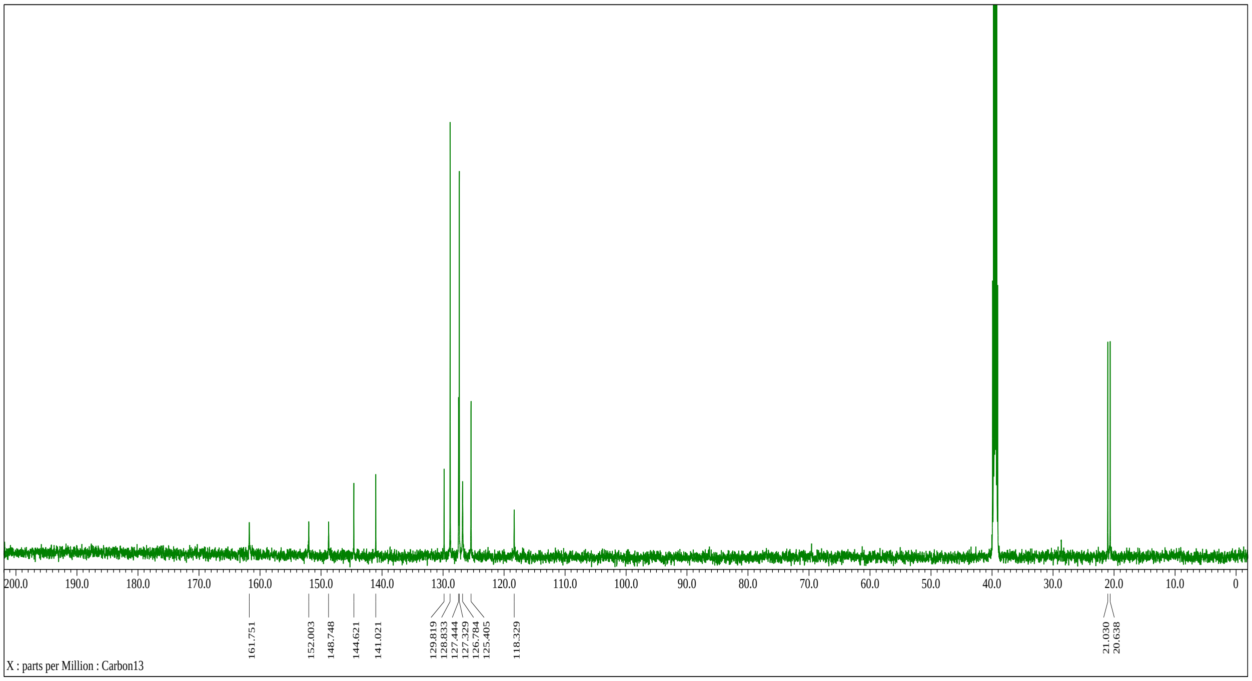
161.751
152.003
148.748
144.621
141.021
129.819
128.833
127.444
127.329
126.784
125.405
118.329
21.030
20.638
X : parts per Million : Carbon13

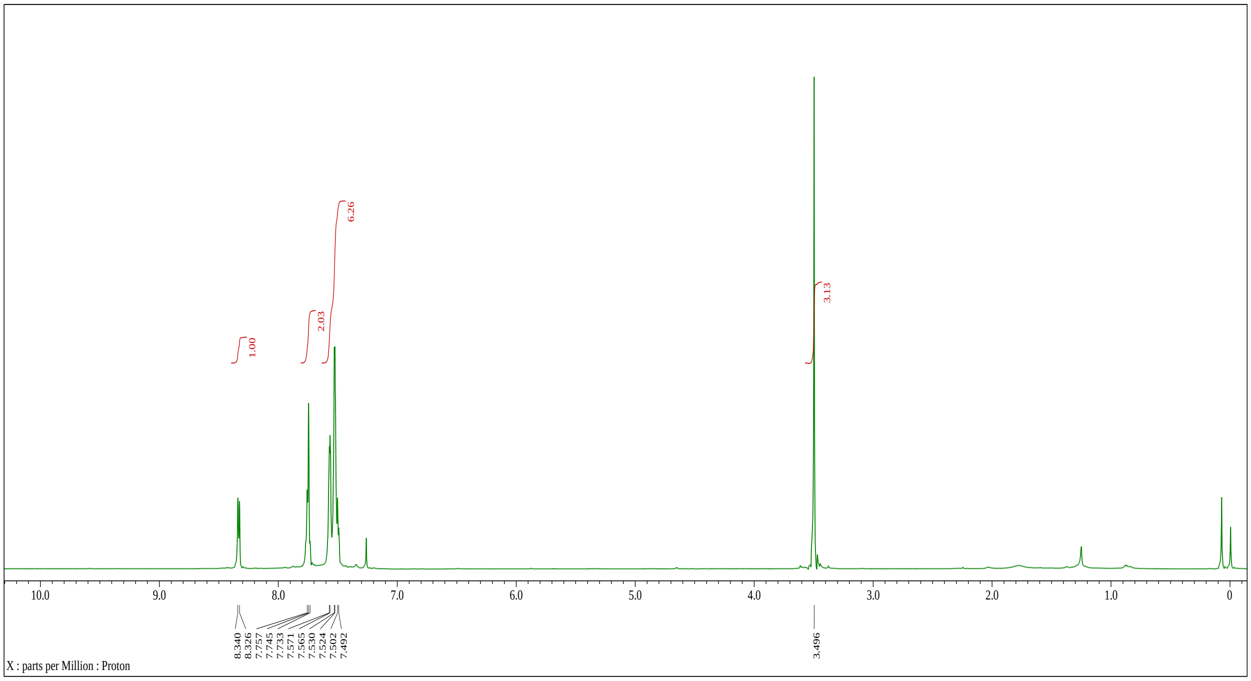
1.00
2.03
6.26
3.13
10.0
9.0
8.0
7.0
6.0
5.0
4.0
3.0
2.0
1.0
0
8.340
8.326
7.757
7.745
7.733
7.571
7.565
7.530
7.524
7.502
7.492
3.496
X : parts per Million : Proton

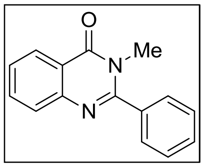
O
N
Me
N

4y

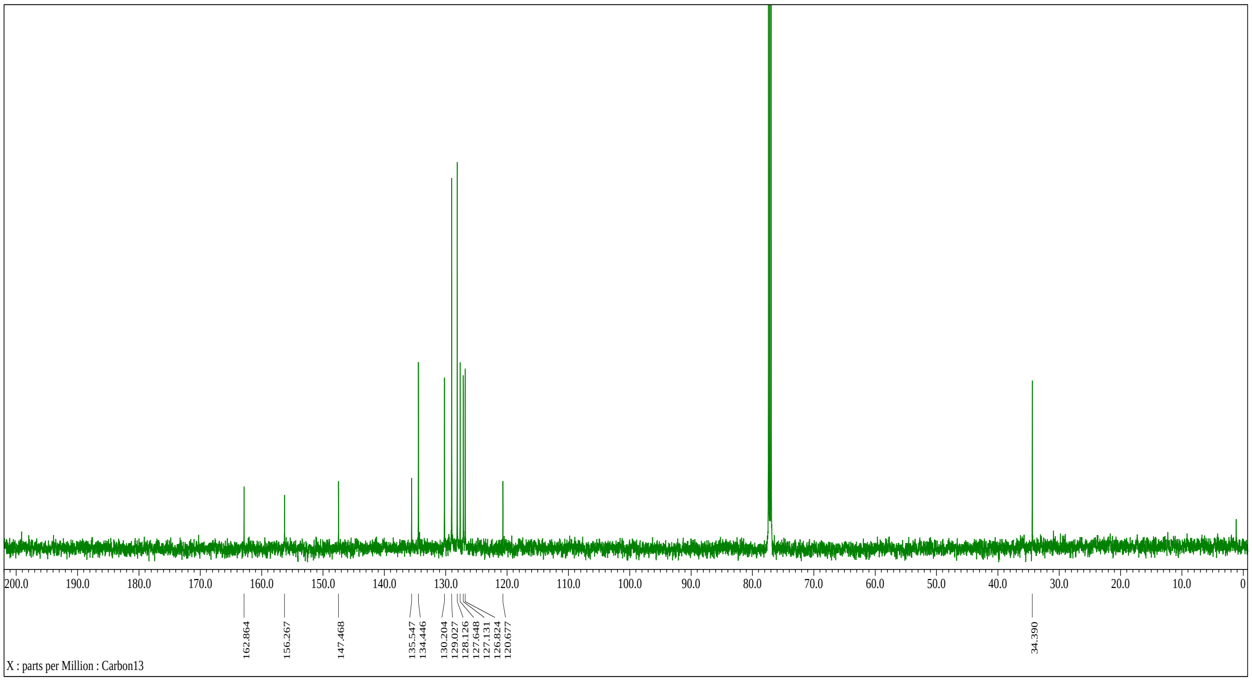
200.0
190.0
180.0
170.0
160.0
150.0
140.0
130.0
120.0
110.0
100.0
90.0
80.0
70.0
60.0
50.0
40.0
30.0
20.0
10.0
0
162.864
156.267
147.468
135.547
134.446
130.204
129.027
128.126
127.648
127.131
126.824
120.677
34.390
X : parts per Million : Carbon13

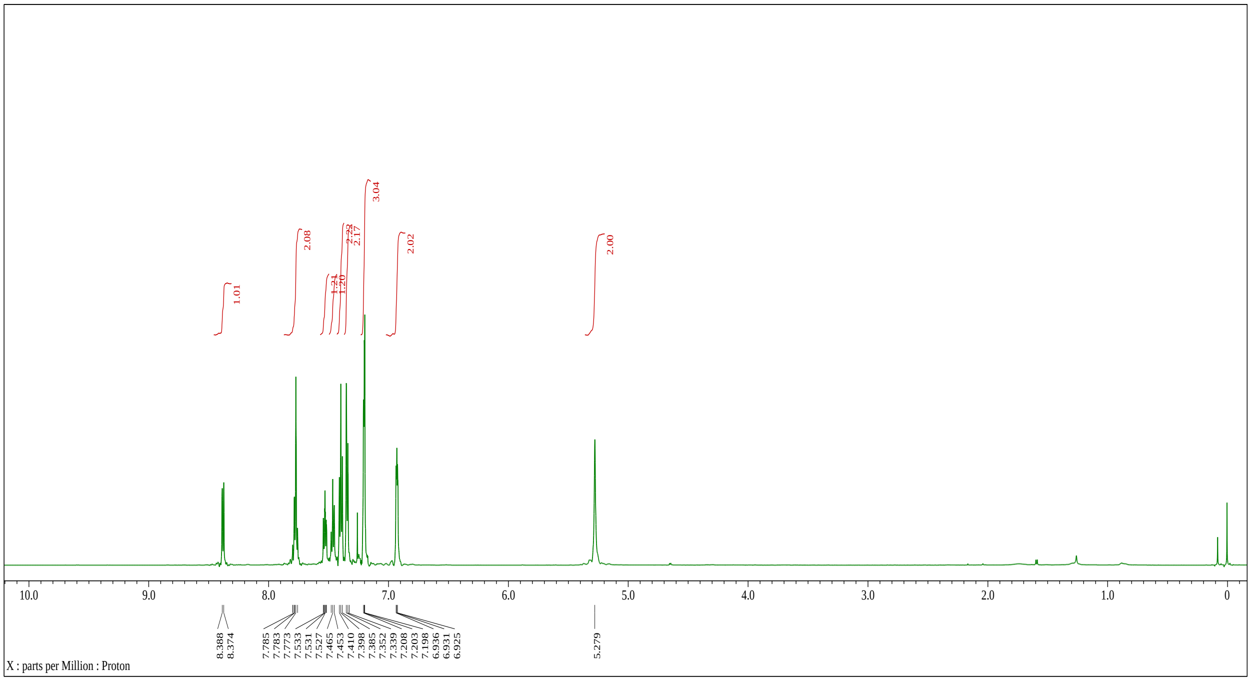
1.01
2.08
1.21
1.20
2.22
2.17
3.04
2.02
2.00
10.0 9.0 8.0 7.0 6.0 5.0 4.0 3.0 2.0 1.0 0
8.388
8.374
7.785
7.783
7.773
7.533
7.531
7.527
7.465
7.453
7.410
7.398
7.385
7.352
7.339
7.208
7.203
7.198
6.936
6.931
6.925
5.279
X : parts per Million : Proton

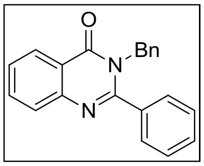
O
N
Bn
N

4z

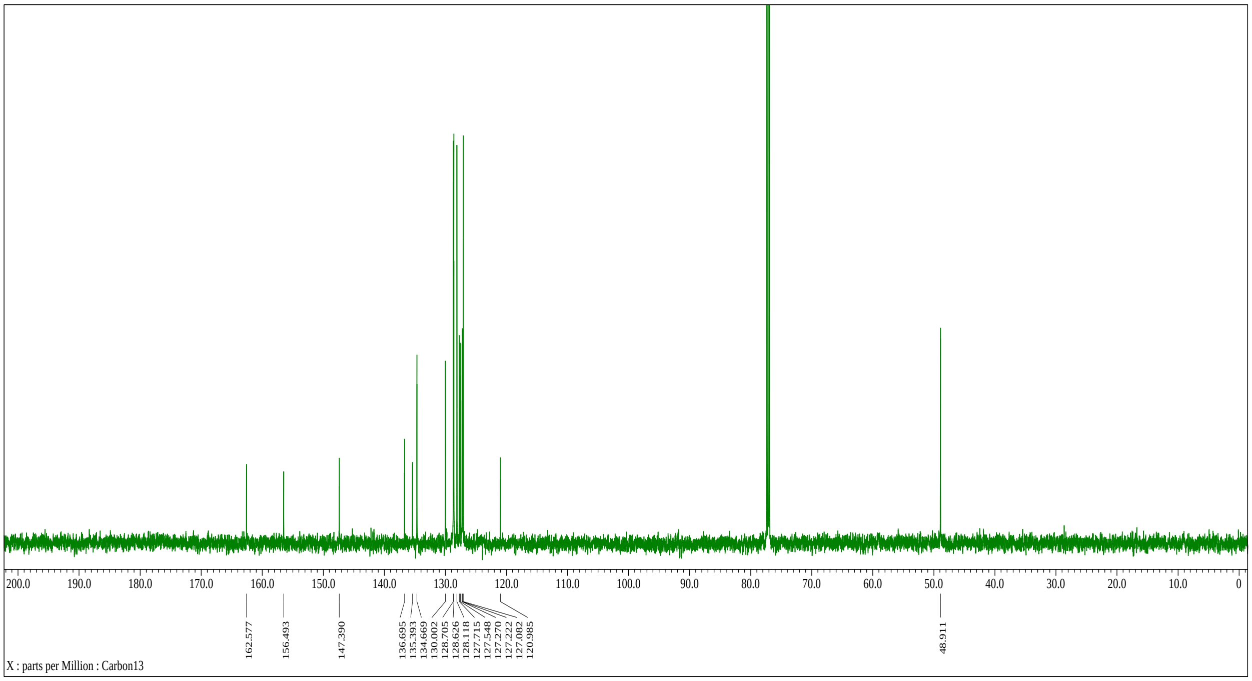
200.0 190.0 180.0 170.0 160.0 150.0 140.0 130.0 120.0 110.0 100.0 90.0 80.0 70.0 60.0 50.0 40.0 30.0 20.0 10.0 0
162.577
156.493
147.390
136.695
135.393
134.669
130.002
128.705
128.626
128.118
127.715
127.548
127.270
127.222
127.082
120.985
48.911
X : parts per Million : Carbon13

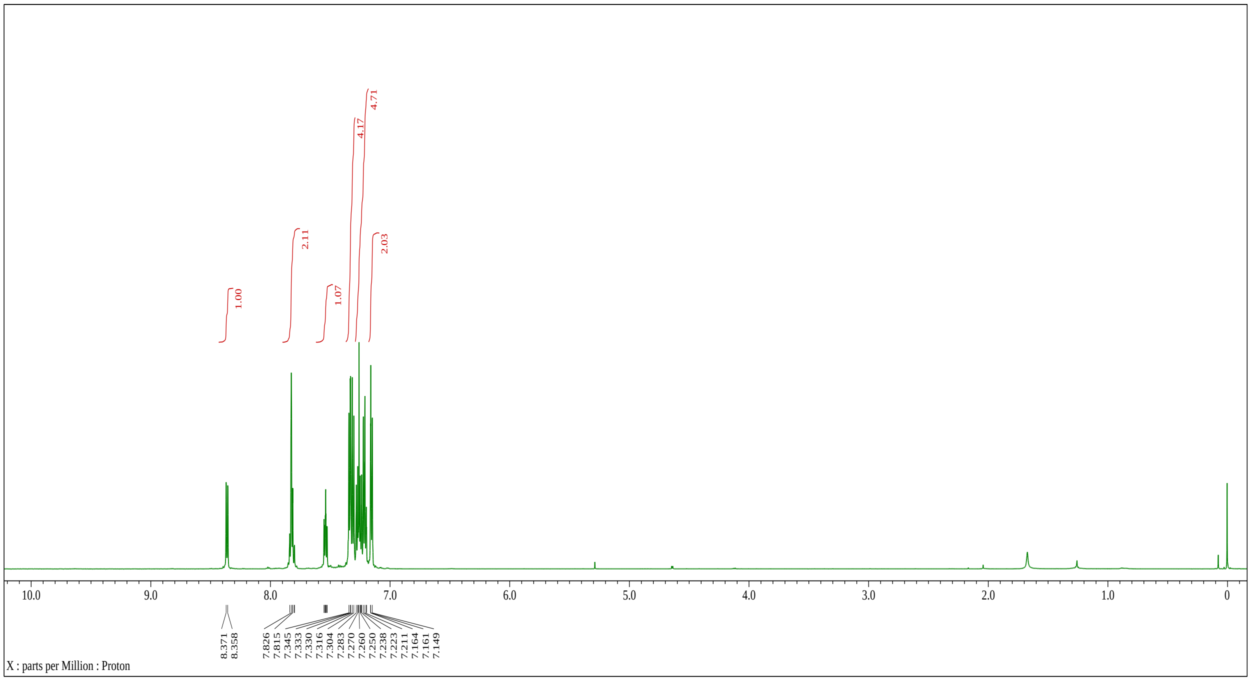

1.00
2.11
1.07
4.17
4.71
2.03
10.0 9.0 8.0 7.0 6.0 5.0 4.0 3.0 2.0 1.0 0
8.371
8.358
7.826
7.815
7.345
7.333
7.330
7.316
7.304
7.283
7.270
7.260
7.250
7.238
7.223
7.211
7.164
7.161
7.149
X : parts per Million : Proton

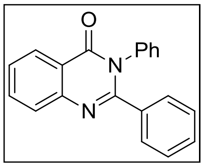

O
N
Ph
N

4za

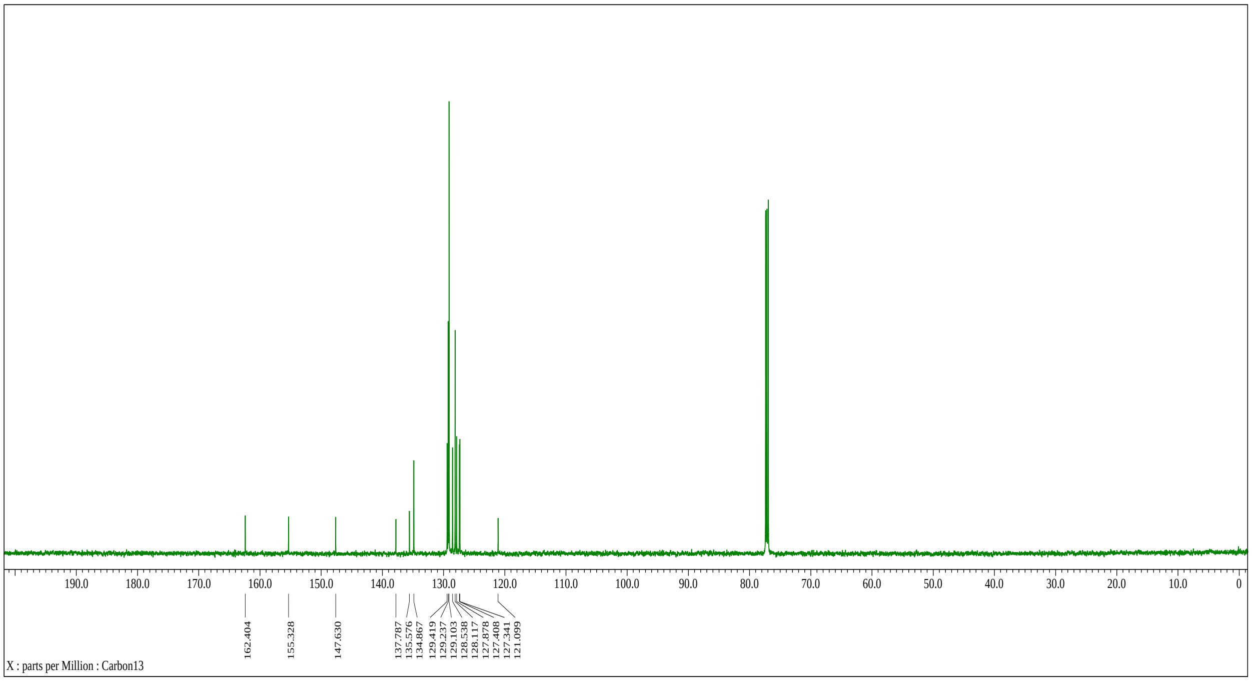

190.0 180.0 170.0 160.0 150.0 140.0 130.0 120.0 110.0 100.0 90.0 80.0 70.0 60.0 50.0 40.0 30.0 20.0 10.0 0
162.404
155.328
147.630
137.787
135.576
134.867
129.419
129.237
129.103
128.538
128.117
127.878
127.408
127.341
121.099
X : parts per Million : Carbon13

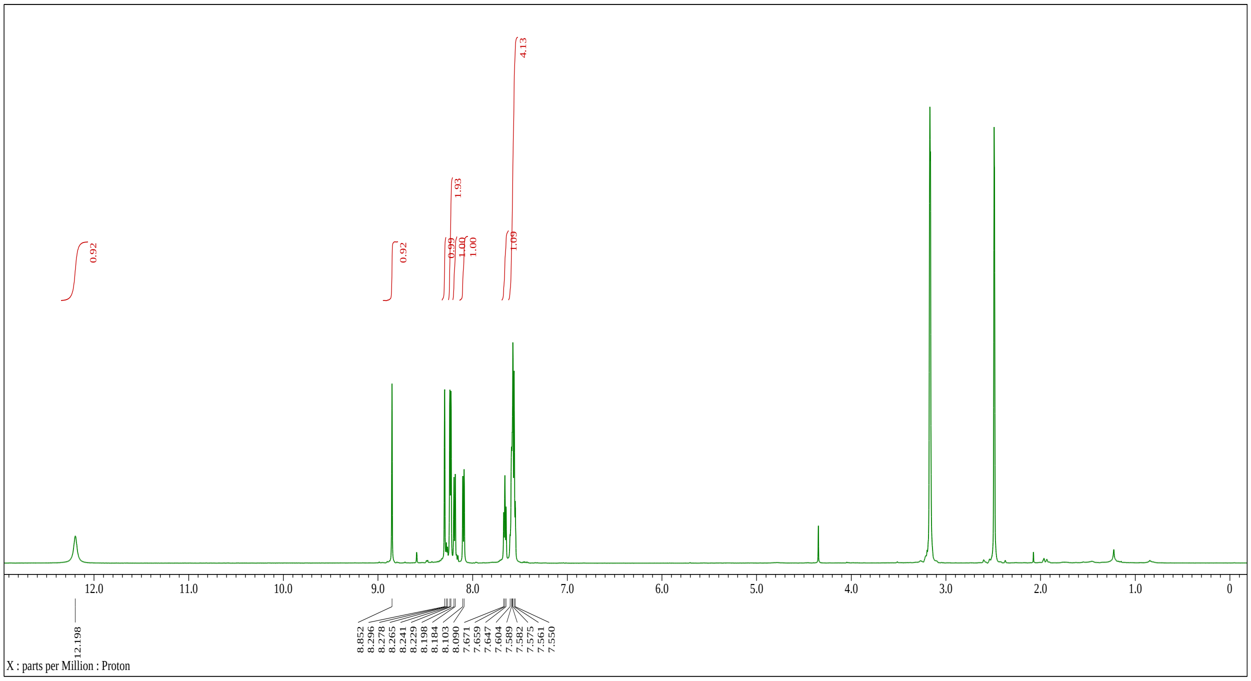
X : parts per Million : Proton

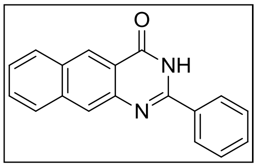
O
NH
N

4zb

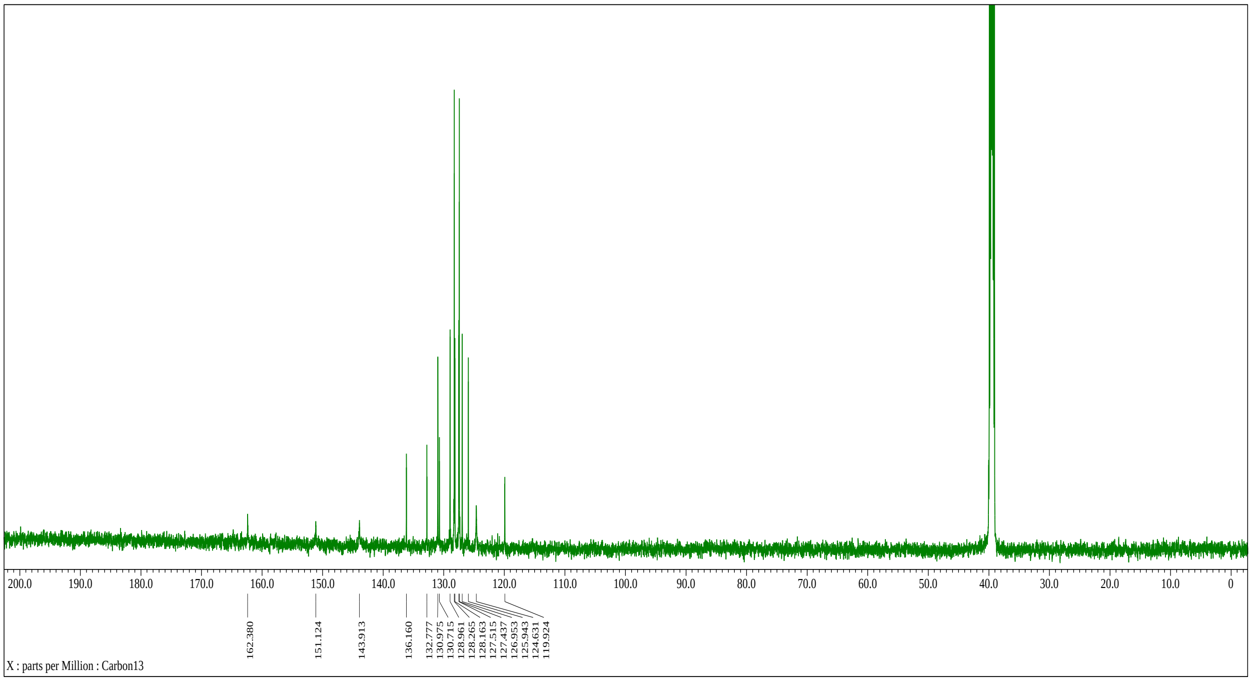
X : parts per Million : Carbon13

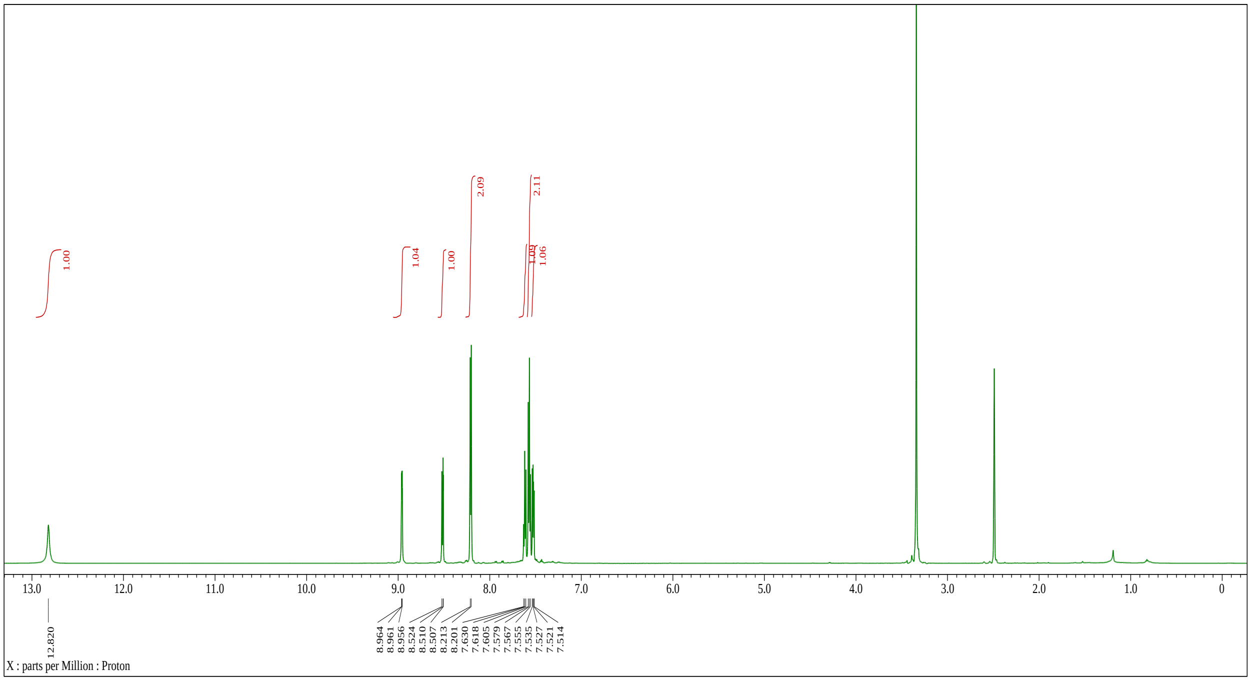
1.00
1.04
1.00
2.09
1.09
2.11
1.06
13.0
12.0
11.0
10.0
9.0
8.0
7.0
6.0
5.0
4.0
3.0
2.0
1.0
0
12.820
8.964
8.961
8.956
8.524
8.510
8.507
8.213
8.201
7.630
7.618
7.605
7.579
7.567
7.555
7.535
7.527
7.521
7.514
X : parts per Million : Proton

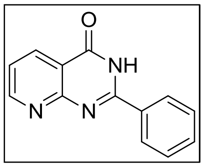
O
NH
N
N

4zc

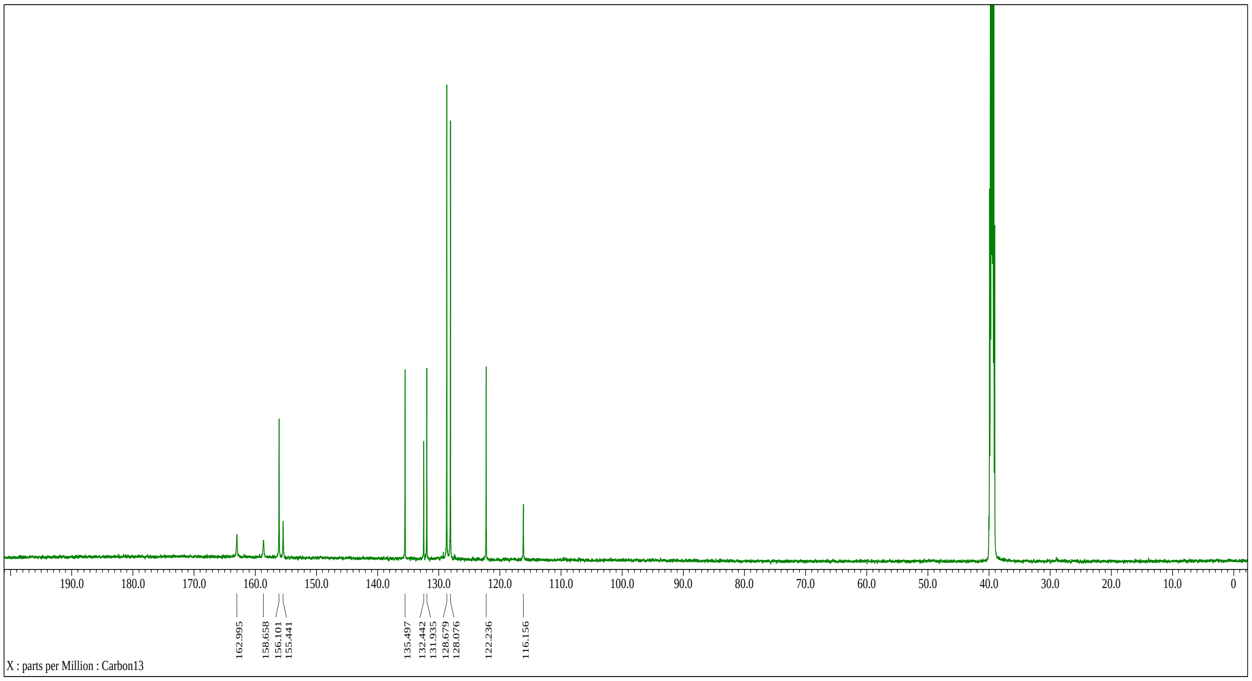
190.0
180.0
170.0
160.0
150.0
140.0
130.0
120.0
110.0
100.0
90.0
80.0
70.0
60.0
50.0
40.0
30.0
20.0
10.0
0
162.995
158.658
156.101
155.441
135.497
132.442
131.935
128.679
128.076
122.236
116.156
X : parts per Million : Carbon13

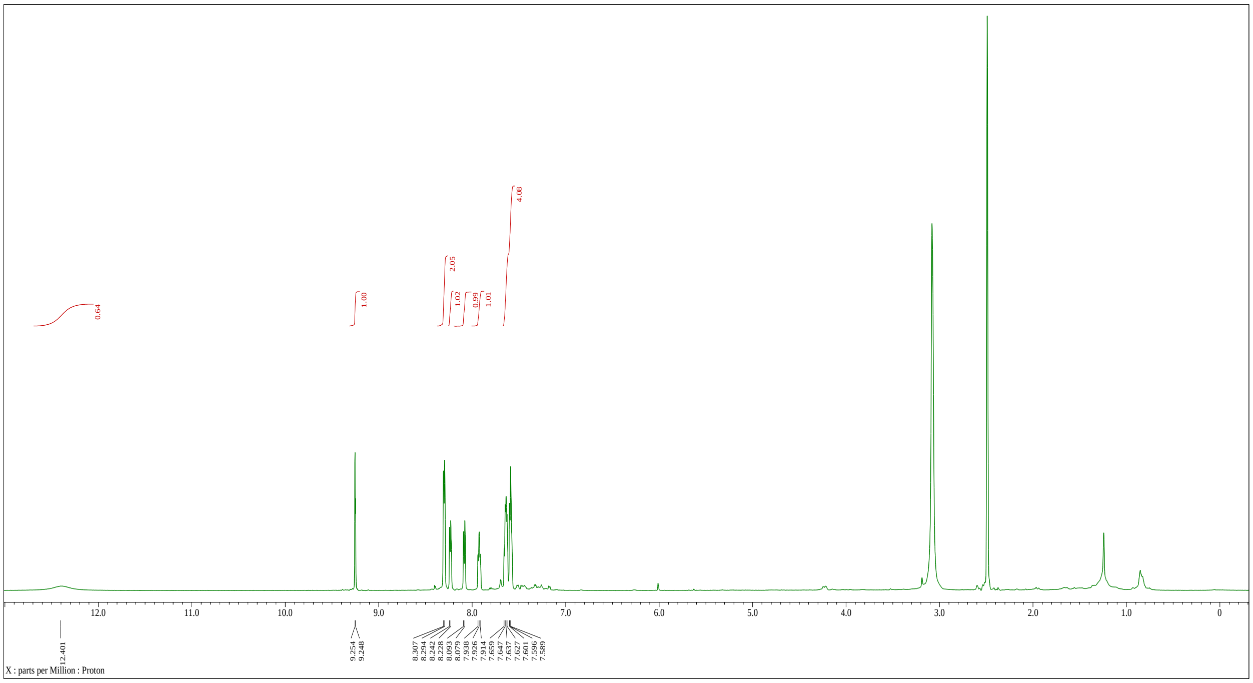
0.64
1.00
2.05
1.02
0.99
1.01
4.08
12.401
9.254
9.248
8.307
8.294
8.242
8.228
8.093
8.079
7.938
7.926
7.914
7.659
7.647
7.637
7.627
7.601
7.596
7.589
X : parts per Million : Proton

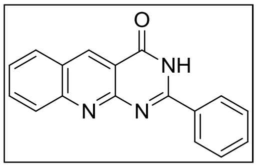
O
NH
N
N

4zd

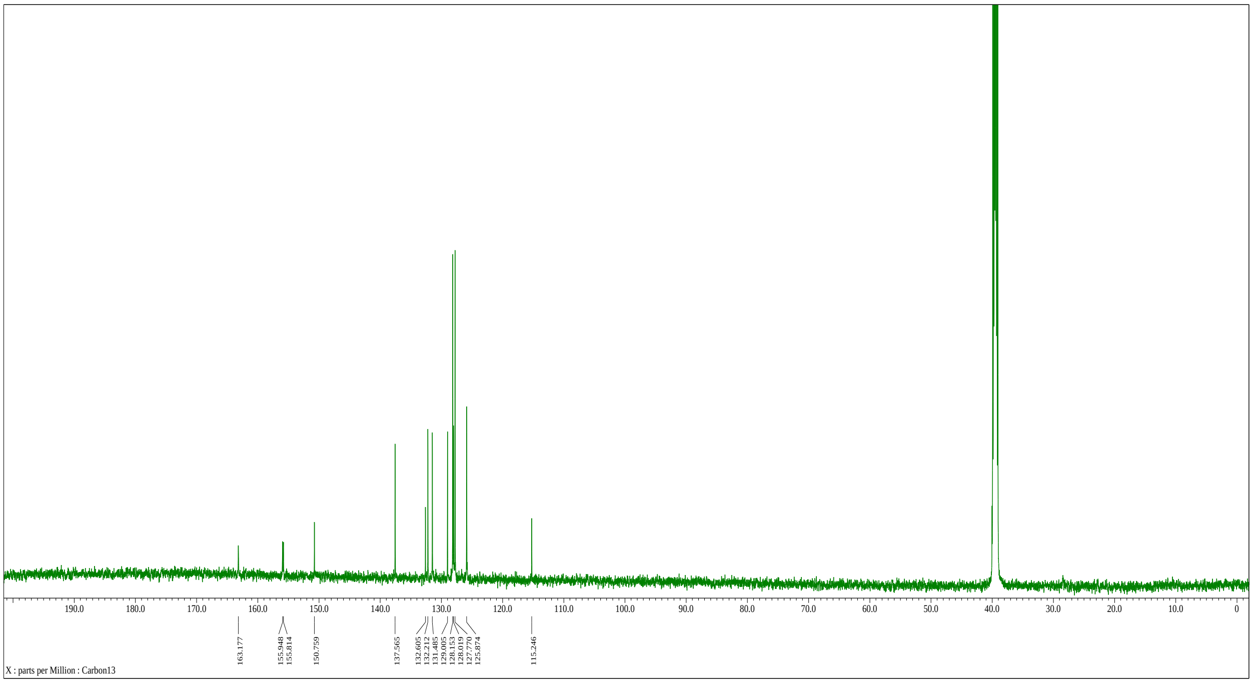
163.177
155.948
155.814
150.759
137.565
132.605
132.212
131.485
129.005
128.153
128.019
127.770
125.874
115.246
X : parts per Million : Carbon13

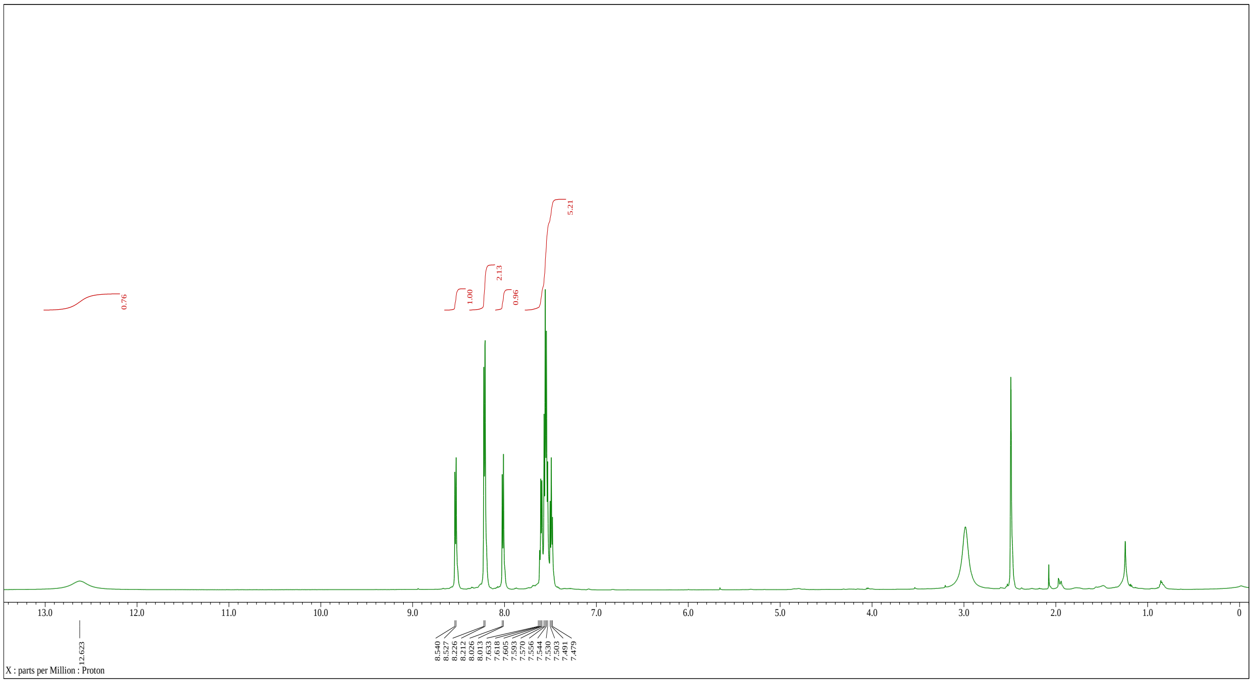
0.76
1.00
2.13
0.96
5.21
12.623
8.540
8.527
8.226
8.212
8.026
8.013
7.631
7.618
7.605
7.593
7.570
7.556
7.544
7.530
7.503
7.491
7.479
X : parts per Million : Proton

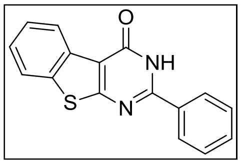
O
NH
S
N

4ze

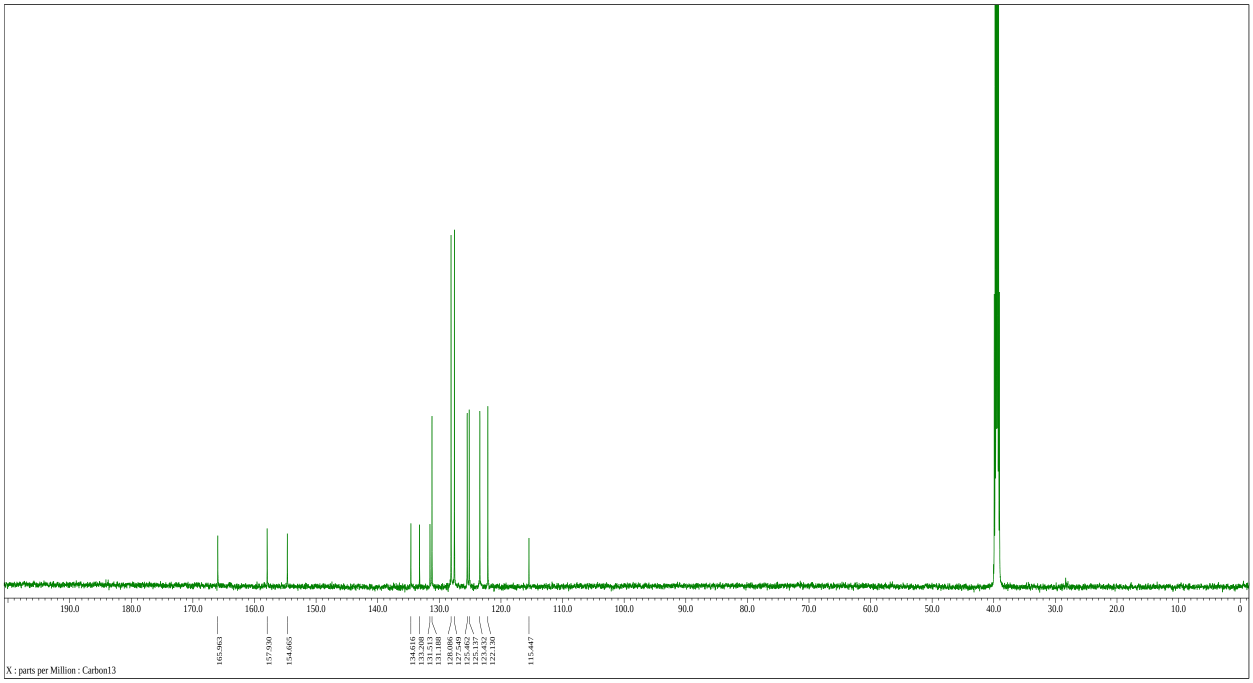
165.963
157.930
154.665
134.616
133.208
131.513
131.188
128.086
127.549
125.462
125.137
123.432
122.130
115.447
X : parts per Million : Carbon13

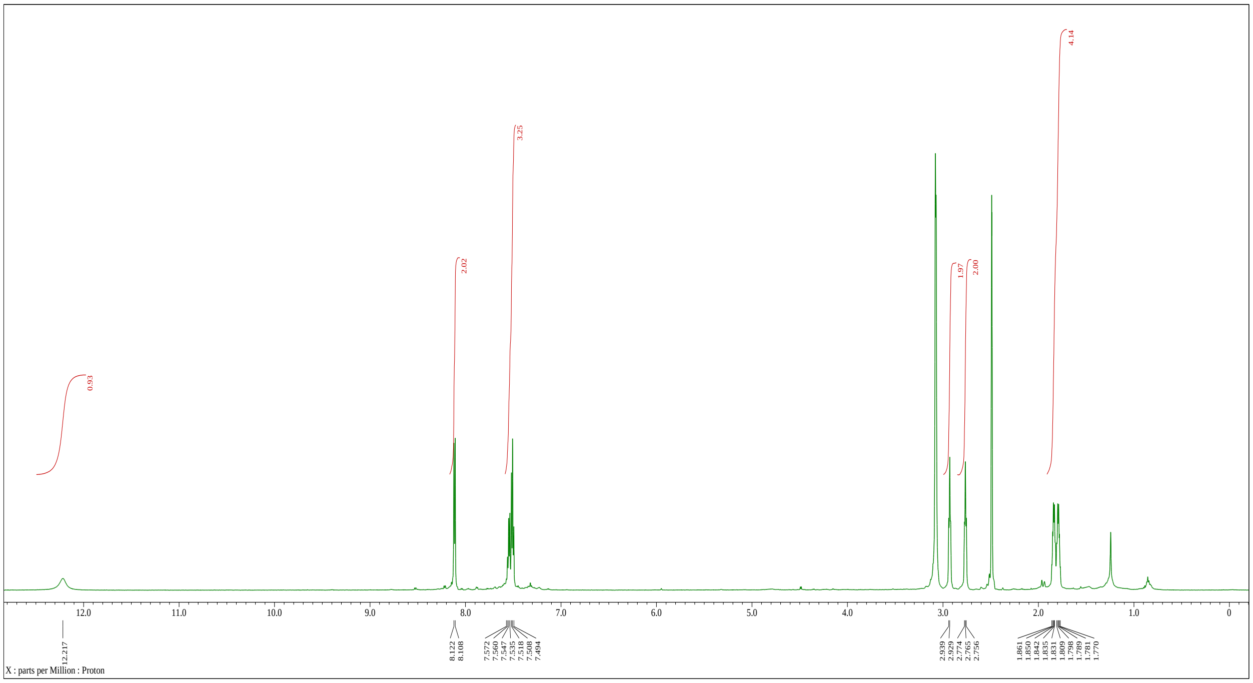
0.93
2.02
3.25
1.97
2.00
4.14
12.0
11.0
10.0
9.0
8.0
7.0
6.0
5.0
4.0
3.0
2.0
1.0
0
12.217
8.122
8.108
7.572
7.560
7.547
7.535
7.518
7.508
7.494
2.939
2.929
2.774
2.765
2.756
1.861
1.850
1.842
1.835
1.831
1.809
1.798
1.789
1.781
1.770
X : parts per Million : Proton

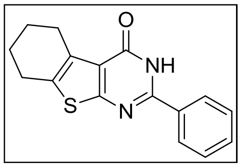
O
NH
S
N

4zf

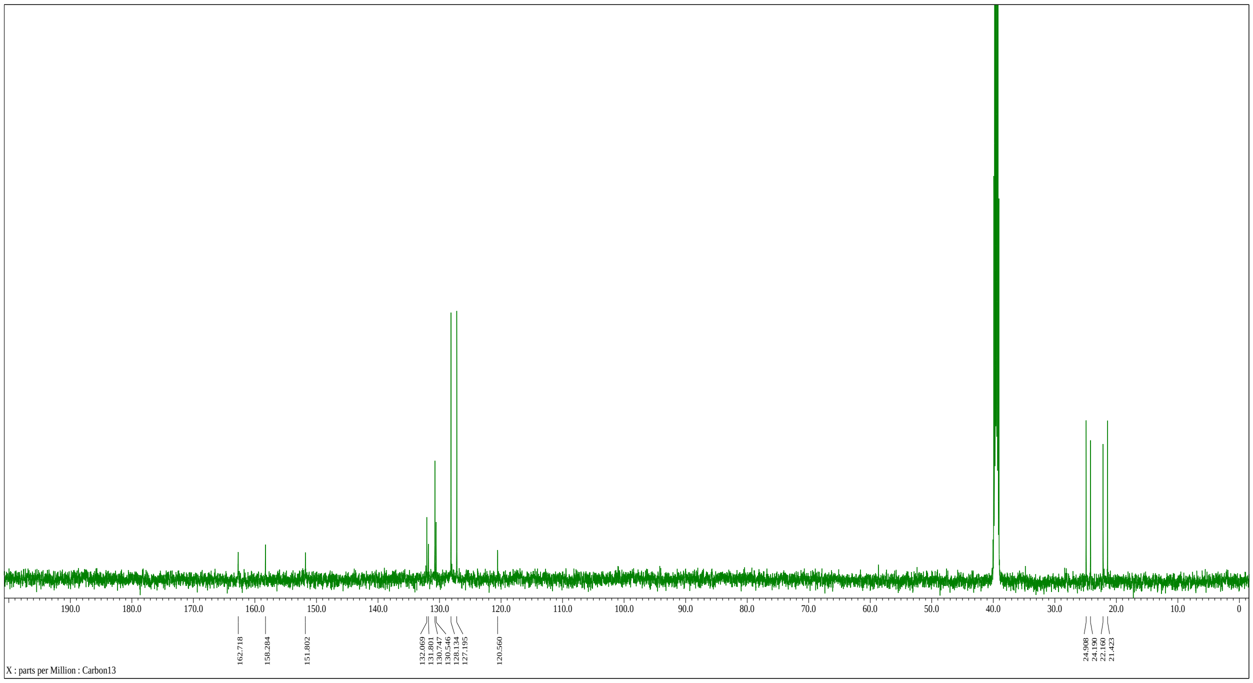
190.0
180.0
170.0
160.0
150.0
140.0
130.0
120.0
110.0
100.0
90.0
80.0
70.0
60.0
50.0
40.0
30.0
20.0
10.0
0
162.718
158.284
151.802
132.069
131.801
130.747
130.546
128.134
127.195
120.560
24.908
24.190
22.160
21.423
X : parts per Million : Carbon13

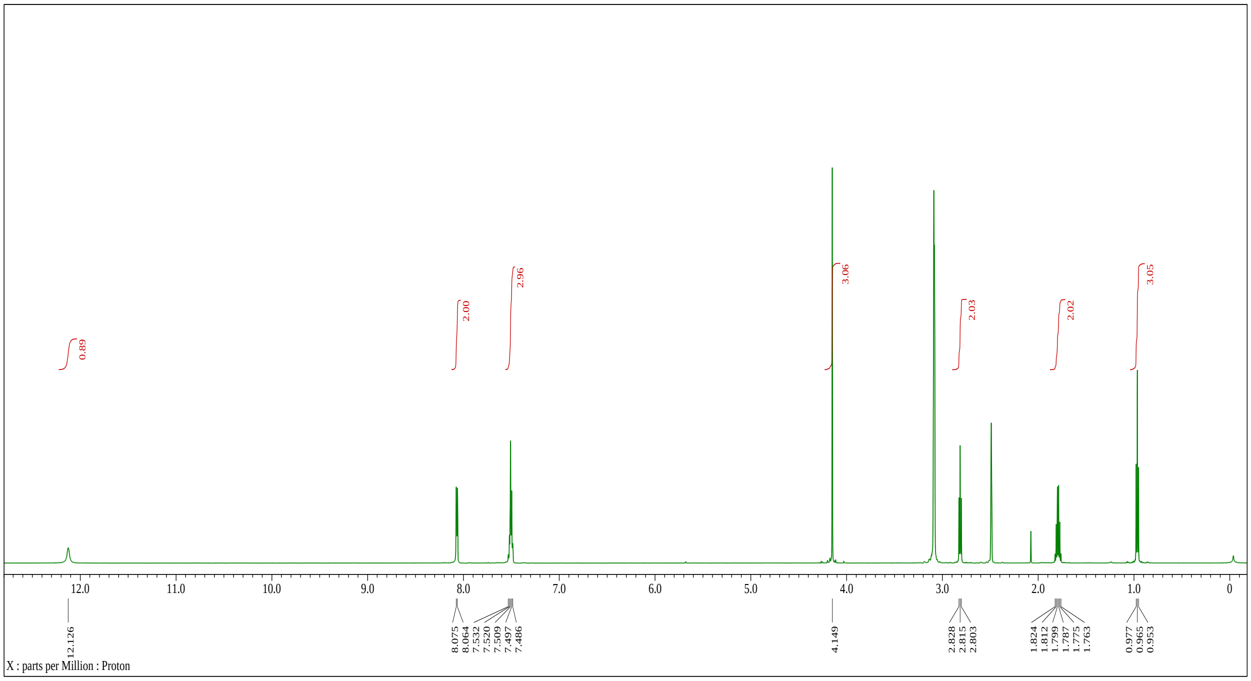
0.89
2.00
2.96
3.06
2.03
2.02
3.05
12.0
11.0
10.0
9.0
8.0
7.0
6.0
5.0
4.0
3.0
2.0
1.0
0
12.126
8.075
8.064
7.532
7.520
7.509
7.497
7.486
4.149
2.828
2.815
2.803
1.824
1.812
1.799
1.787
1.775
1.763
0.977
0.965
0.953
X : parts per Million : Proton

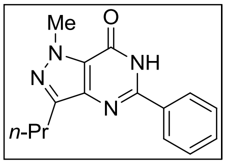
Me
O
N
NH
N
N
n-Pr

4zg

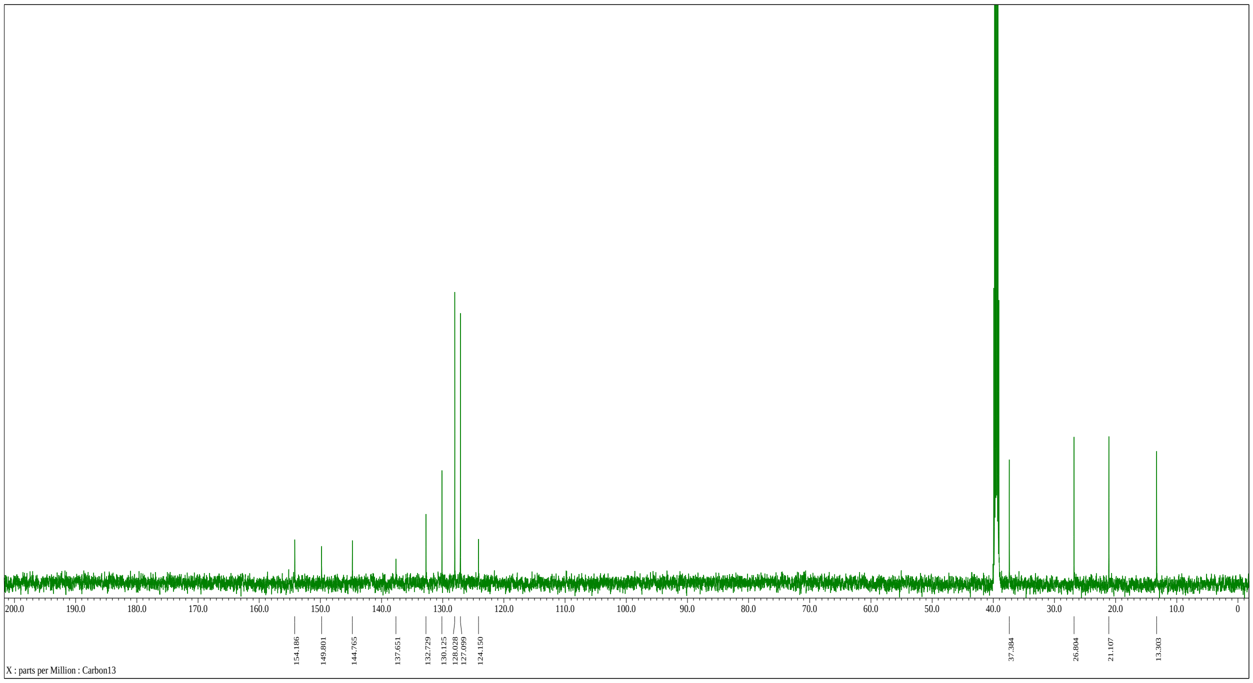
200.0
190.0
180.0
170.0
160.0
150.0
140.0
130.0
120.0
110.0
100.0
90.0
80.0
70.0
60.0
50.0
40.0
30.0
20.0
10.0
0
154.186
149.801
144.765
137.651
132.729
130.125
128.028
127.099
124.150
37.384
26.804
21.107
13.303
X : parts per Million : Carbon13

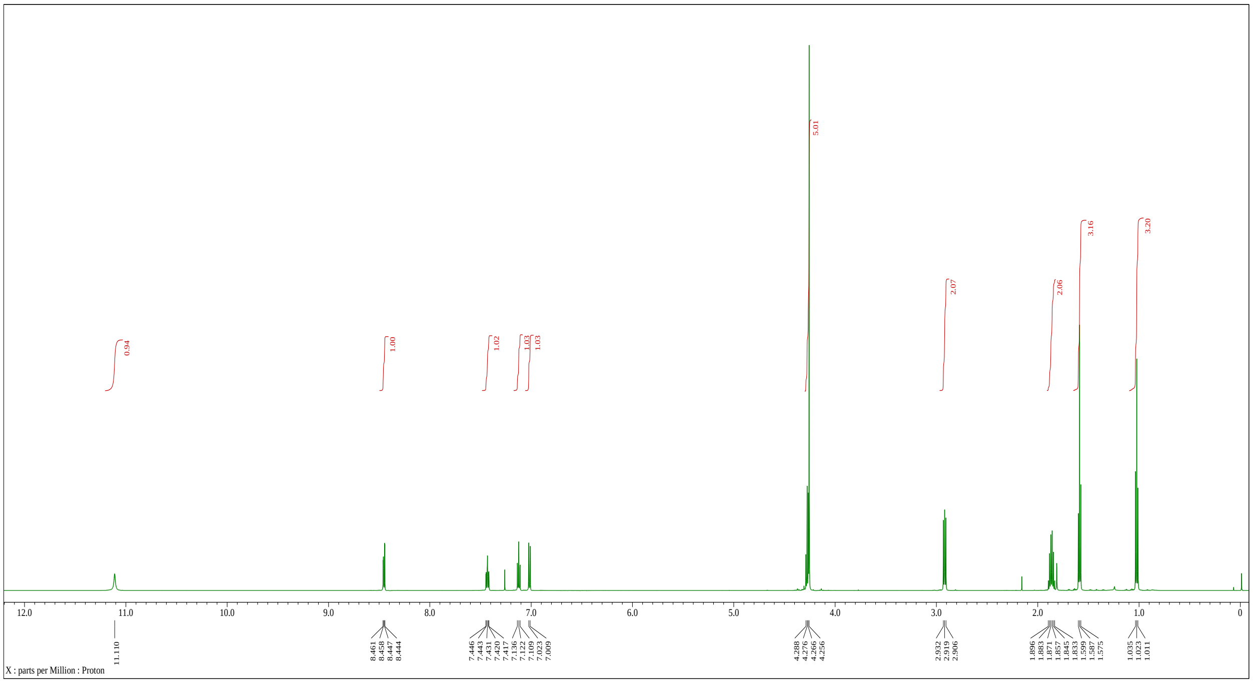

0.94
1.00
1.02
1.03
1.03
5.01
2.07
2.06
3.16
3.20
11.110
8.461
8.458
8.447
8.444
7.446
7.443
7.431
7.420
7.417
7.136
7.122
7.109
7.023
7.009
4.288
4.276
4.266
4.256
2.932
2.919
2.906
1.896
1.883
1.871
1.857
1.845
1.833
1.599
1.587
1.575
1.035
1.023
1.011
X : parts per Million : Proton

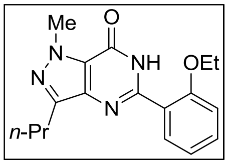

Me
O
N
NH
OEt
N
N
n-Pr

4zh

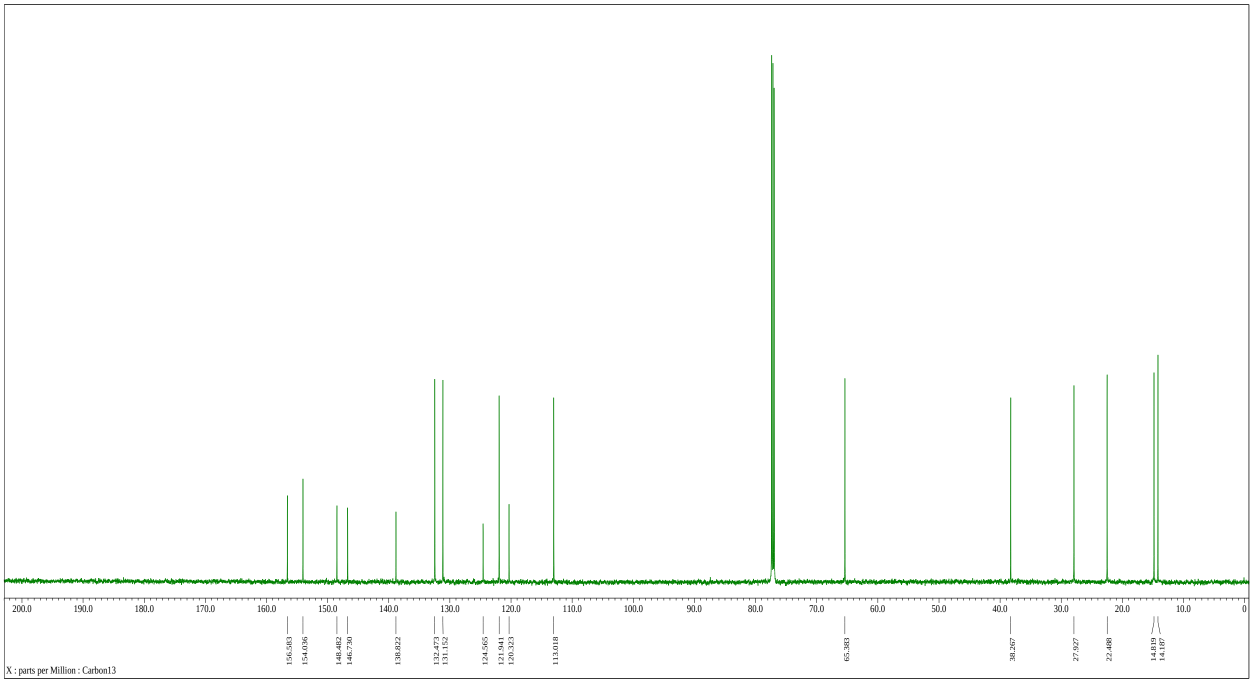

156.583
154.036
148.482
146.730
138.822
132.473
131.152
124.565
121.941
120.323
113.018
65.383
38.267
27.927
22.488
14.819
14.187
X : parts per Million : Carbon13

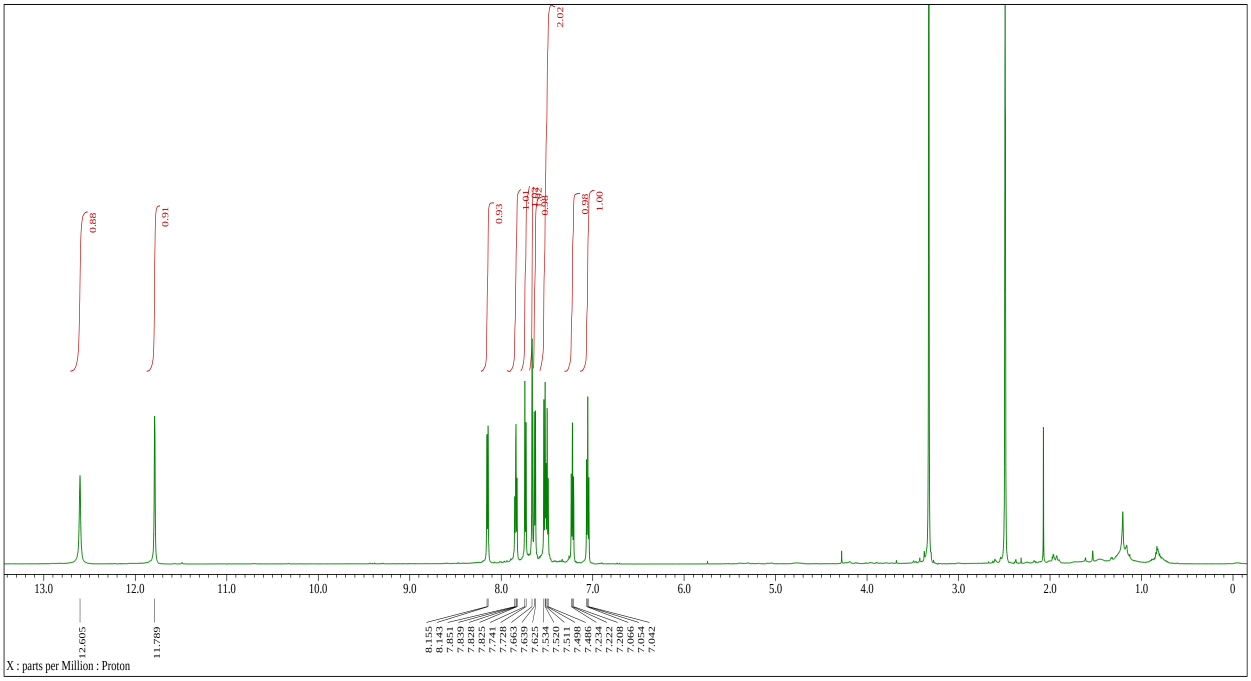
X : parts per Million : Proton

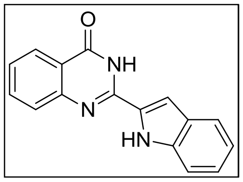
O
NH
N
HN

5

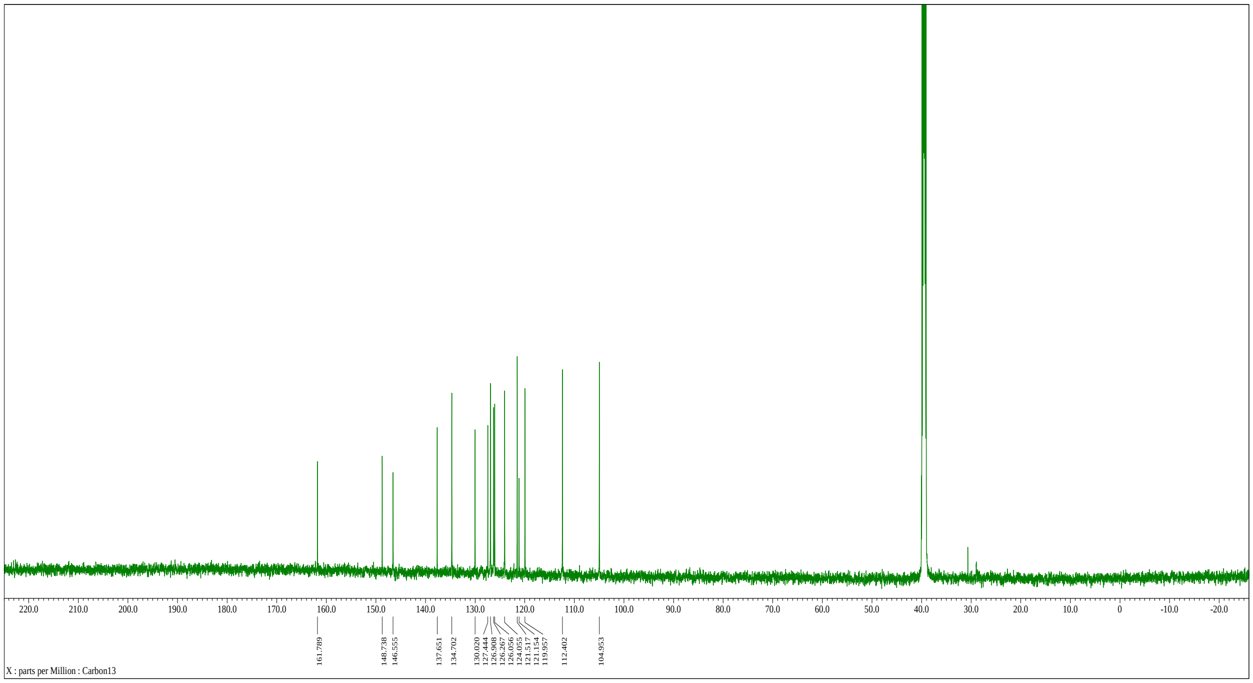
X : parts per Million : Carbon13

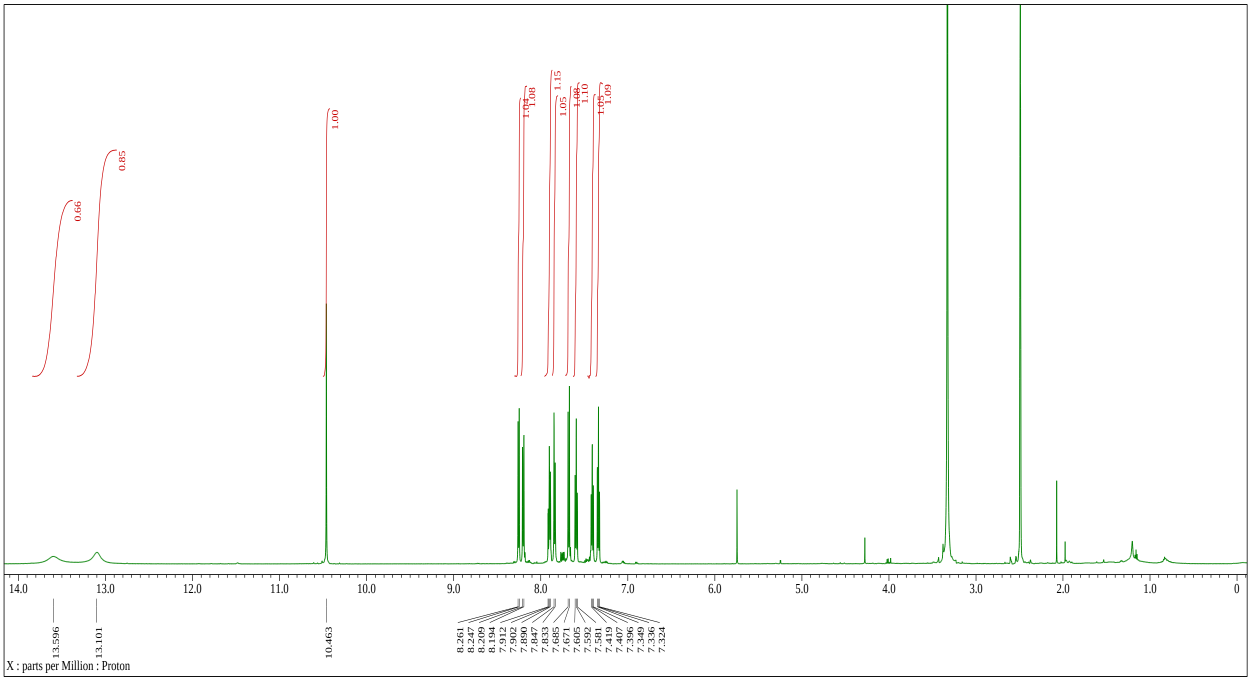

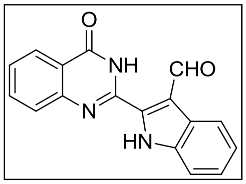

Bouchardatine

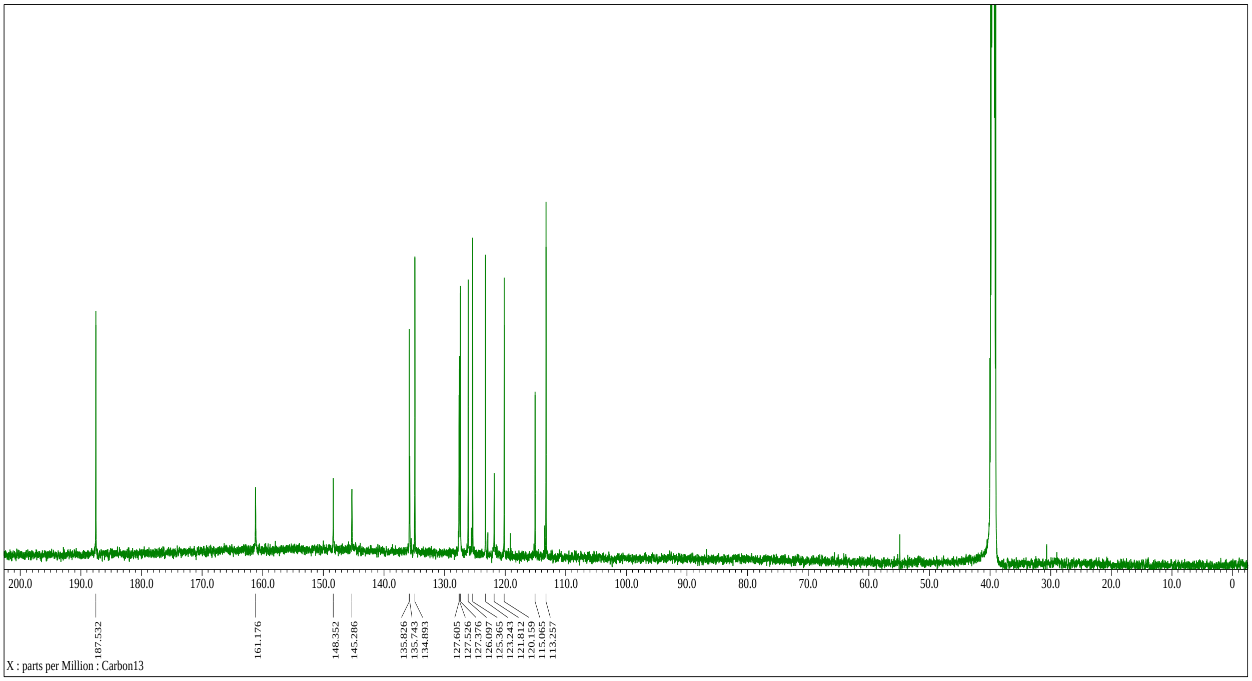

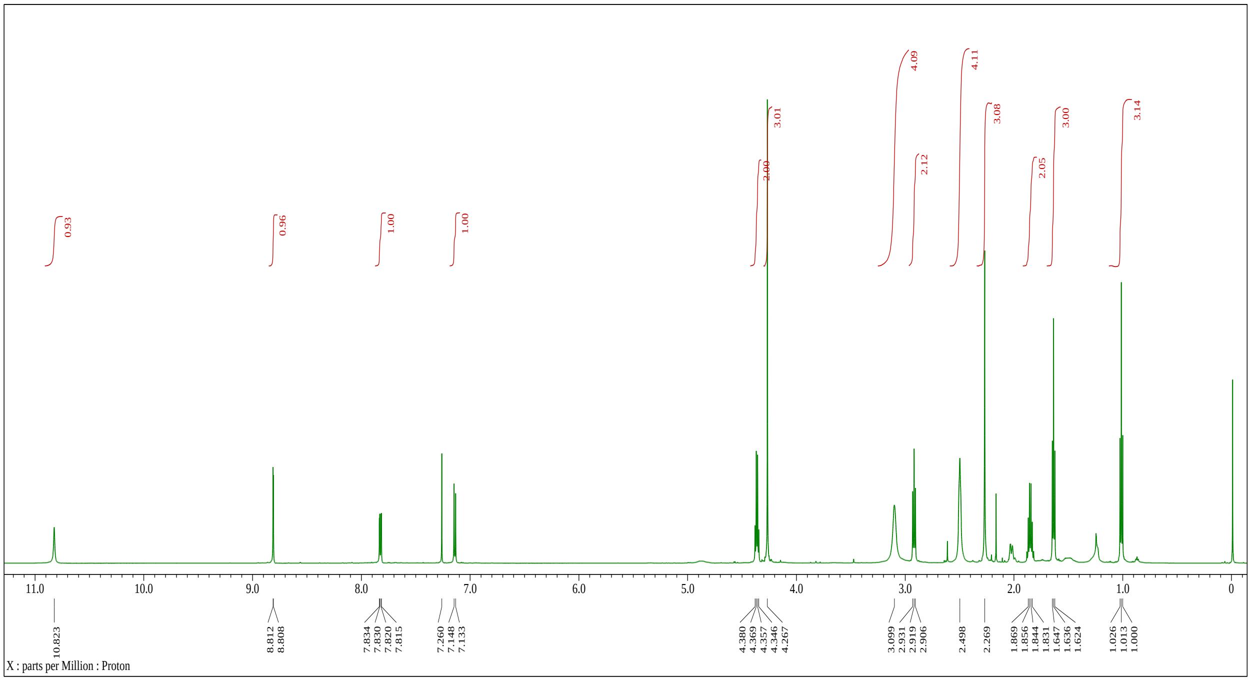
X : parts per Million : Proton

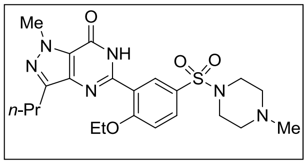
Me
N
N
O
NH
N
n-Pr
EtO
S
O
O
N
N
Me

Sildenafil

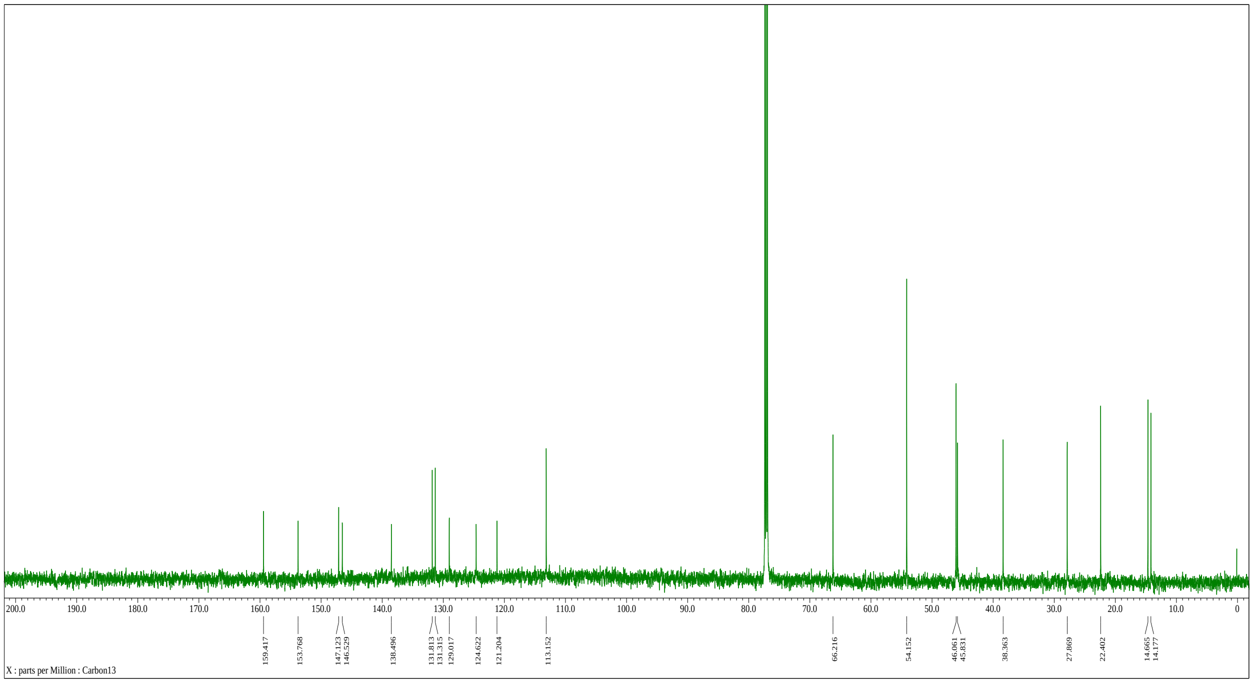
X : parts per Million : Carbon13

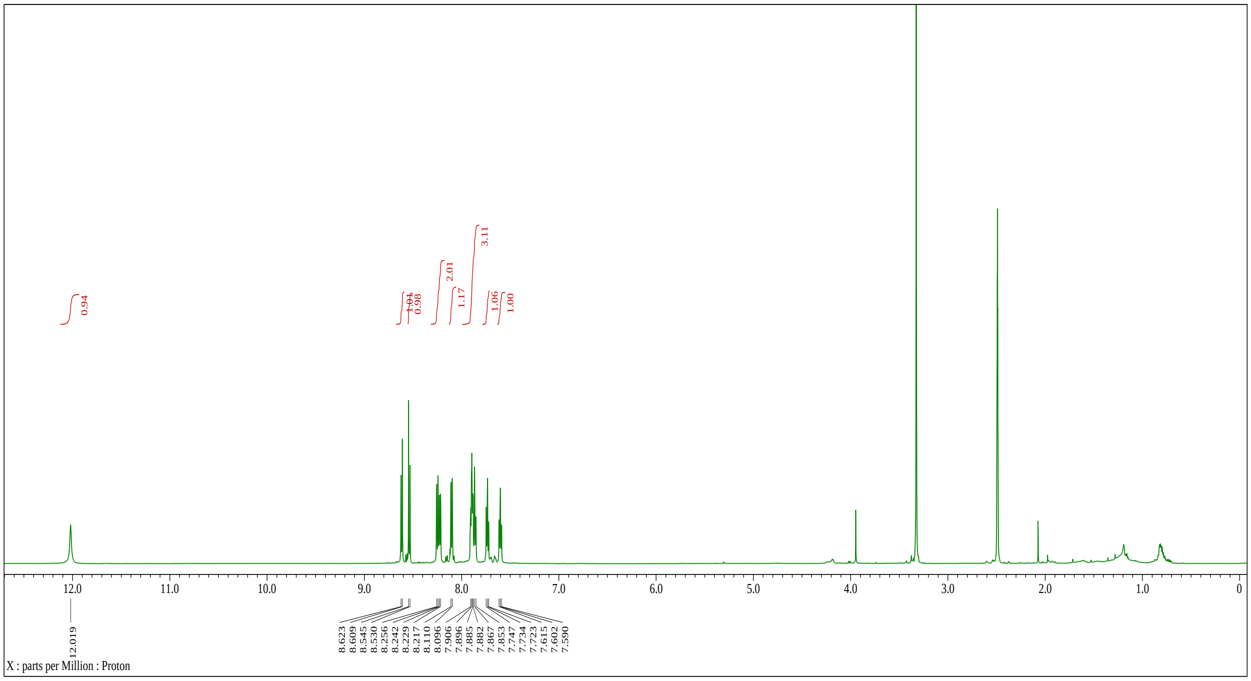

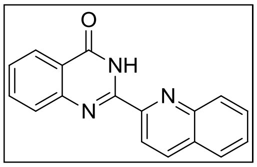

6

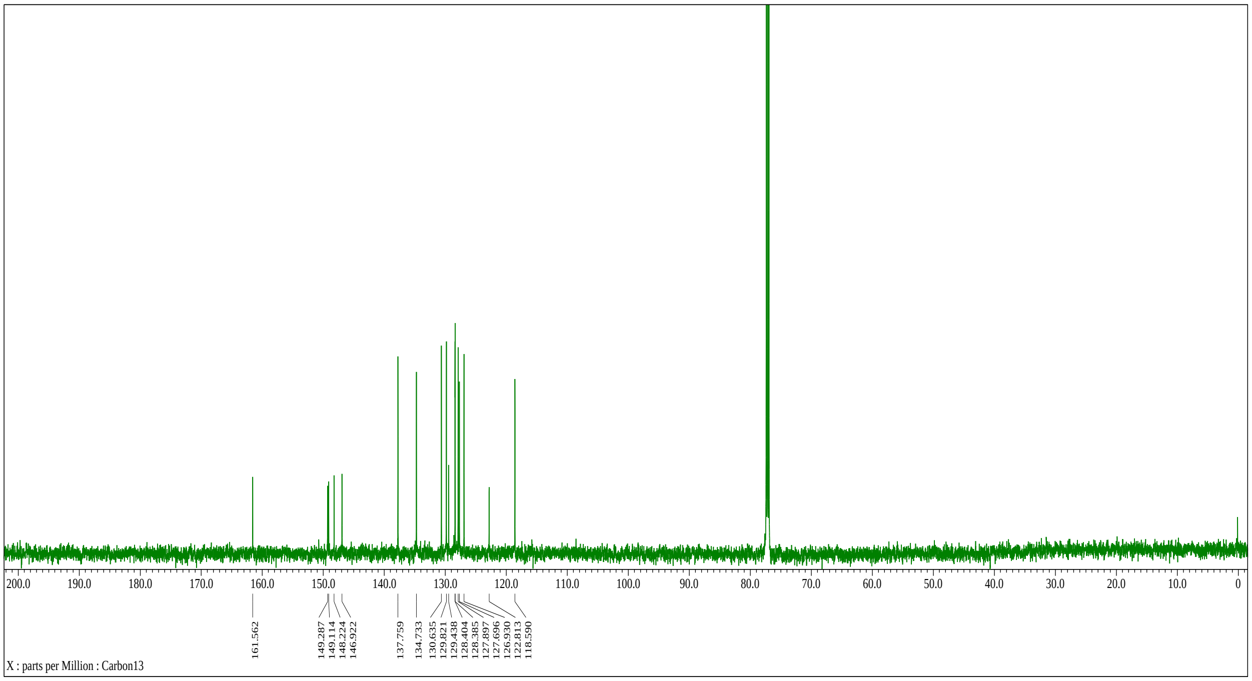

Supplement: RA-010-D0RA06820A-s001 [file RA-010-D0RA06820A-s001.pdf]
